# Validation of the 11^+^Myco MS-PREP^®^ Method for Determination of Aflatoxins, Fumonisins, Deoxynivalenol, Ochratoxin A, Zearalenone, HT-2, and T-2 Toxins in Cereals, Baby Food, Spices, and Animal Feed by Immunoaffinity Column with LC–MS/MS: AOAC *Performance Tested Method*^SM^ 112401

**DOI:** 10.1093/jaoacint/qsae097

**Published:** 2024-12-11

**Authors:** Dave Leeman, Andrew B Allan, Helen Cameron, Carol Donelly, Adam Tramaseur, Joanna Stratton, Susan J MacDonald

**Affiliations:** R-Biopharm Rhône Ltd, Block 10 Todd Campus, West of Scotland Science Park, Acre Rd, Glasgow G20 0XA, UK; R-Biopharm Rhône Ltd, Block 10 Todd Campus, West of Scotland Science Park, Acre Rd, Glasgow G20 0XA, UK; R-Biopharm Rhône Ltd, Block 10 Todd Campus, West of Scotland Science Park, Acre Rd, Glasgow G20 0XA, UK; R-Biopharm Rhône Ltd, Block 10 Todd Campus, West of Scotland Science Park, Acre Rd, Glasgow G20 0XA, UK; Fera Science Ltd, Sand Hutton, York YO41 1LZ, UK; Fera Science Ltd, Sand Hutton, York YO41 1LZ, UK; Fera Science Ltd, Sand Hutton, York YO41 1LZ, UK

## Abstract

**Background:**

The 11^+^Myco MS-PREP^®^ immunoaffinity column (IAC) contains a gel suspension of monoclonal antibodies specific to the toxins of interest. Following sample extraction, the IAC is used for cleanup and concentration of mycotoxins prior to analysis by LC with tandem mass spectrometry (LC–MS/MS).

**Objective:**

This study evaluated the IAC with LC–MS/MS method for *Performance Tested Method*^SM^ certification for simultaneous determination and confirmation of aflatoxins (AF) B_1_, B_2_, G_1_, G_2_, and M_1_; deoxynivalenol (DON), fumonisins B_1_, B_2_, and B_3_; ochratoxin A (OTA); T-2; HT-2; and zearalenone (ZON) from corn, wheat, cereal-based baby food (with and without dairy ingredients), paprika, chili powder, and animal feed.

**Methods:**

A single extraction method using acetonitrile-water (1 + 1, by volume) was used for all matrixes. The method developer validated all matrixes and an independent laboratory verified method performance on corn and animal feed. Data were analyzed for recovery, repeatability precision, LOD, LOQ, confirmation of identity, and method selectivity.

**Results:**

Recovery (72–138%) and repeatability (0.46–24%), with the exception of sporadic data points, were within acceptance criteria. LOQ was estimated as AFB_1_ 0.018–0.32 μg/kg, AFB_2_ 0.037–0.28 μg/kg, AFG_1_ 0.019–0.14 μg/kg, AFG_2_ 0.036–0.28 μg/kg, DON 4.0–75 μg/kg, fumonisin B_1_ 4.9–37 μg/kg, fumonisin B_2_ 4.0–32 μg/kg, fumonisin B_3_ 2.0–16 μg/kg, OTA 0.15–4.4 μg/kg, T-2 0.5–7.5 μg/kg, HT-2 0.70–7.5 μg/kg, and ZON 1.3–7.2 μg/kg, depending on matrix. Method performance was verified with reference and QC materials. Selectivity and confirmation of identity were also demonstrated.

**Conclusion:**

The 11^+^Myco MS-PREP IAC with LC–MS/MS method demonstrated acceptable performance for simultaneous determination of 12 mycotoxins in seven matrixes.

**Highlight:**

The data were reviewed by the AOAC *Performance Tested Methods*^SM^ Program and approval was granted for certification of the 11^+^Myco MS-PREP Method as PTM 112401.

Mycotoxins are toxic metabolites produced by molds, which can contaminate food from field to fork. They can adversely affect food safety through their potential to have serious acute and chronic effects on the health of humans, as well as animals (1–3).

Throughout history, mycotoxins have impaired the quality of agricultural products. Major commodities such as corn, wheat, rice, soy, and peanuts can be contaminated with mycotoxins, depending on growth conditions and storage conditions. Other commodities like rye, oats, hazelnuts, figs, grapes, or spices are also at risk from mycotoxins. The consumption of mycotoxin-contaminated products may have acute toxic health effects on humans and animals as well as chronic effects. Besides health costs, direct economic factors play a role like decreased efficiency in the growth of farm animals and trade losses because of import and export limitations and direct losses in revenue of crop value. Mycotoxin analysis of food and feed is therefore subject to regulatory guidance and legislation (4–21).

There are a variety of different mycotoxins that may even occur simultaneously. For example, cereals may contain different types of trichothecenes (e.g., deoxynivalenol (DON) and T-2/HT-2 toxin), while aflatoxin (AF) is often found together with ochratoxin (OTA) in spices.

Numerous mycotoxins are regulated by law, which means that the concentration in food and feed must not exceed a certain limit value (*see*  Table 1). It is therefore necessary to test the products for all potentially present mycotoxins. To perform efficient multi-mycotoxin analyses, many companies use LC with tandem mass spectrometric detection (LC–MS/MS). LC–MS/MS is particularly suitable for high sample throughput. However, the drawback of this method is that the analysis result is strongly influenced by the matrix, leading to measurement uncertainty. When given appropriate tools, i.e., 11^+^Myco MS-PREP^®^, these so-called matrix effects can be reduced—even for multiple mycotoxins at the same time.

**Table 1.** Regulatory limits for mycotoxins in claimed foods in ng/g (µg/kg, ppb)

Toxin	Cereal (corn, wheat)		Cereal-based baby food		Animal feed		Spices (paprika, chili)		Milk powder/milk	
	EU	US	EU	US	EU	US	EU	US	EU	US
AFB_1_	2–5		0.1		5		5			
AFB_2_										
AFG_1_										
AFG_2_										
Total aflatoxin	4–10	20				20–300	10			
AFM_1_									0.025 (IMF^d^)/0.05	No limit/0.5
DON	400–1750	1000^a^	150		900–12000	5000–10000^a^				
FB_1_										
FB_2_										
FB_3_										
Total fumonisin^b^	800–4000	2000–4000^a^	200		5000–60000	5000–100000^a^				
OTA	3		0.5				15–20			
T-2										
HT-2										
Total T-2 and HT-2	20		10		50–2000^c^					
ZON	50		20		100–3000					

a Guidance Levels.

b EU includes FB_1_ and FB_2_, and US includes FB_1_, FB_2_, and FB_3_.

c Recommendation.

d IMF = Infant milk formula.

Immunoaffinity columns (IAC) are considered the method of choice for the best sample cleanup prior to LC–MS/MS. For the convenient analysis of multiple mycotoxins, a single extraction method for all analytes will greatly improve efficiencies in the laboratory through reduced solvent use and time taken to extract and analyze a given sample. The 11^+^Myco MS-PREP IAC is a highly effective, cost-saving product that can be used with a single extraction method for multi-mycotoxin analysis in a number of commercially important matrixes, eliminating the need for matrix-matched standards.

## Principle Scope of Method

The 11^+^Myco MS-PREP IAC contains a gel suspension of monoclonal antibodies specific to the toxins of interest. Following extraction of the toxins, the extract is filtered, diluted, and passed slowly through the immunoaffinity column. Any toxins present in the sample are retained by the antibodies within the gel suspension. The column is washed to remove unbound material and the toxins are then released from the column following elution with solvent. The eluate is collected prior to analysis by LC–MS/MS.

The total extraction and cleanup takes approximately 60 min to perform and multiple samples can be processed simultaneously. The result is improved cleanup and concentration of the toxins from food and feed samples, reducing ion suppression and removing the need for matrix-matched standards. This provides cleaner chromatography, improved sensitivity, and greater accuracy. The columns also have the added advantage that they can be automated for large-scale analysis of samples.

A 5 g test portion of ground sample is subjected to a acetonitrile-water (1 + 1, by volume) extraction by shaking under controlled conditions. Following filtration and dilution in aqueous buffer, the diluted extract may be further filtered before being added directly to the 11^+^Myco MS-PREP IAC. The prepared extract is then allowed to pass through the IAC at approximately 2 mL/min where any toxins present in the sample are retained by the antibodies within. Unbound material is then washed from the column using an ammonium acetate buffer. Finally, the captured toxins are released from their antibodies by an elution process using 100% methanol that is completed by an equivalent volume of deionized water.

The LC–MS/MS analysis employs a gradient LC method with electrospray ionization in positive polarity using scheduled multiple reaction monitoring (sMRM). For each batch run, a calibration curve is analyzed before and after the sample injections. A mid-point standard is injected several times (≥6) before the first calibration curve and at regular intervals amongst the samples. Diluent injections are included at the start of the sequence as well as before and after the calibration curves to check for carryover.

## Scope of Study

- *Analytes.*—Aflatoxins (B_1_, B_2_, G_1_, G_2_, and M_1_), fumonisins (B_1_, B_2_, and B_3_), deoxynivalenol, ochratoxin A, zearalenone (ZON), HT-2, and T-2 toxins.
- *Matrixes.*—Corn, wheat, cereal-based baby food (with and without dairy ingredients), paprika, chili powder, and animal feed
- *Summary of validated performance claims*.—The 11^+^Myco MS-PREP immunoaffinity column method meets OMA (*see*  Table 6) and Horwitz Ratio (HorRat) acceptance criteria (19) for the simultaneous determination and confirmation of aflatoxins B_1_, B_2_, G_1_, G_2_, and M_1_; deoxynivalenol, fumonisins B_1_, B_2_, and B_3_; ochratoxin A; T-2; HT-2; and zearalenone from a single test portion.

**Table 6.** Recovery acceptance criteria^a^

Analyte, %	Mass fraction (C)	Unit	Mean recovery, %
100	1	100%	98–102
10	10^–1^	10%	
1	10^–2^	1%	97–103
0.1	10^–3^	0.1%	95–105
0.01	10^–4^	100 ppm (mg/kg)^b^	90–107
0.001	10^–5^	10 ppm (mg/kg)	80–110
0.0001	10^–6^	1 ppm (mg/kg)	
0.00001	10^–7^	100 ppb (μg/kg)^c^	
0.000001	10^–8^	10 ppb (μg/kg)	60–115
0.0000001	10^–9^	1 ppb (μg/kg)	40–120

a *Official Methods of Analysis of AOAC INTERNATIONAL*, 22nd Edition Appendix F, Table A5. Table excerpted from AOAC Peer-Verified Methods Program, Manual on Policies and Procedures (1998) AOAC INTERNATIONAL, Rockville, MD, USA. Recovery is defined as the ratio of the observed mean test result to the true value. The range of the acceptable mean recovery expands as the concentration of the analyte decreases. This table provides target mean recovery ranges for analyte concentrations from 100% to 1 ppb.

b ppm = Parts per million.

c ppb = Parts per billion.

## Materials and Methods

### Test Kit Information

- *Kit name*.—11^+^Myco MS-PREP
- *Cat. No*.—RBRP128 or RBRP128B
- *Ordering information*.—R-Biopharm Rhône, Ltd, Block 10 Todd Campus, West of Scotland Science Park, Acre Rd, Glasgow, UK G20 0XA. www.r-biopharm.com. Phone: +44 (0) 141 945 2924. E-Mail: info@r-biopharmrhone.com

### Test Kit Components

- *Immunoaffinity columns.*—10 (RBRP128) or 50 (RBRP128B).

### Additional Supplies and Reagents

- *Accessory pack*.—Cat. No. AP01
- *Column rack*.—Cat. No. CR1
- *Water*.—Deionized.
- *Methanol (MeOH)*.—LC-MS grade.
- *Acetonitrile (ACN)*.—HPLC grade.
- *Phosphate-buffered saline (PBS)*.—Prepare from 10× tablets (Sigma-Aldrich Co. Ltd, Dorset, UK, Cat. No. P4417) per manufacturer’s instructions.
- *Ammonium acetate (CH_3_CONH_4_)*.—Certified ACS grade.
- *Sodium chloride (NaCl)*.—Certified ACS grade.
- *Ammonium formate (HCOONH_4_)*.—LC-MS grade.
- *Formic acid*.—LC-MS grade.
- *Tween 20*.—Sigma-Aldrich, Cat. No. P1379
- *Filter paper*.—Whatman No. 113 or No. 4 and glass microfiber.
- *Centrifuge tubes*.—50 mL conical polypropylene tubes with screw caps.
- *NH_4_OAc solution (20 mM)*.—Weigh 1.54 g NH_4_OAc into a 1 L volumetric flask. Bring to volume with water and stir to dissolve. Prepare fresh each day of analysis.
- *PBS with 0.1% Tween 20 solution (PBS-T)*.—Weigh 1.0 g Tween 20 into a volumetric flask. Bring to volume with PBS and stir to disperse the Tween 20. Store at room temperature. Prepare fresh weekly.
- *Mobile phase A (1 mM ammonium formate [HCOONH_4_] and 0.1% formic acid in 5% MeOH)*.—For 1 L, weigh 0.063g HCOONH_4_ and dissolve in 950 mL deionized water. Add 1 mL formic acid (LC-MS grade) and 50 mL MeOH (LC-MS grade) and mix well.
- *Mobile phase B (1 mM HCOONH_4_ and 0.1% formic acid in 98% MeOH)*.—For 1 L, weigh 0.063g HCOONH_4_ and dissolve in 20 mL deionized water. Add 1 mL formic acid (LC-MS grade) and 980 mL MeOH (LC-MS grade), mix well.

### Apparatus

- *Orbital shaker*.—KS 501 Digital, IKA (Wilmington, NC), Cat. No. 0025004451.
- *Balances*.—To an accuracy of two decimal places 0.1 g to 4100 g, three decimal places 100 mg to 300 g, and four decimal places 10 mg to 50 g.
- *Pipettes.*—Adjustable to 5–50 µL, 200–200 µL, 100–1000 µL, and 500–5000 µL.
- *Volumetric flasks*.—Calibrated to 10 ± 0.025 mL and 100 ± 0.1 mL.
- *Graduated cylinders*.—With capacities of 1 L, 500 mL, 100 mL, and 50 mL.
- *Centrifuge*.—With rotor to hold 50 mL centrifuge tubes.
- *LC–MS/MS*.—Agilent 1260 Infinity II series LC, G7112B binary pump, G7129A Vialsampler, and G7116A MCT column oven. QTRAP 5500 MS/MS (SCIEX, Framingham, MA); Analyst software version 1.7.2, Analyst Device Driver, and MultiQuant version 3.0.3, or equivalent system.
- *Chromatographic column*.—Luna Omega 3 µm Polar C18, 100 × 3 mm, Phenomenex (Torrance, CA). Cat. No. 00D-4760-Y0.

### Standard Solutions

- *Aflatoxin B_1_ (AFB_1_)*.—25 µg/mL in ACN, Trilogy Analytical Laboratory (Washington, MO) Cat. No. TAS-M11LA1-10.
- *Aflatoxin B_2_ (AFB_2_)*.—25 µg/mL in ACN, Trilogy Cat. No. TAS-M12LA1-10.
- *Aflatoxin G_1_ (AFG_1_)*.—25 µg/mL in ACN, Trilogy Cat. No. TAS-M13LA1-10.
- *Aflatoxin G_2_ (AFG_2_).*—25 µg/mL in ACN, Trilogy Cat. No. TAS-M14LA1-10.
- *Aflatoxin M_1_ (AFM_1_)*.—1 µg/mL in ACN, Trilogy Cat. No. TAS-M15LA2-2.
- *Deoxynivalenol (DON)*.—100 µg/mL in MeOH, Trilogy Cat. No. TAS-M21LM2-10.
- *Total fumonisins (FUM)*.—250 µg/mL fumonisin B_1_, 125 µg/mL fumonisin B_2_, 62.5 µg/mL fumonisin B_3_ in 50% ACN, Trilogy Cat. No. TAS-MM19LZ2-2. *Note*: The ratio of fumonisins B_1_, B_2_, and B_3_ in purchased standards varies. Please note the correct ratio for the purchased standard.
- *Ochratoxin A (OTA)*.—10 µg/mL in MeOH, Trilogy Cat. No. TAS-M16LM2-5.
- *T-2 toxin*.—100 µg/mL in ACN, Trilogy Cat. No. TAS-M24LA1-5.
- *HT-2 toxin*.—100 µg/mL in ACN, Trilogy Cat No. TAS-M25LA1-5.
- *Zearalenone (ZON)*.—100 µg/mL in MeOH, Trilogy Cat. No. TAS-M17LM2-10.

### Safety Precautions

Personal protective equipment must be worn (i.e., lab coat, safety goggles, gloves). Concentrations of mycotoxins above 1 µg/mL must be handled in a fume hood. Avoid prolonged exposure. Staff should be trained to work with mycotoxins. Contaminated solutions and food samples must be decontaminated before disposal and all re-useable glassware/labware too, prior to the normal cleaning process.

### General Preparation

#### Preparation of calibration standards

- *Total aflatoxin (AFLA) stock solution (2500 ng/mL, aflatoxins B_1_: B_2_: G_1_: G_2_, 1:1:1:1).*—Adjust volumes as needed depending on standard concentrations.
- *Total T-2 & HT-2 stock solution (≈100* *000 ng/mL, T-2: HT-2, 1:1)*.—Adjust volumes as needed depending on standard concentrations.
- *Combined working standard 1 (including aflatoxin M_1_)*.—Prepare a combined working standard solution according to Supplemental Table 1. Adjust volumes as needed depending on standard concentrations.
- *Combined working standard 2 (without aflatoxin M_1_)*.—Prepare a combined working standard solution according to Supplemental Table 2, but substitute MeOH for the volume of aflatoxin M_1_ added. Adjust volumes as needed depending on standard concentrations.
- *Calibration curve standards.*—Prepare solutions for a 7-point calibration curve by serial dilution. Use combined working standard 1 or 2 depending on whether aflatoxin M_1_ is required for the analysis. Final concentrations of toxins in each standard are shown in Supplemental Table 3.
  1. *Standard 7 (Std7).—*Dilute 1 mL combined working standard 1 or 2 with 9 mL 50% MeOH.
  2. *Standard 6 (Std6).—*Dilute 1 mL Std 7 with 1 mL 50% MeOH.
  3. *Standard 5 (Std5).—*Dilute 1 mL Std 6 with 1 mL 50% MeOH.
  4. *Standard 4 (Std4).—*Dilute 1 mL Std 5 with 2 mL 50% MeOH.
  5. *Standard 3 (Std3).—*Dilute 1 mL Std 4 with 2 mL 50% MeOH.
  6. *Standard 2 (Std2).—*Dilute 1 mL Std 3 with 2 mL 50% MeOH.
  7. *Standard 1 (Std1).—*Dilute 1 mL Std 2 with 2 mL 50% MeOH.

### Sample Preparation

Follow one of the officially recognized sampling procedures to obtain a representative sample.

It is recommended that a minimum of 1 kg of representative sample is finely ground (21).

#### Cereal and cereal based baby food

- Weigh 5 g ground sample into a 50 mL centrifuge tube.
- Add 20 mL acetonitrile-water (1 + 1, by volume), cap, shake by hand until sample is suspended in solution then shake for 30 min at 300 revolutions per minute (rpm) on an orbital shaker.
- Filter the extract through Whatman No. 113 or No. 4 filter paper. Alternatively, centrifuge the extract, at approximately 3000*g* for 10 min.
- For cereals, dilute 2 mL clarified extract in 48 mL PBS. Filter diluted extract through glass microfiber filter paper.
- For cereal-based baby food, dilute 4 mL clarified extract in 46 mL PBS. If cloudy, filter diluted extract through glass microfiber filter paper.
- Pass 20 mL diluted extract through the IAC at approximately 2 mL/min.
- Wash the column by passing 20 mL 20 mM ammonium acetate through at a flow rate of approximately 5 mL/min. Pass air through the column to remove any residual liquid.
- Elute toxins from the column at a flow rate of 1 drop/s using 1.5 mL MeOH with backflushing and collect the eluate into a clean amber glass vial. (Backflush the column by gently raising and lowering the syringe plunger on the glass barrel connected to the IAC, perform three cycles of forward and backward flow, then elute the toxins at a flow rate of 1 drop/s.) Following MeOH elution, pass 1.5 mL water through the column and collect in the same vial to give 3 mL total volume. Cap the vial and mix.
- Inject 20 µL onto the LC–MS/MS system for analysis.

#### Animal feed

- Weigh 5 g ground sample into a 50 mL centrifuge tube.
- Add 20 mL acetonitrile-water (1 + 1, by volume), cap, shake by hand until sample is suspended in solution and shake for 30 min at 300 rpm on an orbital shaker.
- Filter the extract through Whatman No. 113 or No. 4 filter paper. Alternatively, centrifuge the extract, at approximately 3000*g* for 10 min.
- Dilute 1 mL clarified extract in 49 mL PBS-T. Filter diluted extract through glass microfiber filter paper.
- Pass 20 mL diluted extract through the IAC at approximately 2 mL/min.
- Wash the column by passing 20 mL 20 mM ammonium acetate through at a flow rate of approximately 5 mL/min. Pass air through the column to remove any residual liquid.
- Elute toxins from the column at a flow rate of 1 drop/s using 1.5 mL MeOH with backflushing and collect the eluate into a clean amber glass vial. (Backflush the column by gently raising and lowering the syringe plunger on the glass barrel connected to the IAC, perform three cycles of forward and backward flow, then elute the toxins at a flow rate of 1 drop/s.) Following MeOH elution, pass 1.5 mL water through the column and collect in the same vial to give 3 mL total volume. Cap the vial and mix.
- Inject 10 µL onto the LC–MS/MS system for analysis.

#### Spices

- Weigh 5 g ground sample into a 50 mL centrifuge tube.
- Add 20 mL acetonitrile-water (1 + 1, by volume), cap, shake by hand until sample is suspended in solution and shake for 30 min at 300 rpm on an orbital shaker.
- Filter the extract through Whatman No. 113 or No. 4 filter paper. Alternatively, centrifuge the extract, at approximately 3000 *g* for 10 min.
- Dilute 2 mL clarified extract in 23 mL PBS-T. Filter diluted extract through glass microfiber filter paper.
- Pass 5 mL diluted extract through the IAC at approximately 2 mL/min.
- Wash the column by passing 20 mL 20 mM ammonium acetate through at a flow rate of approximately 5 mL/min. Pass air through the column to remove any residual liquid.
- Elute toxins from the column at a flow rate of 1 drop/s using 1.5 mL MeOH with backflushing and collect the eluate into a clean amber glass vial. (Backflush the column by gently raising and lowering the syringe plunger on the glass barrel connected to the IAC, perform three cycles of forward and backward flow, then elute the toxins at a flow rate of 1 drop/s). Following MeOH elution, pass 1.5 mL water through the column and collect in the same vial to give 3 mL total volume. Cap the vial and mix.
- Inject 20 µL onto the LC–MS/MS system for analysis.

### Analysis

- *LC conditions.*—Column temperature, 40°C; flow rate, 0.6 mL/min; method developer injection volume 20 µL for cereal, cereal-based baby food, and spices, 10 µL for animal feed, independent laboratory injection volume 10 µl for cereal and animal feed; gradient: 0.0 min 40% mobile phase B, 0.5 min 40% mobile phase B, method developer 4.0 min 100% mobile phase B, 6.0 min 100% mobile phase B, independent laboratory 4.0 min 99% mobile phase B, 6.0 min 100% mobile phase B, 6.1 min 40% mobile phase B, 9.0 min 40% mobile phase B.
- *MS/MS conditions.*—*See*  Supplemental Tables 4 and 5 for MS/MS settings.
- *Injection sequence.*—For each batch, follow the sequence: two injections standard diluent; six injections Std4, one injection standard diluent; one injection each Std1–Std7; one injection standard diluent; one injection each of up to 12 samples; one injection Std4; continue sample injections with Std4 injected after up to 12 samples; after last set of samples, one injection standard diluent, one injection each Std1–Std7; and two injections standard diluent. Standard diluent injections allow for carryover checks.

### Calculations, Interpretation, and Test Result Report

- *Calibration curve.*—Plot peak areas against analyte concentration from the two calibration standard sequence injections. Perform regression analysis. For most cases, a linear regression with 1/*x* or 1/*x*^2^ weighting will provide a good fit. For some analytes, a quadratic regression may be necessary with weighting. In either case, use the least weighting necessary (1/*x* versus 1/*x*^2^) to achieve system suitability. MultiQuant software will perform this function.
- *Analyte concentration in unknowns.*—Compare the peak areas from the unknowns to the calibration curve to determine the concentration of analyte in the injected IAC eluates in ng. Analyst software will perform this function. To calculate the mycotoxin concentration in the matrix (ng/g), multiply the IAC eluate concentration (ng) by the conversion factor from Supplemental Table 6.
- *System suitability: repeatability*.—For each analyte, calculate the repeatability (RSD_r_) from the areas of the last six Std4 injections. The RSD_r_ should be ≤6%.
- *System Suitability: calibration standards.*—For each analyte calibration curve, the linearity coefficient R must be ≥0.99. For each calibration standard in the injection sequence, the accuracy should be 80–120% (accuracy in % = 100 × calculated concentration [ng]/expected concentration [ng]). Analyst software determines the linearity coefficient R and accuracy of the calibration standards.
- *Determination of precision*.—The precision requirements were derived from the Horowitz equation as per Codex CXS 193-1995, Revision 2023 (16). Codex estimates the predicted repeatability as 66% of predicted reproducibility and allows up to 2× the predicted value (HorRat ≤2). The *Codex Procedural Manual* recommends capping Predicted Relative Standard Deviation of Reproducibility (PRSD(R)) at 22% when the concentration goes below 10e-7 (100 μg/kg), which corresponds to a maximum allowed repeatability of 29% (based on HorRat ≤2).

### Confirmation

For each analyte where quantitative and qualitative transition peaks are present, calculate the area ratios of the qualifier to the quantifier transitions. Determine the mean ion ratios of the calibration curve standards. These are the expected ratios used for confirmation of identity. Calculate the qualifier/quantifier ion ratios for each analyte in the unknown samples. The identity of an analyte is confirmed if the qualifier/quantifier ion ratio is within ±30% of the expected ion ratio. Analyst software will determine the ion ratios. The retention time of analyte peaks in the unknowns must be within 0.2 min of the mean retention time of the standards.

### Experimental

This validation study was conducted under the AOAC Research Institute (RI) *Performance Tested Methods* program. Method developer studies were conducted in the laboratories of R-Biopharm Rhône Ltd and included the selectivity study, calibration model evaluation, matrix studies for all claimed matrixes, product consistency and stability, and robustness testing. The independent laboratory study was conducted by Fera Science Ltd and included a matrix study for two matrixes.

### Method Developer Studies

####  

- *Calibration study.—*Solvent-based calibration curves were generated according to the 11^+^Myco MS-PREP method. In addition, matrix extracts were prepared from samples of each matrix processed through the IAC cleanup. The IAC eluants were used as diluents for preparation of matrix-matched standard curves. All curves were analyzed by regression, residual analysis, and comparison of slopes between solvent-based and matrix-matched for each analyte. A matrix effect is observed as a ≥20% shift in the calibration curve slope.
- *Selectivity study.—*Four non-analyte compounds were tested for potential interference, including nivalenol (100 µg/mL in MeOH, Trilogy Cat. No. TAS-M28LM1-5), neosolaniol (100 µg/mL in ACN, Trilogy Cat. No. TAS-M27LA1-5), sterigmatocystin (50 µg/mL in ACN, Sigma Cat. No. 32986), and α-zearalenol (10 µg/mL in ACN, Sigma Cat. No. 35406). Each non-analyte was tested at 10× the concentration of the structurally similar analyte in the highest calibration standard (Std7). Note that 10× AFLA and 10× the sum of T-2 and HT-2 were used for sterigmatocystin and neosolaniol, respectively. Corn matrix was extracted and filtered according to the candidate method. Two mL aliquots of filtrate received 0 or 0.5 mL Std7 and 0 or 0.5 mL of a non-analyte solution (60 ng/mL sterigmatocystin, 3750 ng/mL nivalenol, 800 ng/mL neosolaniol, or 750 ng/mL α-zearalenol) prepared in MeOH: PBS, 50:50 v/v. All aliquots were then brought to 50 mL with PBS. Twenty mL of the resulting solution was processed through the IAC and analyzed by LC–MS/MS.
- *Matrix study.—*The matrix study was conducted on all claimed matrixes (corn, wheat, cereal-based baby food [with and without dairy ingredients], paprika, chili powder, and animal feed) to determine bias, recovery, repeatability precision, estimated LOD (LOD_est_), and LOQ. Matrixes were obtained from local vendors or online purchases. In addition, various QC materials for corn, wheat, infant food powder, animal feed, dry dog food (cereal-based), chili powder, paprika powder, and whole milk powder were procured (*see*  Supplemental Table 7). The testing of each matrix was conducted on a single day by one analyst on one LC–MS/MS system with six replicates per spike level including blank material (i.e., 6 replicates × 4 levels = 24 test portions per matrix). As European Union Legislative Levels (EU LL) are lower than US Maximum Limits (ML) or Guidance Levels (GL), spike levels were based on EU LL (*see*  Table 1 for comparison of US and EU regulations). Aflatoxin M_1_ is included in claimed cereal-based baby food where it contains dairy ingredients. There were two cereal-based baby foods tested, one with dairy and one without.
  Spike solutions were prepared using MeOH, ACN, and deionized water. For each analyte in a given matrix, three levels of spike were prepared covering a range below and above legislative levels. For all standard preparations, a vortex mixer was used to mix.
  An aflatoxins B_1_, B_2_, G_1_ and G_2_ stock standard solution (20 000 ng/mL total aflatoxin, 1:1:1:1) was prepared using HPLC grade ACN as a diluent. For example, aflatoxin B_1_ (1938 µL 25 800 ng/mL), aflatoxin B_2_ (2101 µL 23 800 ng/mL), aflatoxin G_1_ (1938 µL 25 800 ng/mL), and aflatoxin G_2_ (1961 µL 25 500 ng/mL) were mixed with 2062 µL ACN to yield a 10.0 mL stock solution with each aflatoxin at 5000 ng/mL and a total aflatoxin concentration of 20 000 ng/mL. Volumes of individual toxins were adjusted as needed based on the supplied concentration. Intermediate stock solutions were prepared as needed by dilution of the stock solution in ACN.
  Cereal spike solution diluent consisted of 85.7% MeOH, 8.9% ACN, and 5.4% H_2_0. Cereal spike solution level 3 was prepared by mixing appropriate volumes of each stock or intermediate stock solution to create a solution containing 500 ng/mL AFLA, 37.5 µg/mL DON, 50.0 µg/mL FUM, 150 ng/mL OTA, 1.0 µg/mL T-2+HT-2 (1:1), and 5.0 µg/mL ZON in MeOH. Two 2-fold serial dilutions in spike diluent were made to create cereal spike solution levels 2 and 1.
  For cereal-based baby food containing dairy ingredients, the spike solution diluent consisted of 96.75% MeOH, 2.19% ACN, and 1.06% H_2_O. The level 3 spike solution was prepared by mixing appropriate volumes of stock or intermediate stock solutions to create a solution containing 20 ng/mL AFLA, 5 ng/mL aflatoxin M_1_, 10.0 µg/mL DON, 10.0 µg/mL FUM, 25 ng/mL OTA, 500 ng/mL T-2+HT-2 (1:1), and 1.0 µg/mL ZON in MeOH. Two 2-fold serial dilutions in spike diluent were made to create cereal spike solution levels 2 and 1.
  For cereal-based baby food without dairy ingredients, the spike solution diluent consisted of 97.24% MeOH, 1.68% ACN, and 1.08% H_2_O. The level 3 spike solution was prepared by mixing appropriate volumes of stock or intermediate stock solutions to create a solution containing 20 ng/mL AFLA, 10.0 µg/mL DON, 10.0 µg/mL FUM, 25 ng/mL OTA, 500 ng/mL T-2+HT-2 (1:1), and 1.0 µg/mL ZON in MeOH. Two 2-fold serial dilutions in spike diluent were made to create cereal spike solution levels 2 and 1.
  For animal feed, spike diluent consisted of 61.5% MeOH, 27.8% ACN, and 10.6% H_2_O. The level 3 spike solution for animal feed consisted of 984 ng/mL AFLA, 44.3 µg/mL DON, 98.4 µg/mL FUM, 1.23 µg/mL OTA, 12.3 µg/mL T-2+HT-2 (1:1), and 4.9 µg/mL ZON in MeOH. Animal feed spike solution level 2 was prepared by mixing 2364 µL level 3 with 736 µL diluent in an amber glass vial. Level 1 was prepared by mixing 1000 µL level 2 with 2000 µL diluent.
  Diluent for spices consisted of 94.4% MeOH, 4.6% ACN, and 1.0% H_2_O. The level 3 spike solution for spices consisted of 500 ng/mL AFLA, 5.01 µg/mL DON, 9.98 µg/mL FUM, 1.0 µg/mL OTA, 1.0 µg/mL T-2+HT-2 (1:1), and 2.5 µg/mL ZON in MeOH. Two 2-fold serial dilutions in spike diluent were made to create cereal spike solution levels 2 and 1.
  For each matrix on Day 1, for each of the three spike levels and the unfortified blank material, one or more analysts weighed 6× 5 g test material into 50 mL centrifuge tubes. Samples were blind-coded and fortified with spike solutions. For each spike level, 200 µL of the appropriate spike solution was pipetted onto the surface of the test portion, with the exception of level 3 for animal feed in which 610 µL spike solution was used. Samples were then fully randomized and labelled with a code by a different analyst. The spiked test portions were left uncovered, in the dark, overnight to allow solvent to evaporate. A time of 18 h was preferred to help fit into the working day. On Day 2, the samples were processed as per the method.
- *Solvent evaporation after spiking.—*Prior to the validation, the evaporation of solvent was verified by weighing matrix test portions before spiking, after spiking, and after evaporation. While most of the spike sample diluent (SSD) evaporated, there was still some residual non-evaporated SSD left after 24 h in all cases.
  There was less evaporation with the samples where the SSD had a higher water content, (corn flour, wheat, and animal feed), than for SSDs with a lower water content (cereal-based baby food, dairy and non-dairy, paprika, and chili powder). Results showed an increase in weight of the non-dairy baby food over time, which is assumed to be due to absorbing moisture from the atmosphere. This effect over both 18 and 24 h was not seen with the other samples and led to a slightly anomalous increase in non-evaporated SSD weight from 18 to 24 h in this sample. We do not believe that the residual amounts observed would have any significant impact on the volume or composition of the extraction solvent. As there was little difference in evaporation weights at 18 or 24 h, 18 h was deemed the more suitable time to leave overnight as this allowed spiking in the afternoon and extraction early the next morning fitting better into the working day. This approach was determined as more practical for laboratory analysis (*see*  Supplemental Figures 5 and 6).
- *LOD and LOQ.—*The initial plan for LOD and LOQ determination was to graph the standard deviation (s_r_) versus observed mean according to Currie (22). However, it was observed that the intercept was often a negative value or above the lowest quantifiable standard. Therefore, it was decided to spike at levels corresponding largely to around calibration standard 1 for the matrixes (for some analytes at a level corresponding to calibration standard 2 with animal feed). In cases where analyte(s) was present at levels greater than LOQ, the matrix was not spiked with that analyte in method developer studies. The independent laboratory study spiked all analytes and used native analyte levels as a baseline for recovery calculations. Given that LOQ estimates were defined as 3 × LOD with respect to linear regression, the equation rearranged was used to give an estimated LOD by dividing the measured LOQ by 3. In some cases where native mycotoxins were present, the true LOQ and LOD would be lower.
- *Product consistency and stability.—*This study examined three lots of 11^+^Myco MS-PREP columns for lot-to-lot variability and product stability in a combined study. Three lots were selected, one near the expiration date Lot JL 334, (24 months after manufacture), one near the middle of the expiration period (Lot LC 611, 8 months after manufacture), and one recently manufactured (Lot LK 718, 4 weeks after manufacture). Three columns from each lot were tested for recovery of analyte from Food Analysis Performance Assessment Scheme (FAPAS) corn TCL0406QC reference material.
- *Robustness study.—*This study evaluated the ability of the method to remain unaffected by small variations in method parameters that might be expected to occur when the method is performed by an end user. Extraction time (30 ± 5 min), column loading flow rate (1–3 drops/s), and column elution flow rates (0.5–2 drops/s) were evaluated in the study using a factorial design of experiment. Two replicates of FAPAS sample TCL0406QC were tested for each treatment combination. The mean and s_r_ for each analyte and each treatment combination were determined and data were subjected to Analysis of Variance (ANOVA) analysis using Minitab software.
- *MS confirmation.—*Confirmation of identity is accomplished by comparing the product ions measured in the samples to those present in the standard injections in both mass and relative intensity. US and EU criteria are slightly different. For the United States, using the mean relative abundance percentages of the standards, acceptance ranges are calculated as mean ±20% arithmetic difference for each ion. For example, at 60% relative abundance, the acceptance range would be 60 ± 20 = 40 to 80%. For the EU, using the mean ion abundance percentages (IAP) of the standard solutions, acceptance ranges are calculated as follows: if IAP >50%, the tolerance is ±20% relative to mean IAP of standards; if IAP >20 to 50%, the tolerance is ±25% relative to mean IAP of standards; if IAP >10 to 20%, the tolerance is ±30% relative to mean IAP of standards; and if IAP = 10%, the tolerance is ±50% relative to mean IAP of standards. For example, at 60% mean IAP of the standards, the sample acceptance criterion would be 60 ± (60 **×** 0.2) = 48 to 72%. The IAP values for each analyte at each level of each matrix were compared to the mean IAP of the standards from the same batch analysis (17,18).
- *Column capacity.—*Capacity challenge solution was prepared for each toxin in acetonitrile: water: PBS (2:2:96 v/v/v) to mimic the cereal sample preparation (refer to Supplemental Figure 7).

### Independent Laboratory Studies

- *Matrix studies.—*The independent laboratory matrix study was conducted to verify the bias, recovery, precision, LOD_est_, and LOQ of the method using fortified materials and commercial QC materials for corn and animal feed. Samples used for the validation were a combination of commercially available food materials obtained for spiking and QC/reference samples for analysis as is. The testing of each matrix was conducted on a single day by one analyst on one LC–MS/MS system with six replicate test portions per fortification level including blank material (i.e., 6 replicates × 4 levels = 24 test portions per matrix). As European Union Legislative Levels (EU LL) are lower than US Maximum Limits (ML) or Guidance Levels (GL), fortification levels are based on EU LL. Spiked matrixes were prepared as described in the *Method Developer* section.

**Figure 1.**
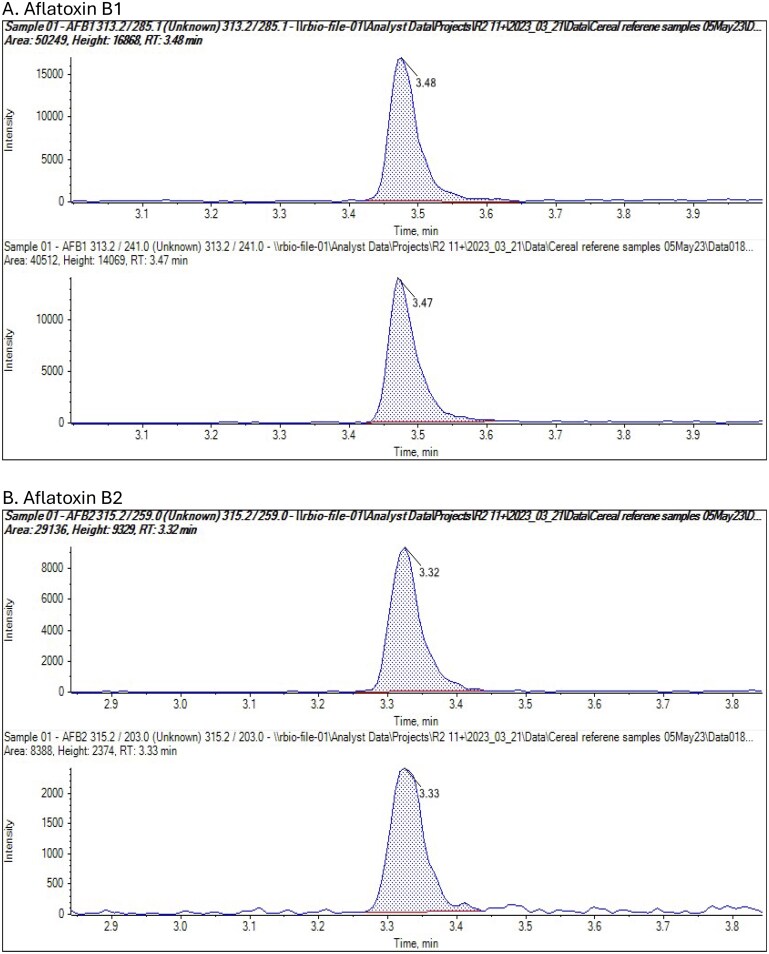

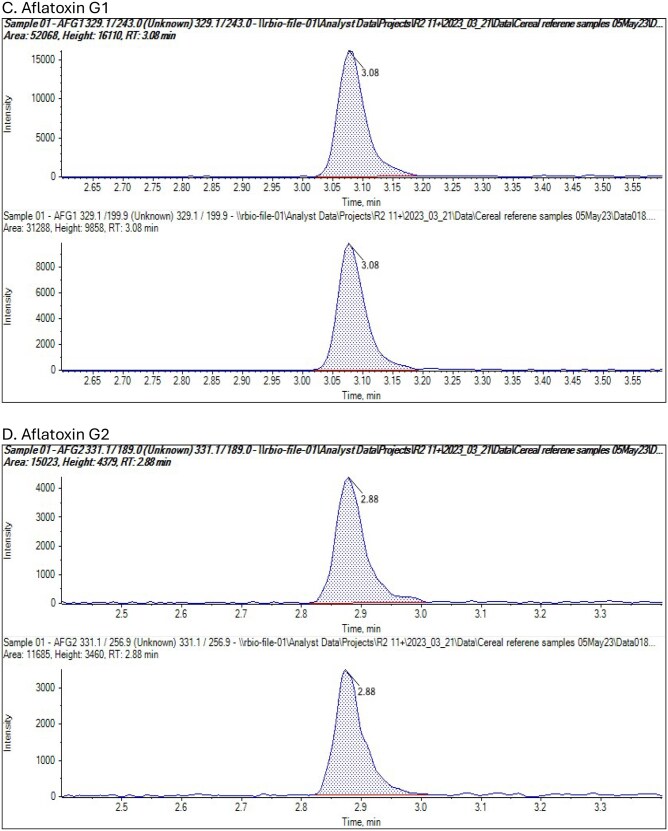

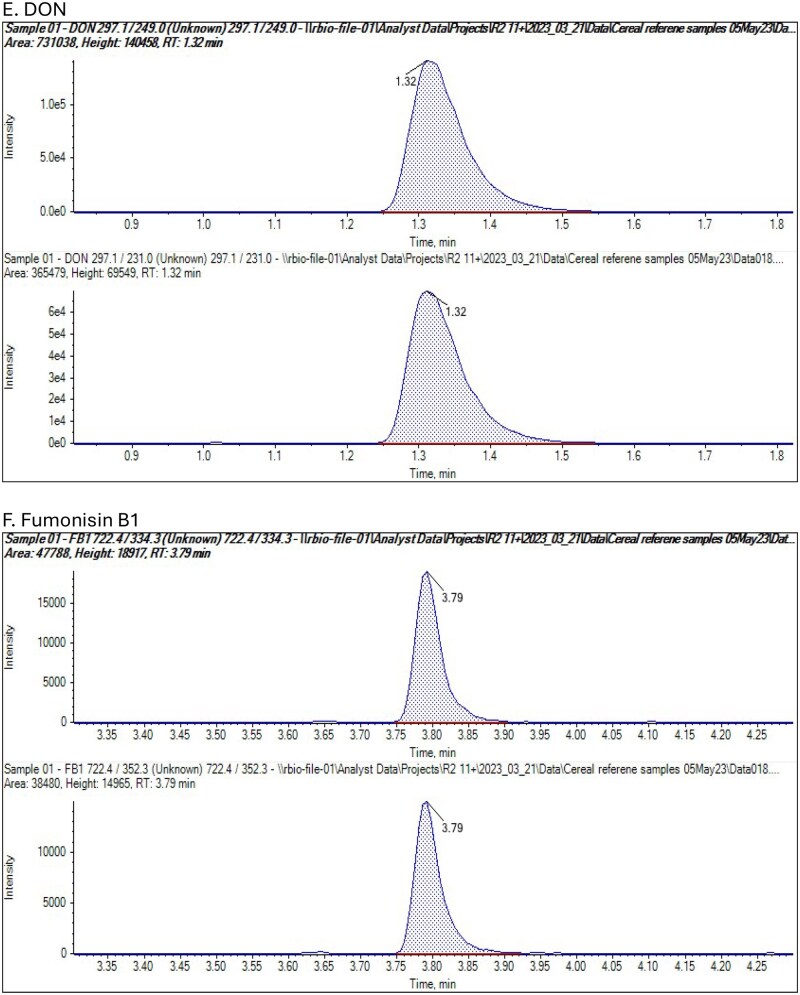

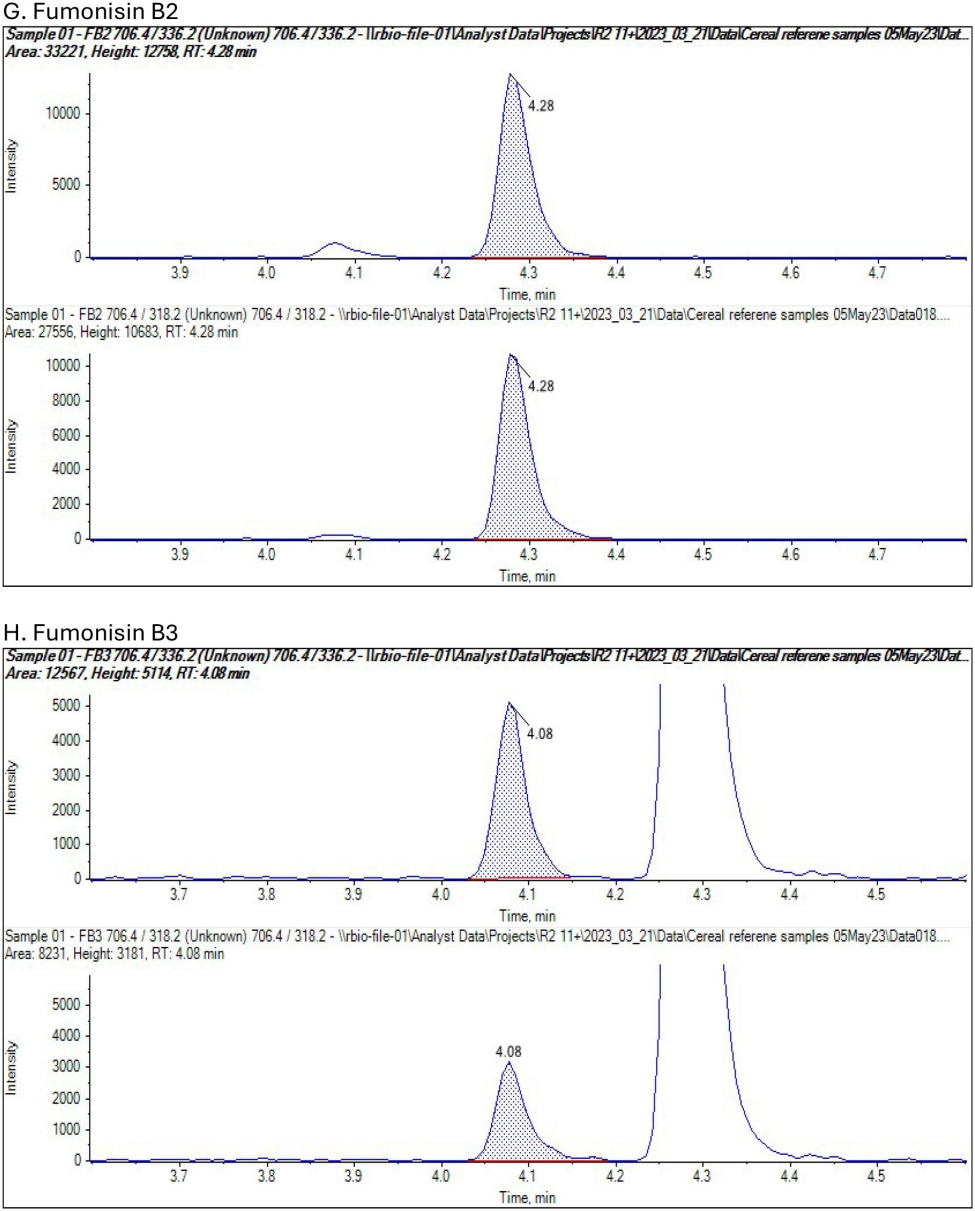

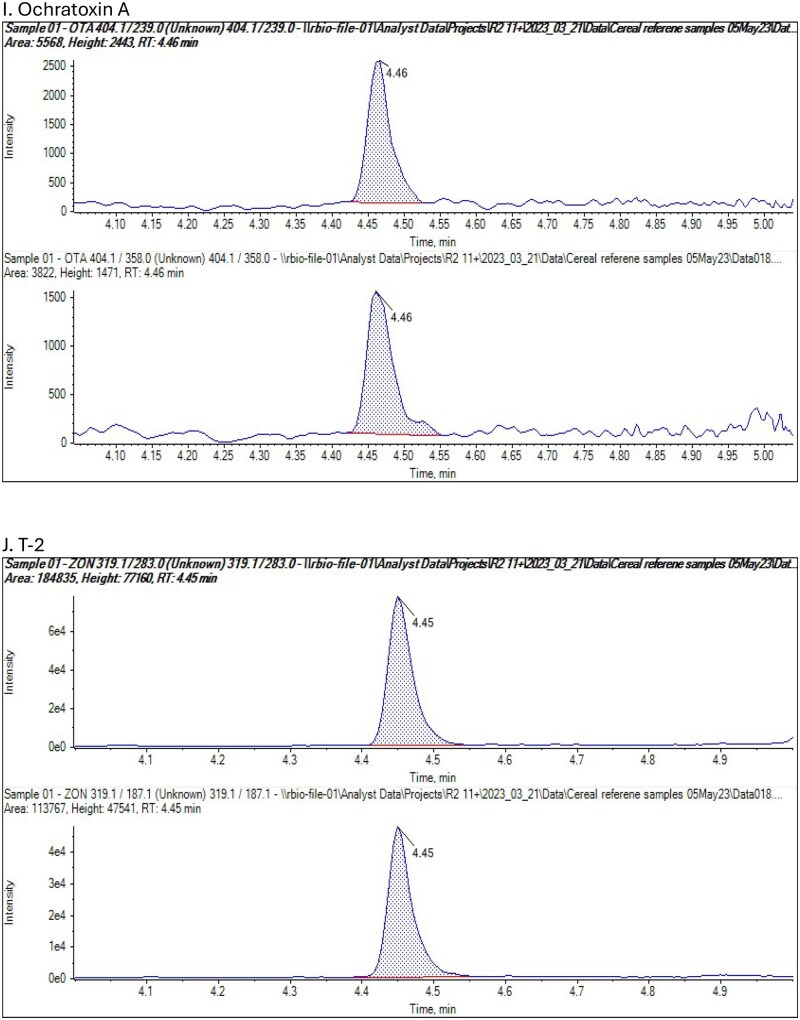

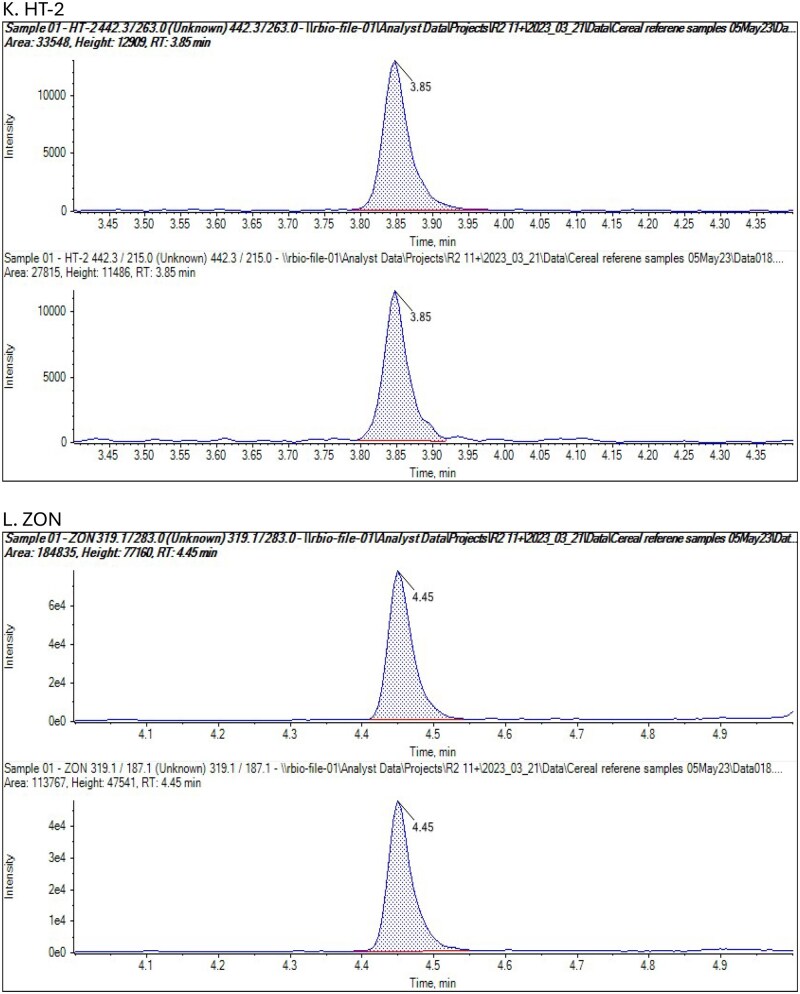
(A–L) Representative chromatograms for FAPAS TCL0406QC corn from the method developer.

**Figure 2.**
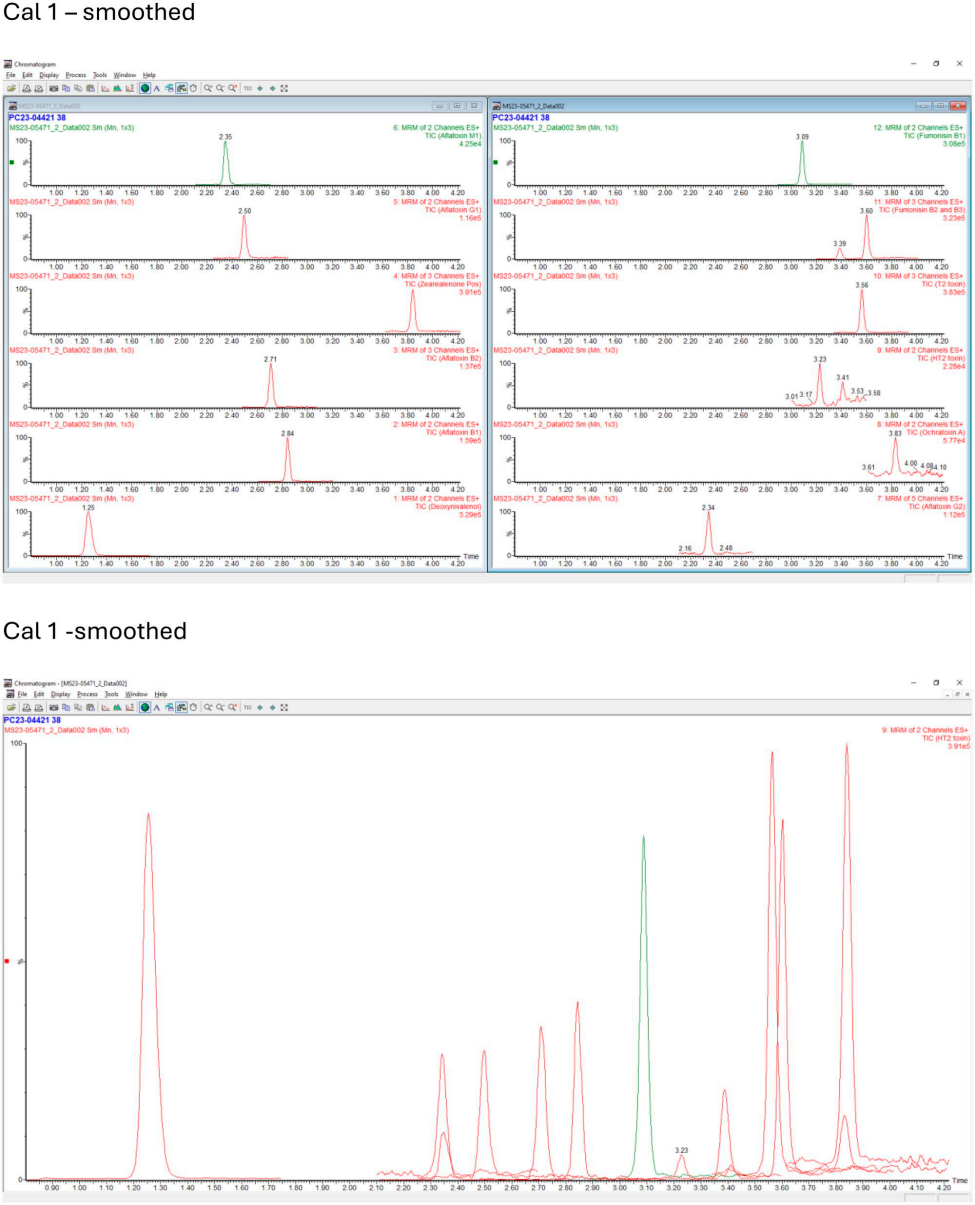
Representative chromatograms from calibration Std1 from the independent laboratory.

**Figure 3.**
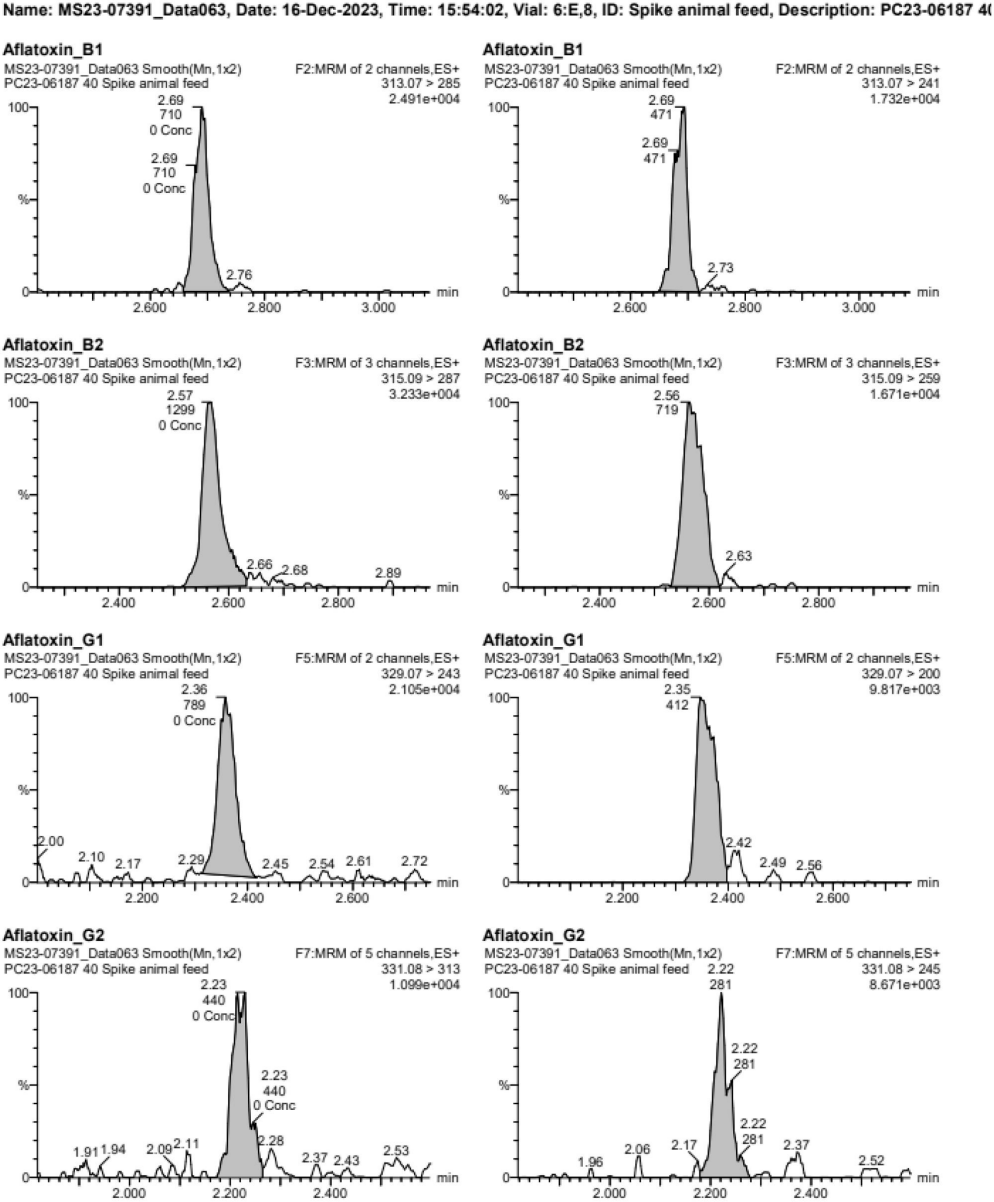

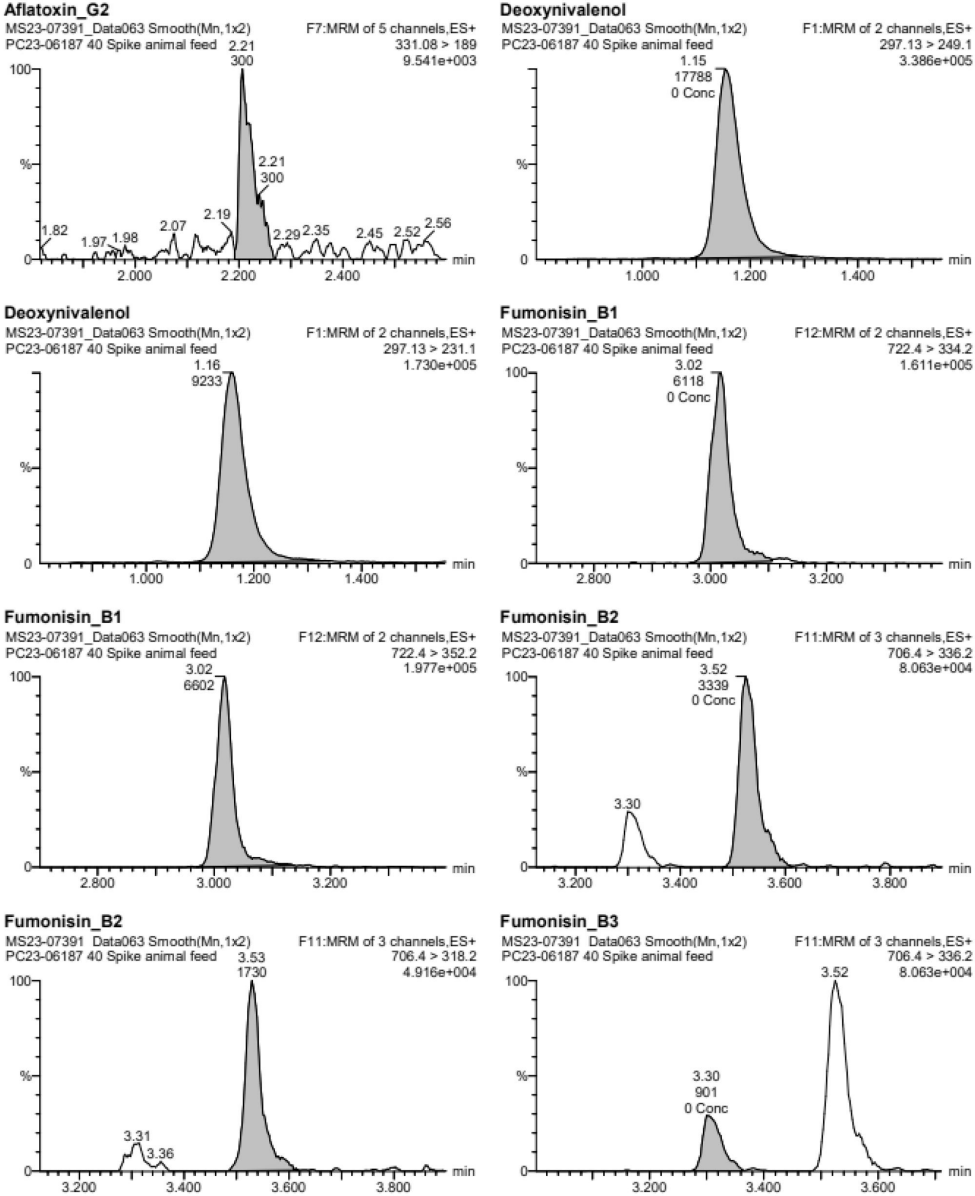

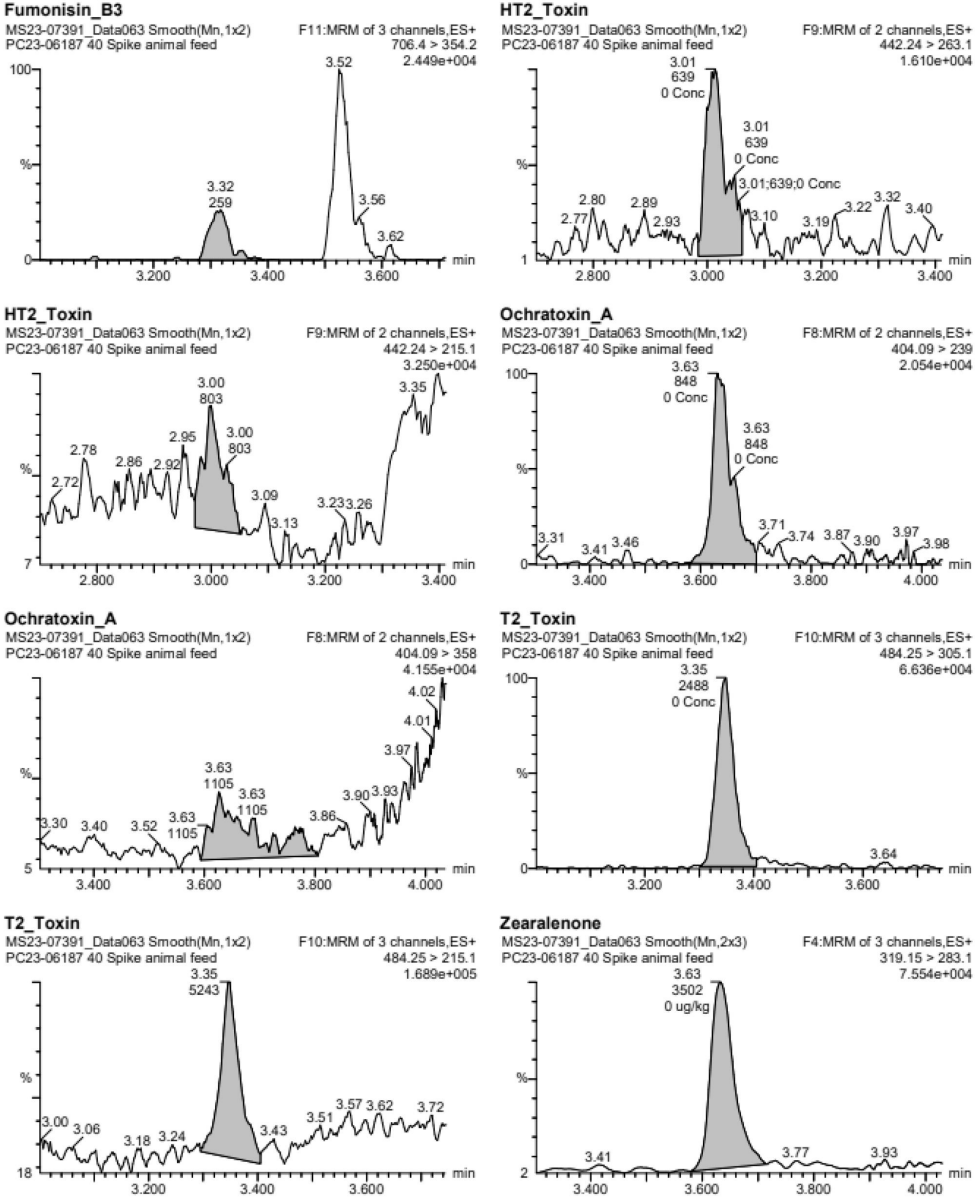

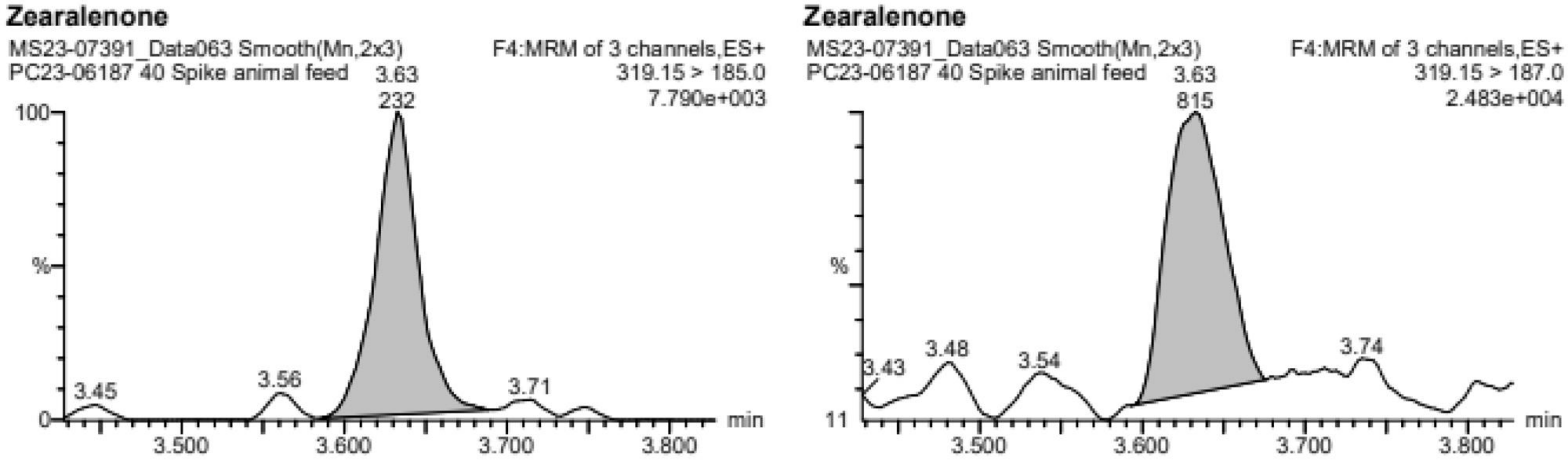
Chromatograms from LOQ spikes in animal feed from the independent laboratory.

The instrumentation used was a Waters Acquity UPLC and Waters Xevo TQ-S instrument (Waters Ltd, Wilmslow, UK). The LC column and conditions were as described in the Analysis section of the method. Retention times were slightly shorter than in the method developer laboratory. Representative chromatograms for all analytes are presented in Figure 1 for the method developer, and Figures 2 and 3 for the independent laboratory.

All samples, standards, or materials stored refrigerated were allowed to reach room temperature before use. Samples were stirred or mixed before test portions were removed in case of settling or segregation and to ensure the test portion was representative. Immunoaffinity columns were at ambient temperature before use.

A combined working solution was prepared by measuring a volume of 10 mL 50% MeOH into a vial and removing 1.25 mL to waste. Appropriate volumes of stock standards were added to prepare the combined working solution at 5.0 ng/mL AFLA, 5.0 µg/mL DON, 1.5 µg/mL FUM, 5 ng/mL OTA, 100 ng/mL T-2, 100 ng/mL HT-2, and 250 ng/mL ZON. Diluted calibration standards in 50% MeOH were prepared fresh on each day of analysis from the combined working stock to yield the concentrations in Supplemental Table 2.

The injection sequence used by the independent laboratory deviated from the method as written. For each batch, the sequence was three injections of Std7; one injection standard diluent; one injection each Std1–Std7; two injections standard diluent; one injection each of up to 15 unknown extracts; one injection Std4; one injection standard diluent; continue unknown extract injections up to 15; two injections standard diluent; one injection each Std1–Std7; and one injection standard diluent.

Data were collected and processed using MassLynx 4.2 CN997 software. Raw data (including peak areas) were transferred to Excel templates to calculate the results in ng/mL in the solution injected. This was converted to µg/kg equivalent values in matrix based on the volume of extract used for sample cleanup.

All data met independent laboratory internal criteria for system suitability.

A triple quad mass spectrometer was used to detect and confirm results. Peak identity was confirmed by comparing the retention time and the ion ratio of the quantification/qualification ions with those of standards injected in the same sequence. The criteria for acceptable ion ratio and retention time were ±30% and ±0.2 min respectively. In all cases, criteria used for assessing data are based on the requirements set out in EURL-MP Guidance Document on performance criteria (17).

## Results and Discussion

### Calibration Study

**Figure 4.**
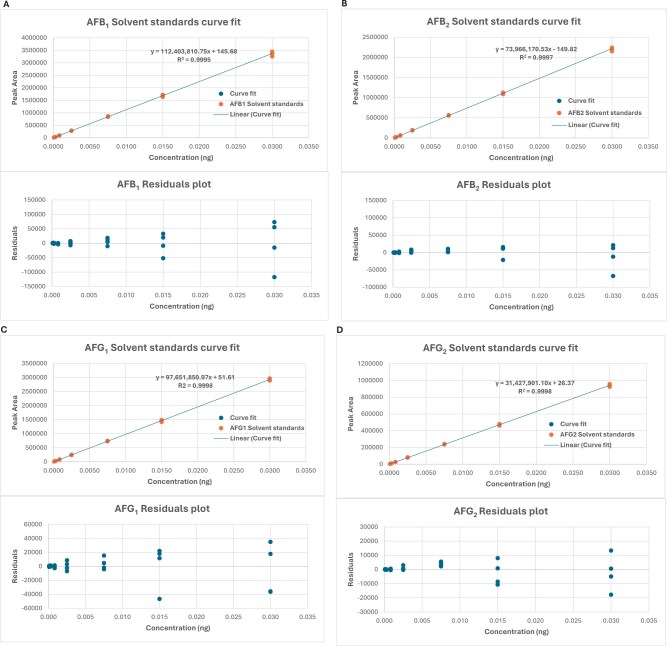

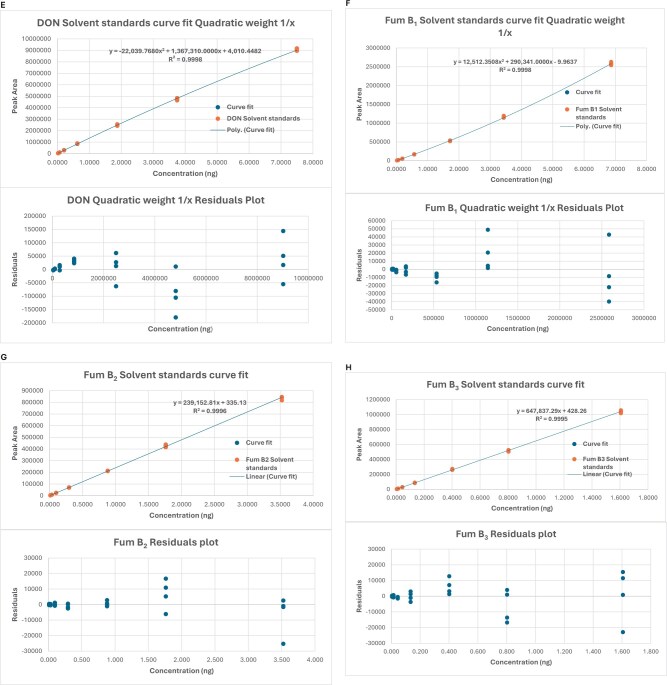

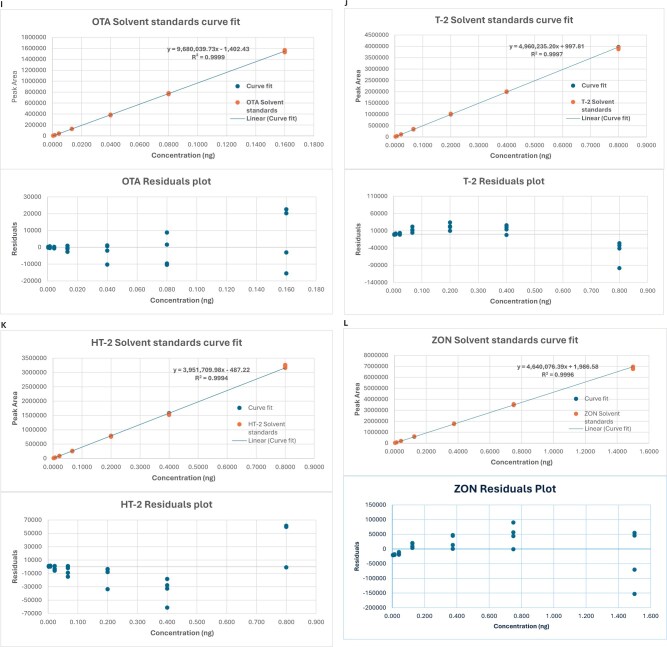
Solvent-based calibration plots and residuals for (A) AFB_1_, (B) AFB_2_, (C) AFG_1_, (D) AFG_2_, (E) DON, (F) FB_1_, (G) FB_2_, (H) FB_3_, (I) OTA, (J) T-2, (K) HT-2, and (L) ZON from the method developer.

The calibration study showed that the relationship between the signal output of the method and the analyte concentration (Figure 4) (as well as potential matrix effects) was acceptable, and without interference from matrix effects. A linear regression analysis was performed, comparing solution standards with matrix-matched standards. A linear fit with weighting of 1/*x* was used for all analytes, except DON and fumonisin B_1_ where a quadratic fit with weighting of 1/*x* was employed. For the purposes of comparing solvent and matrix-matched standards, calibration standards 1 to 5 were used with a linear curve with a weighting of 1/*x*^2^ for DON and fumonisin B_1_. There was no case where a ≥20% matrix effect on a calibration curve was observed (Table 2). Therefore, the solvent-based mixed standard calibration curve was sufficient.

**Table 2.** Matrix effects—solvent versus matrix-matched standards

Toxin	Curve fit, weighting	Percent of solvent curve slope						
		Corn	Wheat	Infant food, non-dairy	Infant food, dairy	Animal feed	Paprika	Chili
Aflatoxin B_1_	Linear, 1/*x*	95.8	97.3	95.9	94.5	94.6	101.0	98.5
Aflatoxin B_2_	Linear, 1/*x*	98.4	98.4	97.2	96.1	97.4	100.4	95.4
Aflatoxin G_1_	Linear, 1/*x*	96.7	99.5	98.3	92.9	96.9	100.2	97.5
Aflatoxin G_2_	Linear, 1/*x*	97.1	99.2	99.7	98.1	97.6	99.5	97.1
Aflatoxin M_1_	Linear, 1/*x*	98.6	100.3	96.8	98.7	ND^a^	ND^a^	ND^a^
DON	Linear, 1/*x*^2^	95.7	99.7	94.2	95.1	96.6	101.1	101.5
FB_1_^b^	Linear, 1/*x*^2^	96.9	98.7	90.1	93.2	98.0	98.2	102.9
FB_2_	Linear, 1/*x*	101.3	99.1	102.1	104.2	97.0	102.1	103.5
FB_3_	Linear, 1/*x*	99.3	98.1	96.4	97.0	98.8	98.4	103.3
OTA	Linear, 1/*x*	99.8	101.9	105.2	106.3	98.6	101.3	100.5
T-2	Linear, 1/*x*	96.2	97.1	96.5	96.1	99.0	104.7	100.5
HT-2	Linear, 1/*x*	96.9	96.4	101.6	97.3	94.7	99.6	100.6
ZON	Linear, 1/*x*	94.8	98.4	98.3	98.8	97.1	118.7	109.6

a ND = Not determined.

b Linear curve with lower five points taken.

### Selectivity

Selectivity determines the ability of the 11^+^Myco MS-PREP method to react to all variants of the analytes and exclude similar compounds or other mycotoxins that could be encountered in the claimed matrixes. There was no evidence of significant positive or negative interferences observed from all non-analyte challenges (Table 3). No positive interferences were observed where only non-analyte was added and the analyte was not present in blank extract. In the case of ZON, which was present at native levels in the matrix used, there were no significant changes to the measured amount (*see*  Table 3). No positive or negative interference was found, when comparing the 11^+^ spike and non-analyte & 11^+^ spike solutions. Therefore, the ability of the 11^+^Myco MS-PREP method to react to all variants of the analytes and exclude similar compounds or other mycotoxins that could be encountered in the claimed matrixes has been demonstrated (refer to Supplemental Figure 2).

**Table 3.** Selectivity results in corn matrix extract

Non-analyte	Analyte	11+ Myco results, µg/kg														
		AFB_1_	AFB_2_	AFG_1_	AFG_2_	AFT^a^	DON	FB_1_	FB_2_	FB_3_	FUM^c^	OTA	T-2	HT-2	T-2 +HT-2	ZON
None	None	ND^b^	ND	ND	ND	ND	ND	ND	ND	ND	ND	ND	ND	ND	ND	5.13
	Std7	1.50	1.55	1.50	1.46	6.01	389.6	329.1	176.5	78.7	584.3	8.0	43.3	40.9	84.1	83.8
Sterigmatocystin, 60 ng/mL	None	ND	ND	ND	ND	ND	ND	ND	ND	ND	ND	ND	ND	ND	ND	5.15
	Std7	1.47	1.53	1.48	1.47	5.96	379.2	325.4	166.5	79.1	570.9	8.1	42.7	40.2	82.8	83.5
Nivalenol, 375 ng/mL	None	ND	ND	ND	ND	ND	ND	ND	ND	ND	ND	ND	ND	ND	ND	5.41
	Std7	1.42	1.56	1.47	1.48	5.93	399.3	319.8	172.3	79.4	571.6	8.2	43.3	37.9	81.2	83.7
Neosolaniol, 800 ng/mL	None	ND	ND	ND	ND	ND	ND	ND	ND	ND	ND	ND	ND	ND	ND	5.12
	Std7	1.41	1.39	1.38	1.51	5.69	393.3	329.5	163.9	76.8	570.2	7.8	40.8	39.3	80.1	80.5
α−Zearalenol, 750 ng/mL	None	ND	ND	ND	ND	ND	ND	ND	ND	ND	ND	ND	ND	ND	ND	5.32
	Std7	1.42	1.47	1.46	1.50	5.84	408.7	331.2	170.3	79.9	581.5	8.0	42.7	40.7	83.4	83.9

a AFT = Total Aflatoxin.

b ND = Not detected.

c FUM = Total fumonisin.

### Matrix Study

The matrix study was conducted on all claimed matrixes to determine bias, recovery, repeatability precision, LOD_est_, and LOQ (Tables 4, 5, 7, and 8). The independent laboratory tested the same corn and animal feed samples as the method developer and except for spiked corn, data show similar results from both laboratories using the method.

**Table 4.** Spiked matrix results

		No spike			Low spike, *n* = 6					Medium spike, *n* = 6					High spike, *n* = 6				
Analyte	n	Mean, µg/kg	Sr, µg/kg	RSDr, %	Mean, µg/kg	Sr, µg/kg	RSDr, %	Rec., %	Bias, µg/kg	Mean, µg/kg	Sr, µg/kg	RSDr, %	Rec., %	Bias, µg/kg	Mean, µg/kg	Sr, µg/kg	RSDr, %	Rec., %	Bias, µg/kg
Corn – method developer																			

AFB_1_	6	ND	ND	ND	1.52	0.060	4.09	121.90	0.270	2.88	0.128	4.45	115.38	0.384	5.91	0.208	3.51	118.12	0.906
AFB_2_	6	ND	ND	ND	1.60	0.060	4.01	128.09	0.350	3.01	0.136	4.51	120.43	0.511	6.13	0.236	3.84	122.66	1.13
AFG_1_	6	ND	ND	ND	1.58	0.050	3.31	126.42	0.330	2.99	0.106	3.56	119.64	0.491	6.08	0.198	3.25	121.70	1.08
AFG_2_	6	ND	ND	ND	1.60	0.080	4.92	128.32	0.350	3.08	0.133	4.33	123.01	0.575	6.09	0.177	2.91	121.89	1.09
AFT	6	ND	ND	ND	6.31	0.228	3.61	126.18	1.31	11.96	0.490	4.10	119.61	1.96	24.22	0.757	3.12	121.09	4.22
DON	6	ND	ND	ND	459.47	13.96	3.04	122.53	84.47	864.67	30.66	3.55	115.29	114.67	1668.42	41.18	2.47	111.23	168.42
FB_1_	6	ND	ND	ND	322.87	18.79	5.82	112.82	36.69	638.94	23.21	3.63	111.63	66.59	1317.60	65.44	4.97	115.10	172.89
FB_2_	6	ND	ND	ND	167.06	5.69	3.41	113.75	20.19	325.15	12.77	3.93	110.69	31.41	664.53	21.41	3.22	113.12	77.06
FB_3_	6	ND	ND	ND	76.34	3.51	4.60	114.03	9.39	149.28	6.66	4.46	111.48	15.37	299.92	7.21	2.40	111.98	32.10
FUM	6	ND	ND	ND	566.27	26.56	4.69	113.25	66.27	1113.38	25.66	2.30	111.34	113.38	2282.04	71.88	3.15	114.10	282.04
OTA	6	ND	ND	ND	1.83	0.081	4.43	121.83	0.33	3.46	0.129	3.73	115.44	0.463	6.98	0.255	3.65	116.31	0.979
T-2	6	ND	ND	ND	6.00	0.371	6.18	120.06	1.00	11.39	0.347	3.05	113.93	1.39	23.26	0.776	3.34	116.31	3.26
HT-2	6	ND	ND	ND	6.14	0.169	2.75	122.80	1.14	11.92	0.151	1.27	119.20	1.92	24.08	0.957	3.97	120.40	4.08
Sum T-2 & HT-2	6	ND	ND	ND	12.14	0.477	3.92	121.43	2.14	23.31	0.438	1.88	116.56	3.31	47.34	1.65	3.49	118.36	7.34
ZON	6	5.57	0.561	9.1	64.88	1.41	2.17	129.76	14.88	121.22	3.85	3.17	121.22	21.22	246.04	6.82	2.77	123.02	46.04

Corn – independent laboratory																			

AFB_1_	10	ND	ND	ND	1.32	0.130	9.66	105.37	0.070	2.72	0.100	3.78	108.98	0.22	5.23	0.33	6.40	104.57	0.23
AFB_2_	10	ND	ND	ND	1.41	0.110	7.54	112.44	0.160	2.89	0.120	4.16	115.52	0.39	5.49	0.27	4.98	109.85	0.49
AFG_1_	10	ND	ND	ND	1.30	0.110	8.64	103.73	0.050	2.64	0.100	3.93	105.43	0.14	5.17	0.31	5.96	103.33	0.17
AFG_2_	10	ND	ND	ND	1.37	0.080	5.53	109.58	0.120	2.78	0.160	5.83	111.09	0.28	5.41	0.27	4.93	108.16	0.41
AFT	10	ND	ND	ND	5.39	0.410	7.61	107.78	0.390	11.03	0.450	4.12	110.26	1.03	21.30	1.16	5.46	106.48	1.30
DON	10	ND	ND	ND	433.36	31.46	7.26	115.56	58.36	794.20	92.61	11.66	105.89	58.36	1604.40	247.20	15.40	106.95	104.35
FB_1_	10	ND	ND	ND	251.40	13.61	5.41	87.85	–34.77	534.06	44.92	8.41	93.30	–38.34	1140.70	53.56	4.70	99.64	104.35
FB_2_	10	ND	ND	ND	157.90	9.59	6.07	107.52	–10.70	321.93	28.21	8.76	109.59	28.17	625.64	37.17	5.94	106.49	–4.09
FB_3_	10	ND	ND	ND	65.94	3.54	5.36	98.48	–1.02	136.38	13.55	9.93	101.84	2.46	274.10	21.73	7.93	102.34	38.12
FUM	10	ND	ND	ND	475.30	26.53	5.58	95.05	–24.71	992.40	86.40	8.71	99.23	–7.63	2040.50	108.34	5.31	102.01	40.45
OTA	10	ND	ND	ND	1.45	0.050	3.66	96.85	–0.050	2.87	0.160	5.75	95.54	–0.13	5.62	0.40	7.10	93.61	2.49
T-2	10	ND	ND	ND	4.64	0.440	9.45	92.78	–0.360	9.94	0.450	4.51	99.39	–0.06	20.13	1.12	5.56	100.67	–0.38
HT-2	10	ND	ND	ND	5.60	0.530	9.47	112.00	0.600	11.24	0.480	4.23	112.40	1.24	22.49	1.32	5.89	112.43	6.26
Sum T-2 & HT-2	10	ND	ND	ND	10.24	0.860	8.45	102.39	0.240	21.18	0.870	4.10	105.89	1.18	42.62	2.36	5.53	106.55	2.62
ZON	10	3.67	0.03	0.91	61.58	5.41	8.79	113.80	11.65	114.95	7.43	6.47	110.35	15.09	216.71	22.31	10.29	106.12	0.13

Wheat – method developer																			

AFB_1_	6	ND	ND	ND	1.44	0.058	4.07	114.86	0.186	2.75	0.075	2.74	110.11	0.253	5.45	0.347	6.37	108.91	0.446
AFB_2_	6	ND	ND	ND	1.49	0.061	4.10	118.85	0.236	2.91	0.116	4.01	116.28	0.407	5.71	0.244	4.28	114.24	0.712
AFG_1_	6	ND	ND	ND	1.46	0.029	2.02	116.42	0.205	2.80	0.118	4.20	112.00	0.300	5.55	0.280	5.04	110.98	0.549
AFG_2_	6	ND	ND	ND	1.55	0.024	1.58	123.62	0.295	2.85	0.097	3.39	114.13	0.353	5.74	0.263	4.58	114.71	0.736
AFT	6	ND	ND	ND	5.92	0.144	2.44	118.44	0.922	11.31	0.352	3.11	113.13	1.313	22.44	1.045	4.66	112.21	2.44
DON	6	9.78	0.597	6.11	433.46	14.54	3.36	115.59	58.46	808.64	25.99	3.21	107.82	58.64	1561.33	37.88	2.43	104.09	61.33
FB_1_	6	ND	ND	ND	263.50	9.55	3.63	92.07	–22.68	515.32	9.69	1.88	90.04	–57.03	1026.86	40.05	3.90	89.70	–117.85
FB_2_	6	ND	ND	ND	137.73	5.14	3.73	93.78	–9.14	268.88	9.38	3.49	91.54	–24.86	535.17	26.50	4.95	91.10	–52.30
FB_3_	6	ND	ND	ND	62.55	2.25	3.59	93.42	–4.40	122.74	4.38	3.57	91.66	–11.17	243.34	9.52	3.91	90.86	–24.48
FUM	6	ND	ND	ND	463.78	15.78	3.40	92.76	–36.22	906.94	19.04	2.10	90.69	–93.06	1805.38	70.60	3.91	90.27	–194.62
OTA	6	ND	ND	ND	1.93	0.088	4.58	128.68	0.430	3.50	0.153	4.36	116.75	0.503	6.95	0.276	3.96	115.81	0.949
T-2	6	ND	ND	ND	5.60	0.250	4.46	111.98	0.599	10.88	0.528	4.85	108.80	0.880	21.24	0.993	4.68	106.22	1.24
HT-2^a^	6	0.205	0.233	113.5	5.92	0.487	8.22	118.42	0.921	11.09	0.191	1.72	110.88	1.09	21.96	0.963	4.38	109.82	1.96
Sum T-2 & HT-2	6	0.205	0.233	113.5	11.52	0.642	5.57	115.20	1.52	21.97	0.591	2.69	109.84	1.97	43.21	1.60	3.70	108.02	3.21
ZON	6	ND	ND	ND	50.84	1.57	3.09	101.67	0.836	96.97	1.60	1.65	96.97	–3.03	192.86	7.11	3.69	96.43	–7.14

Cereal-based infant food with dairy – method developer																			

AFB1^b^	6	0.00872	0.0044	49.9	0.0582	0.0035	6.01	116.45	0.0082	0.108	0.004	3.90	108.21	0.0082	0.199	0.006	3.08	99.45	–0.0011
AFB2	6	ND	ND	ND	0.0542	0.0022	4.05	108.49	0.0042	0.107	0.009	8.27	106.98	0.0070	0.200	0.010	5.10	99.81	–0.0004
AFG_1_	6	ND	ND	ND	0.0511	0.0018	3.52	102.15	0.0011	0.103	0.005	5.32	102.84	0.0028	0.199	0.010	5.16	99.40	–0.0012
AFG_2_	6	ND	ND	ND	0.0495	0.0034	6.95	98.90	–0.0005	0.103	0.007	6.82	103.44	0.0034	0.204	0.006	2.81	101.98	0.0040
AFT	6	0.00872	0.0044	49.9	0.213	0.0043	2.01	106.50	0.013	0.421	0.019	4.50	105.37	0.021	0.801	0.020	2.54	100.16	0.0013
AFM1	6	ND	ND	ND	0.0529	0.0040	7.61	105.71	0.0029	0.0987	0.005	4.92	98.67	–0.0013	0.200	0.006	2.98	99.82	–0.0004
DON	6	6.78	0.32	4.65	98.35	1.91	1.95	98.35	–1.65	190.6	2.82	1.48	95.30	–9.39	380.0	9.35	2.46	95.00	–20.0
FB_1_	6	ND	ND	ND	56.96	1.39	2.44	99.52	–0.277	112.7	2.17	1.93	98.42	–1.80	228.0	1.05	0.46	103.08	6.82
FB_2_	6	ND	ND	ND	28.68	0.48	1.66	97.65	–0.690	58.7	2.73	4.65	99.95	–0.032	117.2	2.82	2.41	99.79	–0.25
FB_3_	6	ND	ND	ND	11.37	0.35	3.11	84.92	–2.019	22.37	0.55	2.44	83.54	–4.41	45.83	1.37	2.98	85.57	–7.73
FUM	6	ND	ND	ND	97.01	1.35	1.39	97.01	–2.986	193.75	4.18	2.16	96.87	–6.25	391.06	4.47	1.14	97.77	–8.94
OTA^b^	6	0.115	0.010	9.03	0.345	0.014	3.96	138.02	0.095	0.632	0.020	3.10	126.40	0.132	1.14	0.024	2.13	113.67	0.137
T-2	6	ND	ND	ND	2.59	0.108	4.17	103.76	0.094	5.33	0.190	3.57	106.52	0.326	10.64	0.272	2.56	106.39	0.639
HT-2^b^	6	0.136	0.106	78.3	2.85	0.140	4.92	113.95	0.349	5.35	0.287	5.38	106.91	0.345	10.89	0.208	1.90	108.95	0.895
Sum T-2 & HT-2	6	0.136	0.106	78.3	5.44	0.223	4.09	108.85	0.443	10.67	0.446	4.18	106.71	0.671	21.53	0.379	1.76	107.67	1.534
ZON	6	2.75	0.171	6.24	9.46	0.131	1.39	94.64	–0.536	19.11	0.598	3.13	95.54	–0.892	38.51	0.642	1.67	96.27	–1.494

Cereal-based infant food non-dairy – method developer																			

AFB_1_	6	ND	ND	ND	0.0476	0.001	3.14	95.27	–0.0024	0.094	0.0064	6.83	93.79	–0.0062	0.205	0.0052	2.52	102.58	0.0052
AFB_2_	6	ND	ND	ND	0.0570	0.004	6.45	113.95	0.0070	0.102	0.0079	7.71	102.14	0.0021	0.220	0.0052	2.35	109.89	0.020
AFG_1_	6	ND	ND	ND	0.0500	0.002	4.23	100.03	0.0000	0.093	0.0045	4.79	93.05	–0.0070	0.208	0.0052	2.52	104.02	0.0080
AFG_2_	6	ND	ND	ND	0.0505	0.005	9.21	100.98	0.0005	0.097	0.0054	5.60	96.64	–0.0034	0.213	0.0076	3.56	106.52	0.013
AFT	6	ND	ND	ND	0.205	0.010	4.80	102.56	0.0051	0.386	0.020	5.08	96.40	–0.014	0.846	0.013	1.56	105.76	0.046
DON^c^	6	6.45	0.370	0.580	102.6	3.21	3.13	102.57	2.57	190.6	6.84	3.59	95.28	–9.44	408.9	10.77	2.63	102.23	8.93
FB_1_^c^	6	3.26	0.720	22.1	51.61	0.82	1.59	90.17	–5.63	99.1	4.30	4.34	86.59	–15.35	220.4	5.10	2.32	99.63	–0.807
FB_2_	6	ND	ND	ND	28.10	1.02	3.61	95.69	–1.27	52.6	1.80	3.41	89.60	–6.11	119.0	3.02	2.54	101.27	1.49
FB_3_^c^	6	0.562	0.123	22.0	13.17	0.51	3.90	98.39	–0.216	23.76	0.64	2.70	88.71	–3.02	53.47	1.81	3.38	99.84	–0.087
FUM	6	3.82	0.640	16.9	92.89	1.97	2.12	92.89	–7.11	179.6	6.34	3.53	89.78	–20.44	392.8	6.79	1.73	98.20	–7.18
OT^c^	6	0.0392	0.021	52.7	0.318	0.008	2.60	127.32	0.068	0.559	0.022	3.86	111.77	0.059	1.17	0.035	2.97	117.09	0.171
T-2	6	ND	ND	ND	2.45	0.134	5.48	97.89	–0.053	4.63	0.097	2.09	92.50	–0.375	10.42	0.461	4.43	104.19	0.419
HT-2^c^	6	0.373	0.042	11.3	2.73	0.039	1.41	109.24	0.231	4.82	0.119	2.47	96.44	–0.178	10.45	0.230	2.20	104.54	0.454
Sum T-2 & HT-2	6	0.373	0.042	11.3	5.18	0.134	2.58	103.56	0.178	9.45	0.208	2.21	94.47	–0.553	20.87	0.593	2.84	104.37	0.873
ZON	6	ND	ND	ND	8.72	0.141	1.62	87.18	–1.28	16.36	0.256	1.57	81.78	–3.64	36.52	0.774	2.12	91.31	–3.48

Animal feed – method developer																			

AFB_1_	6	ND	ND	ND	2.65	0.114	4.30	105.86	0.147	7.86	0.148	1.89	104.78	0.359	31.44	1.26	4.02	104.80	1.44
AFB_2_	6	ND	ND	ND	2.74	0.128	4.68	109.71	0.243	8.15	0.295	3.62	108.63	0.647	33.13	1.26	3.81	110.45	3.13
AFG_1_	6	ND	ND	ND	2.73	0.127	4.67	109.11	0.228	7.91	0.305	3.85	105.42	0.406	32.76	1.01	3.09	109.21	2.76
AFG_2_	6	ND	ND	ND	2.73	0.119	4.36	109.35	0.234	8.25	0.205	2.48	109.99	0.749	33.08	1.11	3.37	110.25	3.08
AFT	6	ND	ND	ND	10.85	0.414	3.82	108.51	0.851	32.16	0.771	2.40	107.21	2.162	130.41	4.08	3.13	108.68	10.41
DON^d^	6	79.76	8.64	10.84	502.35	17.47	3.48	111.63	52.35	1460.48	29.60	2.03	108.18	110.48	5710.67	193.33	3.39	105.75	310.67
FB_1_^d^	6	45.43	12.07	26.58	542.63	23.58	4.35	94.81	–29.72	1669.42	45.74	2.74	97.23	–47.64	6763.88	130.47	1.93	98.48	–104.37
FB_2_	6	ND	ND	ND	282.30	8.98	3.18	96.11	–11.44	846.88	20.93	2.47	96.52	–30.54	3416.54	120.78	3.54	96.93	–108.30
FB_3_	6	ND	ND	ND	131.89	9.35	7.09	98.49	–2.02	401.12	7.80	1.95	99.85	–0.596	1582.22	51.42	3.25	98.46	–24.69
FUM	6	45.43	12.07	26.58	956.81	33.06	3.45	95.68	–43.19	2917.43	38.45	1.32	97.25	–82.57	11762.64	266.04	2.26	98.02	–237.36
OTA	6	ND	ND	ND	13.96	0.622	4.45	111.70	1.46	40.30	1.12	2.79	107.46	2.80	164.30	7.15	4.35	109.53	14.30
T-2	6	ND	ND	ND	69.39	1.81	2.61	111.03	6.89	202.55	5.87	2.90	108.03	15.05	837.02	18.93	2.26	111.60	87.02
HT-2	6	ND	ND	ND	68.27	2.78	4.07	109.24	5.77	200.06	6.20	3.10	106.70	12.56	790.46	27.36	3.46	105.39	40.46
Sum T-2 & HT-2	6	ND	ND	ND	137.67	2.35	1.71	110.13	12.67	402.62	7.84	1.95	107.36	27.62	1627.47	37.75	2.32	108.50	127.47
ZON^d^	6	1.17	1.84	157.02	52.41	2.02	3.85	104.82	2.41	146.54	4.55	3.11	97.69	–3.46	581.10	17.29	2.98	96.85	–18.90

Animal feed – independent laboratory																			

AFB_1_	10	ND	ND	ND	1.92	0.630	32.98	76.56	–0.590	6.74	0.630	9.35	89.80	–0.770	27.22	1.55	5.69	90.69	–2.79
AFB_2_	10	ND	ND	ND	1.95	0.650	33.18	77.93	–0.550	6.96	0.650	9.27	92.80	–0.540	27.76	1.61	5.79	92.49	–2.25
AFG_1_	10	ND	ND	ND	2.05	0.610	29.58	81.79	–0.460	7.11	0.560	7.87	94.74	–0.400	28.18	1.19	4.21	93.88	–1.84
AFG_2_	10	ND	ND	ND	2.08	0.610	29.38	82.98	–0.430	7.22	0.560	7.76	96.24	–0.280	28.77	1.27	4.42	95.88	–1.24
AFT	10	ND	ND	ND	7.99	2.49	31.20	79.82	–2.02	28.03	2.39	8.53	93.39	–1.98	111.92	5.55	4.96	93.23	–8.12
DON^e^	10	76.62	13.71	17.90	410.26	266.78	65.03	79.46	–39.75	1429.41	459.37	32.14	101.98	79.41	4592.30	1070.60	23.31	84.07	–807.30
FB_1_^e^	10	40.93	11.79	28.80	484.86	23.33	4.81	77.99	–87.52	1381.30	34.16	2.47	78.20	–335.89	6356.30	284.74	4.48	91.99	–511.57
FB_2_^e^	10	7.46	0.96	12.94	253.40	15.12	5.97	85.85	–40.35	748.66	25.05	3.35	84.82	–132.61	3050.60	106.28	3.48	86.51	–474.13
FB_3_^e^	10	3.68	1.00	27.33	109.45	4.78	4.37	81.08	–24.47	326.07	15.04	4.61	80.94	–75.69	1385.80	30.74	2.22	86.19	–221.08
FUM	10	52.07	12.93	24.84	847.71	22.99	2.71	80.71	–152.35	2456.00	66.71	2.72	80.51	–544.19	10792.70	375.54	3.48	89.60	–1207.34
OTA	10	ND	ND	ND	11.06	0.920	8.36	88.49	–1.44	33.40	0.87	2.61	89.08	–4.09	133.93	11.97	8.94	89.32	–16.01
T-2^e^	10	0.290	0.150	50.37	46.96	21.96	46.77	75.12	–15.55	170.91	30.83	18.04	91.14	–16.61	710.16	69.02	9.72	94.69	–39.84
HT-2^e^	10	2.51	0.57	22.66	45.54	22.18	48.71	72.16	–16.97	174.17	30.41	17.46	92.65	–13.35	690.98	78.70	11.39	92.07	–59.02
Sum T-2 & HT-2	10	2.80	0.62	22.05	92.50	44.14	47.72	73.65	–32.50	345.08	60.18	17.44	91.90	–29.95	1401.10	147.28	10.51	93.38	–98.86
ZON^e^	10	0.710	0.780	109.83	35.09	14.10	40.19	70.23	–14.87	124.64	19.01	15.25	83.15	–25.26	477.43	51.46	10.78	79.64	–122.08

Paprika – method developer																			

AFB_1_^f^	6	0.024	0.013	54.00	1.19	0.065	5.47	94.85	–0.064	2.22	0.200	9.03	88.63	–0.284	4.38	0.349	7.97	87.50	–0.625
AFB_2_	6	ND	ND	ND	1.27	0.054	4.29	101.28	0.016	2.42	0.199	8.25	96.75	–0.081	4.81	0.415	8.63	96.14	–0.193
AFG_1_	6	ND	ND	ND	1.26	0.070	5.55	100.79	0.010	2.42	0.239	9.86	96.89	–0.078	4.70	0.354	7.54	93.94	–0.303
AFG_2_	6	ND	ND	ND	1.29	0.060	4.64	103.22	0.040	2.54	0.258	10.16	101.56	0.039	4.93	0.273	5.52	98.70	–0.065
AFT	6	0.024	0.013	54.00	5.00	0.245	4.90	100.04	0.002	9.60	0.879	9.16	95.96	–0.404	18.81	1.37	7.30	94.07	–1.19
DON	6	ND	ND	ND	54.63	0.992	1.82	109.26	4.63	114.96	4.92	4.28	114.96	14.96	227.37	9.28	4.08	113.69	27.37
FB_1_	6	ND	ND	ND	52.56	3.97	7.56	95.06	–2.73	108.28	6.86	6.34	97.92	–2.30	210.77	8.74	4.15	92.06	–18.17
FB_2_	6	ND	ND	ND	26.44	2.48	9.38	90.01	–2.93	55.65	1.87	3.36	94.72	–3.10	107.23	2.82	2.63	91.26	–10.26
FB_3_	6	ND	ND	ND	13.22	0.715	5.41	98.75	–0.17	26.22	2.11	8.06	97.91	–0.559	51.52	2.19	4.25	96.19	–2.04
FUM	6	ND	ND	ND	92.22	4.98	5.40	92.22	–7.78	190.14	8.45	4.45	95.07	–9.86	369.51	9.72	2.63	92.38	–30.49
OTA	6	2.35	0.732	31.10	11.76	2.87	24.37	117.60	1.76	18.60	2.05	11.00	92.99	–1.40	36.27	3.29	9.07	90.68	–3.73
T-2	6	ND	ND	ND	5.17	0.276	5.33	103.39	0.17	11.01	1.27	11.53	110.13	1.01	21.56	1.94	9.02	107.81	1.56
HT-2	6	ND	ND	ND	6.47	0.200	3.10	129.30	1.47	11.35	0.69	6.08	113.55	1.35	21.91	0.903	4.12	109.53	1.91
Sum T-2 & HT-2	6	ND	ND	ND	11.63	0.456	3.92	116.34	1.63	22.99	1.22	5.32	114.93	2.99	43.47	2.76	6.35	108.67	3.47
ZON^f^	6	0.532	0.366	68.68	22.55	1.60	7.10	90.19	–2.45	41.50	5.35	12.88	83.00	–8.50	81.00	6.45	7.96	81.00	–19.00

Chili powder – method developer																			

AFB_1_^g^	6	0.32	0.02	5.69	1.18	0.07	6.23	94.79	–0.07	2.41	0.09	3.72	96.23	–0.09	5.15	0.17	3.39	102.99	0.15
AFB_2_	6	ND	ND	ND	1.15	0.04	3.22	92.32	–0.10	2.40	0.06	2.42	96.12	–0.10	4.90	0.14	2.94	97.99	–0.10
AFG_1_	6	ND	ND	ND	1.17	0.03	2.53	93.99	–0.08	2.43	0.07	2.98	97.38	–0.07	4.98	0.18	3.64	99.64	–0.02
AFG_2_	6	ND	ND	ND	1.14	0.04	3.85	90.86	–0.11	2.42	0.10	4.23	96.67	–0.08	4.89	0.11	2.32	97.72	–0.11
AFT	6	ND	ND	ND	4.65	0.12	2.60	92.99	–0.35	9.66	0.27	2.82	96.60	–0.34	19.60	0.48	2.44	98.01	–0.40
DON	6	ND	ND	ND	46.13	1.96	4.25	92.26	–3.87	101.34	3.71	3.66	101.34	1.34	206.20	5.24	2.54	103.10	6.20
FB_1_	6	ND	ND	ND	55.98	3.59	6.42	93.78	–3.71	112.93	6.65	5.88	94.60	–6.44	232.84	7.47	3.21	80.92	–54.90
FB_2_	6	ND	ND	ND	27.60	2.35	8.51	95.55	–1.29	53.81	4.08	7.57	93.15	–3.96	109.90	8.82	8.03	95.11	–5.65
FB_3_	6	ND	ND	ND	11.02	0.59	5.36	82.31	–2.37	22.01	1.51	6.86	96.34	–0.84	44.91	0.98	2.19	98.26	–0.80
FUM	6	ND	ND	ND	94.60	5.01	5.30	94.60	–5.40	188.76	12.01	6.36	94.38	–11.24	387.66	10.33	2.66	96.91	–12.34
OTA^g^	6	4.90	0.85	17.37	8.88	1.05	11.86	88.83	–1.12	19.15	2.69	14.07	95.73	–0.85	37.32	0.80	2.15	93.31	–2.68
T-2	6	ND	ND	ND	5.56	0.43	7.80	111.18	0.56	10.55	0.80	7.60	105.55	0.55	21.50	1.13	5.24	107.51	1.50
HT-2^h^	6	0.20	0.23	113.55	5.73	0.30	5.26	114.54	0.73	10.85	0.61	5.65	108.47	0.85	21.20	0.59	2.80	105.99	1.20
Sum T-2 & HT-2	6	0.20	0.23	113.55	11.29	0.46	4.11	112.86	1.29	21.09	1.16	5.52	105.45	1.09	42.70	1.35	3.16	106.75	2.70
ZON^i^	6	5.89	0.77	13.07	18.22	2.64	14.49	72.89	–6.78	39.96	4.52	11.32	79.92	–10.04	80.49	3.51	4.36	80.49	–19.51

a HT-2 detected in blank test portions at levels below lowest calibration standard used.

b Aflatoxin B1, OTA, and HT-2 detected in blank test portions at levels less than lowest calibration standard.

c DON, Fumonisin B1, Fumonisin B3, OTA, and HT-2 values less than lowest calibration standard.

d DON, Fumonisin B1, and ZON detected in blank sample. ZON was in 2 of the 6 test portions.

e DON, Fumonisin B1, Fumonisin B2, Fumonisin B3, T-2, HT-2, and ZON detected in blank sample.

f Aflatoxin B1 and ZON detected in 5 of 6 test portions at levels under lowest calibration standard.

g AFB1 and OTA detected in blank sample.

h HT-2 detected in four of six test portions at levels below calibration standard 1.

i ZON values just below calibration standard 1.

**Table 5.** Reference and QC material results (*n* = 6)

Parameter	AFB_1_	AFB_2_	AFG_1_	AFG_2_	AFLA	DON	FB_1_	FB_2_	FB_3_	FUM (1 + 2 + 3)^a^		FUM (1 + 2)^b^	OTA	T-2	HT-2	T-2+HT-2	ZON
Corn—FAPAS TCL0406QC—method developer																	

Mean, µg/kg	0.483	0.436	0.504	0.446	1.87	335.72	111.49	111.46	13.74^c^	236.69		222.95	0.496	24.43	13.63	39.07	31.90
S_r_, µg/kg	0.018	0.014	0.020	0.016	0.047	4.84	6.08	6.61	0.93	10.78		11.19	10.78	1.36	2.57	3.75	0.86
RSD_r_, %	3.68	3.19	3.91	3.51	2.52	1.44	5.46	5.93	6.75	4.73		4.83	6.75	5.55	18.85	9.84	2.69
Rec., %	92.88	106.27	100.85	110.05	99.93	112.66	83.83	83.81	NA^d^	NA		88.12	95.34	120.35	84.17	104	107.05
Bias, µg/kg	−0.037	0.026	0.004	0.041	−0.001	37.72	−21.51	−21.54	NA	NA		−16.31	−0.02	4.13	−2.57	1.47	2.10

Corn—FAPAS TCL0406QC—independent laboratory																	

Mean, µg/kg	0.570	0.470	0.510	0.430	1.98	328.70	117.24	121.37	14.66^c^	253.27		238.61	0.52	23.4	14.66	38.06	29.61
S_r_, µg/kg	0.024	0.015	0.023	0.018	0.050	30.09	9.15	19.19	12.67	11.96		11.17	0.010	0.760	0.730	1.32	2.05
RSD_r_, %	4.23	3.13	4.41	4.27	2.54	9.15	4.96	4.74	7.87	4.72		4.68	2.54	3.23	4.97	3.46	6.92
Rec., %	109.1	115.16	101.31	106.49	105.74	110.3	88.15	91.25	NA	NA		94.30	100.07	115.29	100.11	103.99	99.36
Bias, µg/kg	0.05	0.06	0.01	0.03	0.11	30.7	−15.76	−11.63	NA	NA		14.40	−0.1	3.1	0.27	1.46	−0.19

Wheat—FAPAS T22187QC—method developer																	

Mean, µg/kg	2.72	1.51	1.76	0.985	6.97	833	ND^e^	ND	ND	ND		ND	ND	34.93	21.45	56.38	67.25
S_r_, µg/kg	0.055	0.080	0.053	0.033	0.20	16.2	ND	ND	ND	ND		ND	ND	1.67	1.00	2.56	1.74
RSD_r_, %	2.03	5.30	3.01	3.31	2.85	1.94	ND	ND	ND	ND		ND	ND	4.78	4.78	4.55	2.59
Rec., %	104.45	98.62	103.65	109.45	104.21	111.83	ND	ND	ND	ND		ND	ND	107.80	96.20	104.41	90.38
Bias, µg/kg	0.12	−0.021	0.062	0.085	0.28	88.11	ND	ND	ND	ND		ND	ND	2.53	−0.85	2.38	−7.15

Wheat—Trilogy TE-QC-MYC-20-1WHE—method developer																	

Mean, µg/kg	11.90	0.829^c^	0.095^c^	ND	11.90	1833.43	560.73	169.05	87.89	817.66		729.77	25.26	435.62	253.23	688.89^c^	407.58
S_r_, µg/kg	4.54	0.291	0.030	ND	4.54	48.61	60.27	13.22	9.84	82.26		73.16	5.82	49.19	12.98	55.52	11.54
RSD_r_, %	38.19	35.08	31.56	ND	38.19	2.65	10.75	7.82	11.19	10.06		10.02	23.03	11.29	5.13	8.06	2.83
Rec., %	112.23	ND	ND	ND	112.23	141.03	112.15	84.52	87.89	102.21		NA	114.81	97.69	128.22	ND	105.62
Bias, µg/kg	1.30	ND	ND	ND	1.30	533.43	60.73	−30.95	−12.11	17.66		NA	3.26	−10.28	55.73	ND	21.68

Cereal-based infant food (non-dairy)—FAPAS T04412QC—method developer																	

Mean, µg/kg	0.100	0.102	ND	ND	0.202	166.44	ND	ND	ND	ND		ND	0.471	ND	ND	ND	ND
S_r_, µg/kg	0.0038	0.0027	ND	ND	0.0049	4.00	ND	ND	ND	ND		ND	0.023	ND	ND	ND	ND
RSD_r_, %	3.80	2.59	ND	ND	2.45	2.40	ND	ND	ND	ND		ND	4.84	ND	ND	ND	ND
Rec., %	98.63	114.94	ND	ND	110.34	118.04	ND	ND	ND	ND		ND	100.35	ND	ND	ND	ND
Bias, µg/kg	−0.0014	0.013	ND	ND	0.019	25.44	ND	ND	ND	ND		ND	0.0016	ND	ND	ND	ND

Cereal-based infant food (non-dairy)—FAPAS T04424QC—method developer																	

Mean, µg/kg	0.203	0.160	0.205	0.160^c^	0.568	ND	ND	ND	ND	ND		ND	0.533	ND	ND	ND	ND
S_r_, µg/kg	0.0073	0.0072	0.010	0.007	0.022	ND	ND	ND	ND	ND		ND	0.022	ND	ND	ND	ND
RSD_r_, %	3.61	4.53	4.68	4.26	3.84	ND	ND	ND	ND	ND		ND	4.17	ND	ND	ND	ND
Rec., %	97.35	110.20	96.78	NA	87.31	ND	ND	ND	ND	ND		ND	102.76	ND	ND	ND	ND
Bias, µg/kg	−0.0055	0.015	−0.0068	NA	−0.083	ND	ND	ND	ND	ND		ND	0.014	ND	ND	ND	ND

Animal feed (DDGS)—Trilogy 121229(MM)—method developer																	

Mean, µg/kg	16.46	1.33	0.219^c^	ND	17.80	2847.95	9661.62	3313.30	958.12	13933.04		12974.92	2.75^c^	24.06^c^	85.35^c^	109.40^c^	159.65
S_r_, µg/kg	3.03	0.17	0.059	ND	3.20	86.83	570.13	105.04	60.63	641.84		603.92	0.29	1.62	2.48	3.20	9.28
RSD_r_, %	18.43	12.81	26.9	ND	17.98	3.05	5.90	3.17	6.33	4.61		4.65	10.51	6.73	2.91	2.93	5.81
Rec., %	94.06	121.33	NA	ND	95.67	105.48	115.02	110.44	119.77	114.21		NA	NA	NA	NA	NA	109.35
Bias, µg/kg	−1.04	0.23	NA	ND	−0.805	147.95	1261.62	313.30	158.12	1733.04		NA	NA	NA	NA	NA	13.65

Animal feed (DDGS)—Trilogy 121229(MM)—independent laboratory																	

Mean, µg/kg	10.02	0.83	ND	ND	10.85	2252.2	8150.00	2807.00	779.97	11736.00		10957.00	2.80^c^	14.24^c^	73.33^c^	87.56^c^	96.68
S_r_, µg/kg	2.13	0.21	ND	ND	2.34	895.40	1069.00	307.60	38.85	1405.00		1370.74	0.38	4.48	22.71	26.92	23.25
RSD_r_, %	21.21	25.69	ND	ND	21.53	39.76	13.11	10.96	4.98	11.97		12.51	13.66	31.45	30.97	30.74	24.04
Rec., %	57.26	75.32	ND	ND	58.32	83.41	97.02	93.56	97.50	96.20		NA	NA	NA	NA	NA	66.22
Bias, µg/kg	−7.48	−0.27	ND	ND	−7.75	−447.85	−250.00	−193.00	−20.04	−464.00		NA	NA	NA	NA	NA	−49.33

Cereal-based dog kibble—FAPAS T04447QC—method developer																	

Mean, µg/kg	13.38	ND	ND	ND	13.38^c^	1373.62	198.60	213.38	ND	411.98		411.98	10.82	54.99	60.79	115.78	116.68
S_r_, µg/kg	0.403	ND	ND	ND	0.403	19.23	9.06	9.01	ND	13.29		13.29	0.40	1.61	1.63	2.96	2.65
RSD_r_, %	3.01	ND	ND	ND	3.01	1.40	4.56	4.22	ND	3.23		3.23	3.65	2.93	2.68	2.55	2.26
Rec., %	122.79	ND	ND	ND	NA	126.02	86.35	93.18	ND	NA		94.06	108.16	103.95	108.56	110.27	100.74
Bias, µg/kg	2.48	ND	ND	ND	NA	283.62	−31.40	−15.62	ND	NA		−26.02	0.82	2.09	4.79	10.78	0.86

Cereal-based dog kibble—FAPAS T04447QC—independent laboratory																	

Mean, µg/kg	11.45	ND	ND	ND	11.45^c^	1453.4	160.78	173.64	ND	334.4		334.4	8.34	50.17	55.62	105.79	95.2
S_r_, µg/kg	0.12	ND	ND	ND	0.12	73.40	3.32	4.26	ND	7.24		7.24	0.37	1.47	1.95	3.38	5.09
RSD_r_, %	1.06	ND	ND	ND	1.06	5.05	2.06	2.45	ND	2.17		2.17	4.46	2.93	3.51	3.20	5.34
Rec., %	105.09	ND	ND	ND	NA	133.34	69.90	75.83	ND	NA		72.86	83.38	94.84	99.32	97.14	82.07
Bias, µg/kg	0.55	ND	ND	ND	NA	363.40	−69.23	−55.36	ND	NA		−124.58	−1.66	−2.73	−0.38	−3.11	−20.8

Paprika—FAPAS T04416QC—method developer																	

Mean, µg/kg	4.10	1.78	1.70	0.799	8.38	ND	ND	ND	ND	ND		ND	15.84	ND	ND	ND	ND
S_r_, µg/kg	0.489	0.183	0.213	0.087	0.97	ND	ND	ND	ND	ND		ND	1.85	ND	ND	ND	ND
RSD_r_, %	11.93	10.26	12.60	10.84	11.52	ND	ND	ND	ND	ND		ND	11.69	ND	ND	ND	ND
Rec., %	93.55	96.99	90.93	99.90	95.46	ND	ND	ND	ND	ND		ND	99.62	ND	ND	ND	ND
Bias, µg/kg	−0.28	−0.055	−0.170	−0.0008	−0.398	ND	ND	ND	ND	ND		ND	−0.061	ND	ND	ND	ND

Paprika—ERM BD286—method developer																	

Mean, µg/kg	2.84	0.146^f^	1.98	ND	4.82^f^	ND	ND	ND	ND	ND		ND	9.30^f^	ND	ND	ND	ND
S_r_, µg/kg	0.19	0.016	0.15	ND	0.20	ND	ND	ND	ND	ND		ND	0.57	ND	ND	ND	ND
RSD_r_, %	6.80	10.68	7.46	ND	4.14	ND	ND	ND	ND	ND		ND	6.18	ND	ND	ND	ND
Rec., %	76.43	NA	82.50	ND	NA	ND	ND	ND	ND	ND		ND	NA	ND	ND	ND	ND
Bias, µg/kg	−0.88	NA	−0.42	ND	NA	ND	ND	ND	ND	ND		ND	NA	ND	ND	ND	ND

Chili powder—FAPAS TET016RM—method developer																	

Mean, µg/kg	4.13	1.71	3.37	0.878	10.08	ND	ND	ND	ND	ND		ND	9.90	ND	ND	ND	ND
S_r_, µg/kg	0.33	0.11	0.31	0.073	0.803	ND	ND	ND	ND	ND		ND	1.78	ND	ND	ND	ND
RSD_r_, %	8.03	6.16	9.13	8.29	7.96	ND	ND	ND	ND	ND		ND	17.79	ND	ND	ND	ND
Rec., %	97.62	98.70	96.00	99.78	98.87	ND	ND	ND	ND	ND		ND	97.01	ND	ND	ND	ND
Bias, µg/kg	−0.101	−0.022	−0.140	−0.0019	−0.116	ND	ND	ND	ND	ND		ND	−0.305	ND	ND	ND	ND

a Total fumonisins.

b Fumonisins B_1_ + B_2_.

c No assigned value.

d NA = Not applicable as no assigned value for comparison.

e ND = Not detected.

f No certified value.

**Table 7.** Method developer matrix LOQ study results

Analyte		AFB_1_	AFB_2_	AFG_1_	AFG_2_	AFM_1_	DON	FB_1_	FB_2_	FB_3_	OTA	T-2	HT-2	ZON
*n*		10	10	10	10	10	10	10	10	10	10	10	10	10
Spike μg/kg		0.035	0.070	0.035	0.070	NT^a^	4.00	10.00	8.00	4.00	0.300	1.00	1.40	None
Corn	Mean	0.034	0.077	0.034	0.076	NT	4.23	8.78	6.87	3.72	0.325	0.997	1.61	5.44
	RSD_r_	11.36	6.02	7.41	6.84	NT	2.95	8.62	6.39	7.80	4.06	5.07	5.44	5.71
	Recovery	98.09	110.05	98.16	108.28	NT	105.84	87.78	85.85	92.90	108.34	99.67	114.94	—^b^
	LOQ	0.040	0.070	0.040	0.070	NT	4.00	10.00	8.00	4.00	0.30	1.00	1.40	5.44
	LOD_est_	0.013	0.023	0.013	0.023	NT	1.33	3.33	2.67	1.33	0.100	0.332	0.467	1.81
Spike μg/kg		0.035	0.070	0.035	0.070	NT	None	10.00	8.00	4.00	0.300	1.00	1.40	2.50
Wheat	Mean	0.029	0.079	0.034	0.073	NT	11.19	7.61	5.35	2.84	0.319	0.97	2.20	1.91
	RSD_r_	13.74	8.91	9.63	4.60	NT	5.46	10.45	10.63	9.49	10.53	9.82	11.64	8.06
	Recovery	83.90	112.71	97.04	103.89	NT	—	76.06	66.93	71.12	106.26	96.94	157.38	76.27
	LOQ	0.040	0.070	0.040	0.070	NT	11.19	10.00	8.00	4.00	0.300	1.00	1.40	2.50
	LOD_est_	0.013	0.023	0.01	0.023	NT	3.73	3.33	2.67	1.33	0.100	0.333	0.467	0.833
Spike μg/kg		0.018	0.035	0.018	0.035	NT	None	None	4.00	2.00	0.15	0.50	0.70	1.25
Non-dairy cereal baby food	Mean	0.018	0.039	0.019	0.038	NT	7.10	4.90	4.15	2.10	0.205	0.553	0.907	1.07
	RSD_r_	9.70	5.86	6.80	6.52	NT	4.15	6.55	3.44	3.43	3.59	3.02	4.14	3.90
	Recovery	105.40	111.57	110.71	108.54	NT	—	—	103.67	105.12	136.98	110.66	129.59	85.33
	LOQ	0.018	0.039	0.019	0.038	NT	7.10	4.90	4.00	2.00	0.150	0.500	0.700	1.25
	LOD_est_	0.006	0.013	0.006	0.013	NT	2.37	1.63	1.33	0.667	0.050	0.167	0.233	0.417
Spike μg/kg		0.018	0.035	0.018	0.035	0.035	None	5.00	4.00	2.00	0.15	0.50	0.70	None
Dairy cereal baby food	Mean	0.024	0.037	0.019	0.036	0.036	6.32	4.89	3.91	1.80	0.230	0.516	0.673	2.45
	RSD_r_	6.40	7.61	7.24	6.50	4.29	5.51	7.24	4.83	8.55	4.98	2.43	8.27	3.60
	Recovery	135.00	105.10	111.18	103.66	102.07	—	97.75	97.75	90.23	153.53	103.24	96.07	—
	LOQ	0.024	0.037	0.019	0.036	0.036	6.32	5.00	4.00	2.00	0.150	0.500	0.700	2.45
	LOD_est_	0.008	0.012	0.006	0.012	0.012	2.11	1.67	1.33	0.667	0.050	0.167	0.233	0.818
Spike, μg/kg		0.14	0.28	0.14	0.28	NT	None	None	32.00	16.00	1.50	7.50	7.50	7.00
Animal feed	Mean	0.139	0.267	0.145	0.300	NT	75.26	37.42	32.58	16.77	1.42	8.41	11.47	10.42
	RSD_r_	7.60	9.00	7.00	6.90	NT	6.60	12.68	5.87	8.25	4.29	5.44	6.94	14.31
	Recovery	99.40	95.30	103.70	107.20	NT	—	—	101.81	104.83	94.80	112.15	152.95	148.89
	LOQ	0.139	0.280	0.140	0.280	NT	75.26	37.42	32.00	16.00	1.50	7.50	7.50	7.00
	LOD_est_	0.046	0.093	0.047	0.093	NT	25.09	12.47	10.67	5.33	0.500	2.50	2.50	2.33
Spike, μg/kg		0.07	0.14	0.07	0.14	NT	8.00	20.00	16.00	8.00	None	2.00	3.00	5.00
Paprika	Mean	0.073	0.122	0.060	0.127	NT	7.73	16.13	12.55	6.92	2.14	1.71	3.07	5.25
	RSD_r_	18.30	13.60	15.27	10.52	NT	3.86	7.48	7.80	6.74	23.44	20.37	9.61	15.98
	Recovery	104.80	87.39	85.84	90.73	NT	96.57	80.63	78.45	86.52	—	85.48	102.27	105.06
	LOQ	0.073	0.140	0.070	0.140	NT	8.00	20.00	16.00	8.00	2.14	2.00	3.00	5.00
	LOD_est_	0.024	0.047	0.023	0.047	NT	2.67	6.67	5.33	2.67	0.715	0.667	1.00	1.67
Spike, μg/kg		None	0.14	0.07	0.14	NT	8.00	20.00	16.00	8.00	None	2.00	3.00	none
Chili	Mean	0.318	0.141	0.066	0.129	NT	8.08	15.35	13.50	7.19	4.39	2.01	3.55	6.30
	RSD_r_	10.48	3.87	7.50	4.02	NT	6.51	7.04	5.55	7.39	20.35	4.65	4.86	17.10
	Recovery	—	100.80	94.80	92.36	NT	100.95	76.74	84.40	89.84	—	100.74	118.35	—
	LOQ	0.320	0.140	0.070	0.140	NT	8.00	20.00	16.00	8.00	4.39	2.00	3.00	6.30
	LOD_est_	0.107	0.047	0.023	0.047	NT	2.67	6.67	5.33	2.67	1.46	0.667	1.00	2.10

a NT = not tested.

b – = not spiked so no recovery value.

**Table 8.** Independent laboratory matrix LOQ study results

Analyte		AFB_1_	AFB_2_	AFG_1_	AFG_2_	DON	FB_1_	FB_2_	FB_3_	OTA	T-2	HT-2	ZON
*n*		10	10	10	10	10	10	10	10	10	10	10	10
Spike, μg/kg		0.070	None	0.070	0.070	18.00	16.32	8.64	3.84	0.380	1.92	1.92	None
Corn	Mean	0.050	0.067	0.060	0.070	15.59	11.50	6.29	2.83	0.370	1.63	2.07	3.95
	RSD_r_	9.69	0.01	13.29	10.41	8.83	9.51	6.80	10.88	10.19	8.44	54.47	12.82
	Recovery	66.94	—^a^	70.08	85.05	85.29	63.30	62.22	66.72	74.03	77.57	134.59	—
	LOQ	0.070	0.070	0.070	0.070	18.00	16.32	8.64	3.84	0.38	1.92	1.92	3.95
	LOD_est_	0.023	0.023	0.023	0.023	6.00	5.44	2.88	1.28	0.127	0.640	0.640	1.32
Spike, μg/kg		0.144	None	0.144	0.144	None	None	None	7.68	0.768	3.84	None	7.20
Animal feed	Mean	0.110	0.130	0.110	0.140	76.84	34.60	8.24	6.63	0.670	2.95	4.44	6.92
	RSD_r_	12.43	14.13	17.85	14.41	5.76	26.06	26.51	17.56	12.83	13.04	42.56	12.85
	Recovery	66.87	—	65.82	86.22	—	—	—	41.93	61.77	56.26	—	54.26
	LOQ	0.140	0.130	0.140	0.140	76.84	34.60	8.24	7.68	0.770	3.84	4.44	7.20
	LOD_est_	0.047	0.043	0.047	0.047	25.61	11.53	2.75	2.56	0.257	1.28	1.48	2.40

a — = Not spiked so no recovery value.

Table 4 shows the results for spiked corn samples were marginally outside specification for recoveries (too high); however, the RSD_r_ values showed the precision was acceptable. With the exception of marginally high recovery from the low DON spike, mean recoveries from the three spike levels from the same corn sample tested by the independent laboratory were within specification and RSD_r_ values met criteria (19). Mean results for all analytes measured by the method developer and independent laboratory from corn reference sample TCL0406QC (Table 5) were acceptable. Recovery values ranged from 83.38% for fumonisin B_1_ to 120.35% for T-2 toxin. Repeatability values (RSD_r_) were within criteria as were the recovery values except for DON (112.66%) and T-2 (120.35%) in the method developer study. Although no reference value was provided for fumonisin B_3_ in sample TCL0406QC, it was reported in both method developer and independent laboratory results at levels of 13.74 µg/kg and 14.66 µg/kg, respectively.

For wheat (Table 4), mean aflatoxin G_2_ (123.62%), OTA (128.68%), and DON (115.59%) recoveries were high for the low spike (level 1) conditions. The other recoveries were within the acceptable range as were all the RSD_r_ values. Two wheat reference samples were tested. The assigned analytes detected from FAPAS sample T22187QC met mean recovery and RSD_r_ specifications (Table 5) apart from DON, which was slightly above 110% recovery with a mean of 111.83%. RSD_r_ values ranged from 1.94 to 5.30%. For Trilogy sample TP-QC-MYC-20–1-WHE, mean recoveries of DON (141.03%), fumonisin B_1_ (112.5%), and HT-2 (128.22%) were outside the OMA range. High RSD_r_ values were recorded for AFB_1_ (38.19%) and OTA (23.03%), although mean recoveries (112.23% and 114.81%, respectively) were within specification. Low levels of AFB_2_ and AFG_1_ were also detected in this sample (no reference values were assigned for these analytes).

Method developer study results for non-dairy cereal-based baby food (Table 4) showed the presence of native DON, fumonisin B_1_, fumonisin B_3_, OTA, and HT-2 at less than the lowest calibration standard for unspiked samples. Except for OTA at the low spiking level (127.32%), mean recovery levels for all three spiking levels were within criteria. The RSD_r_ range was 1.41–9.21%. Two reference materials were tested (Table 5). FAPAS T04412QC recovered all analytes, which were assigned reference values in the recovery range 98.63 to 118.04%, with RSD_r_ values between 2.40 and 4.84%. DON recovery (118.04%) was the only outlier in the dataset. For FAPAS T04424QC, mean recoveries ranged from 87.31 to 110.2% and RSD_r_ values were between 3.61 and 4.68%. In both samples, OTA was recovered at levels close to the assigned value and with RSD_r_ values of 4.84% and 4.17%, respectively. All other assigned analytes were detected within specifications for both samples. Although not assigned a value, sample TO4424QC was also found to have low levels of AFG_2_.

Dairy cereal-based baby food (Table 4) showed the presence of native AFB_1,_ OTA, and HT-2 in blank test portions at levels less than calibration standard 1. Mean OTA recovery was high from level 1 (138.0%) and level 2 (126.4%) spiked test portions. All the other recovery values were within range and all RSD_r_ values were acceptable ranging from 1.39% for ZON (level 1) to 8.27% for AFB_2_ (level 2). Finally, AFM_1_ spiking results were consistently within specification for the three levels tested, demonstrating the method as suitable for accurate and precise detection of AFM_1_ in baby food.

Method developer results for animal feed showed the presence of native DON, fumonisin B_1_, and ZON in the un-spiked sample. ZON was measured in two of the six test portions (Table 4). Mean recoveries of DON (111.63%) marginally exceeded OMA criteria in the level 1 spike animal feed sample as did T-2 at level 3 (111.60%; *see*  Table 6). Other mean recoveries were within range and RSD_r_ values were all acceptable with values ranging from 1.89% (AFB_1_ level 2) to 7.09% (fumonisin B_3_ level 1). The independent laboratory results in Table 4 also showed the presence of native DON, fumonisin B_1_, and ZON in the un-spiked sample. In addition, low levels of native fumonisin B_2_, fumonisin B_3_, T-2, and HT-2 were also detected. Compared to method developer studies, overall mean recoveries were lower but fell within the acceptance criteria range (70.23 to 101.98%). RSD_r_ values were high for many of the analytes at the low spike level 1, ranging from 29.38% for AFG_2_ to 65.03% for DON. RSD_r_ values for fumonisins and OTA were acceptable. At spike level 2, the DON (32.14%), RSD_r_ value was above the acceptance criteria limit, similar to spike level 3 where the DON RSD_r_ was reported as 23.31%. The recoveries for test portions 4 and 5 at level 1 were significantly lower for DON, T-2, HT-2, ZON, and the aflatoxins compared with the other replicates. Something similar happened with test portion 1 of spike level 2 and test portion 5 of spike level 3. These results were responsible for the high RSD_r_ values (*see* raw data tables for animal feed), and also fell outside the precision criteria (16)

Method developer results for animal feed reference 121229(MM) were within specification of the assigned values except for AFB2, which was marginally above 120% recovery, and fumonisins B_1_ and B_3_, which were higher than 110% (Table 5). AFG_1_, OTA, T-2, and HT-2 did not have values assigned but were detected at low levels. The RSD_r_ values were acceptable for analytes that had a reference value assigned. Animal feed sample TO4447QC recoveries of DON at 126.02% were high and AFB1 at 122.79% marginally so, but all other analytes were detected within specification. The same reference samples were tested by the independent laboratory. Sample 1212299(MM) was shown to have detectable levels of the unassigned OTA, T-2, and HT-2. For analytes with assigned values, AFB_1_ and ZON recoveries (57.26% and 66.22%, respectively) were below the criteria levels (Table 5). As occurred with the spiked animal feed, one of the extracts (test portion 6) gave much lower recoveries for nearly all the analytes (including DON, ZON, AFB_1_, and AFB_2_ of the assigned analytes) that lowered their respective mean recovery and increased RSD_r_ values (*see* raw data tables). Except for DON (high at 133.34%) and fumonisin B_1_, (marginally low at 69.90%), the analytes were recovered within criteria from sample TO4447QC. RSD_r_ values were found to be within an acceptable range (1.06 to 5.34%). Overall, both laboratories reported similar findings for animal feed in both spiked samples and contaminated QC/reference materials.

Method developer results from the paprika sample (Table 4) showed aflatoxin B_1_ and ZON were detected in five of six test portions at levels under the lowest calibration standard. There were high mean recoveries for the level 1 HT-2 (129.3%) and OTA (117.60%) spike and DON in level 2 (114.96%) and level 3 (113.96%) spikes. All remaining analyte mean recoveries and RSD_r_ values for the spike levels tested were found to be acceptable. All reference analytes were recovered from paprika sample FAPAS T04416QC, meeting criteria for both mean recovery and RSD_r_. Mean recoveries of AFB_1_ (76.43%) and AFG_1_ (82.50%) with RSD_r_ values of 6.80% and 7.46%, respectively, for reference sample ERM BD286 were within acceptance criteria. OTA and AFB_2_ were detected (*see*  Table 5) but did not have assigned values.

Finally, matrix study results for chili showed levels of AFB_1_, OTA, HT-2, and ZON in the blank sample (*see*  Table 4). All analytes spiked at three separate levels showed mean recoveries (ranging from 72.89 to 114.54%) and RSD_r_ values (2.19 to 14.49%) that met the acceptance criteria. The recovery of all reference analytes from FAPAS sample TET016RM was close to the stated values (Table 5) and RSD_r_ values ranged from 6.16% (AFB_2_) to 17.97% (OTA).

**Figure 5.**
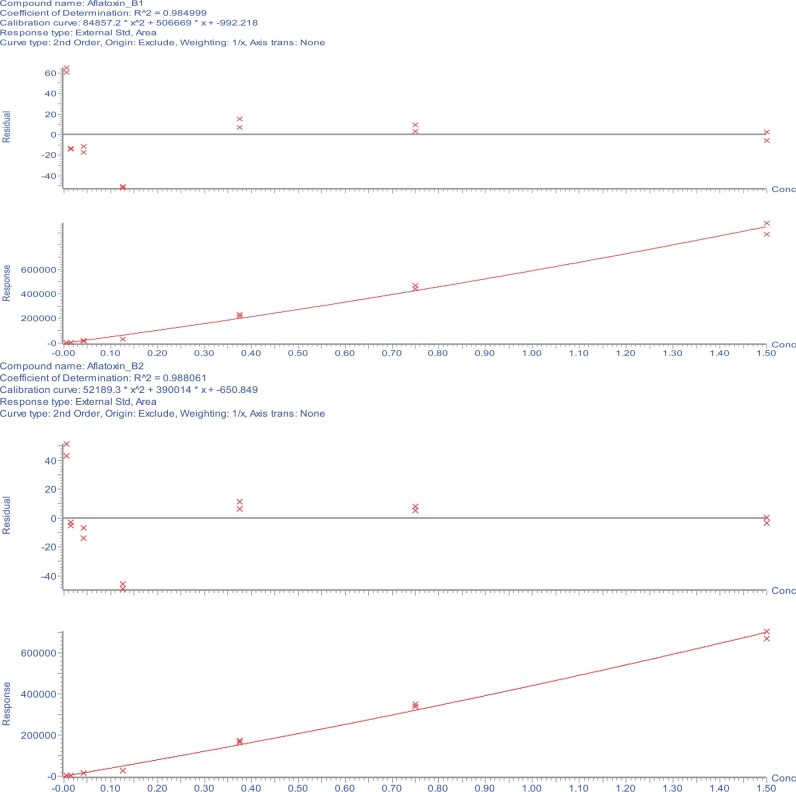

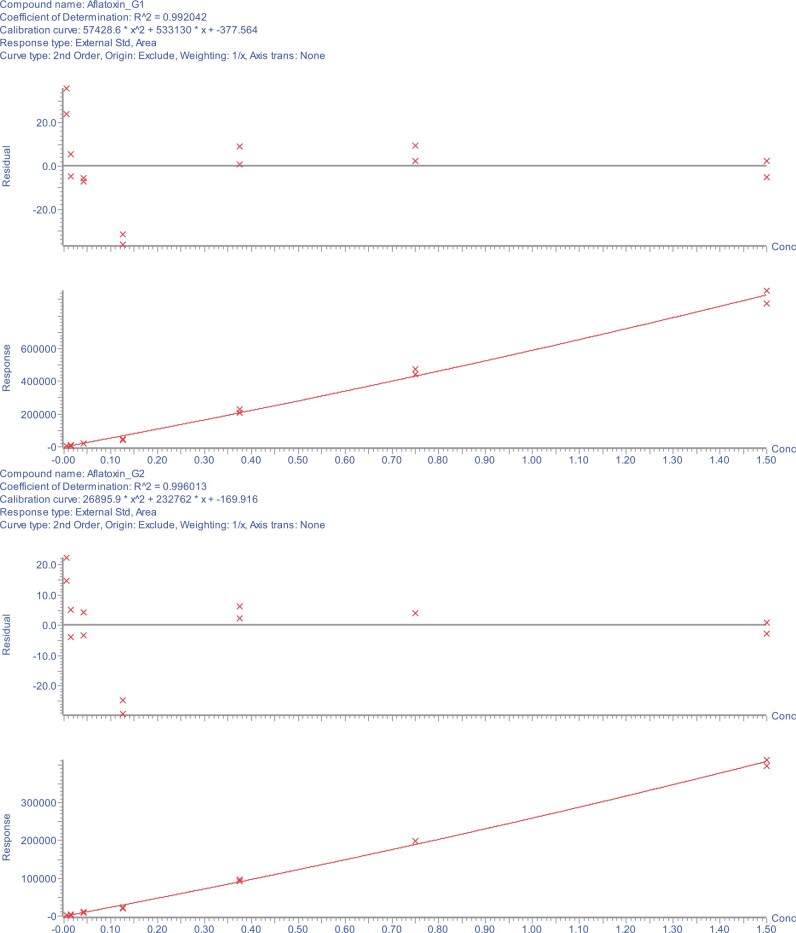

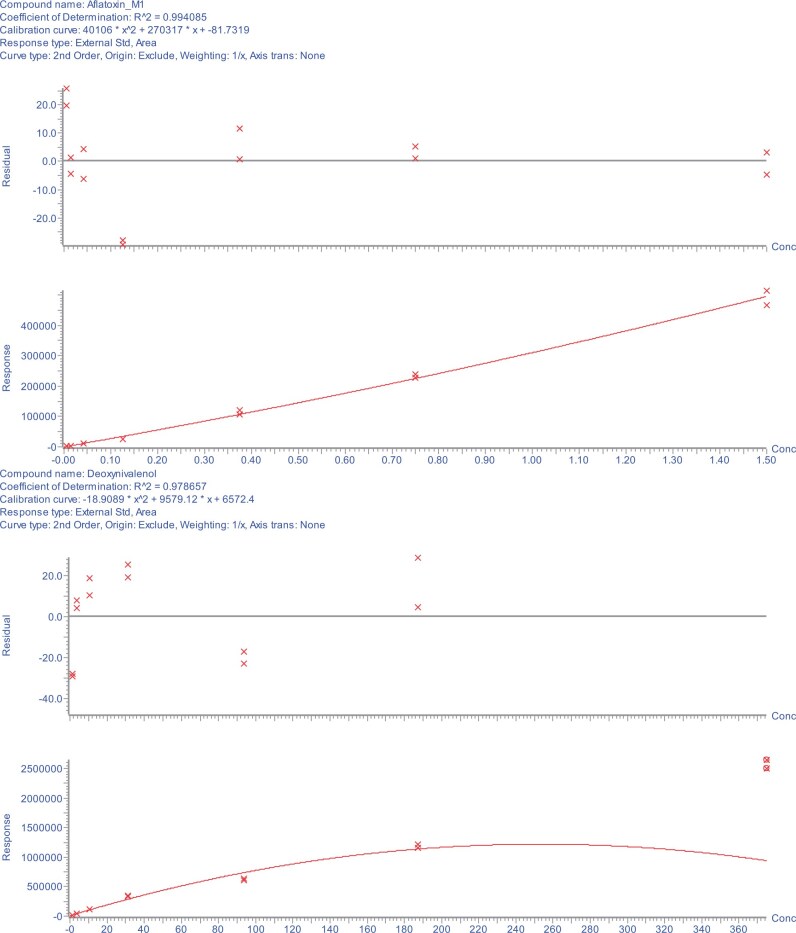

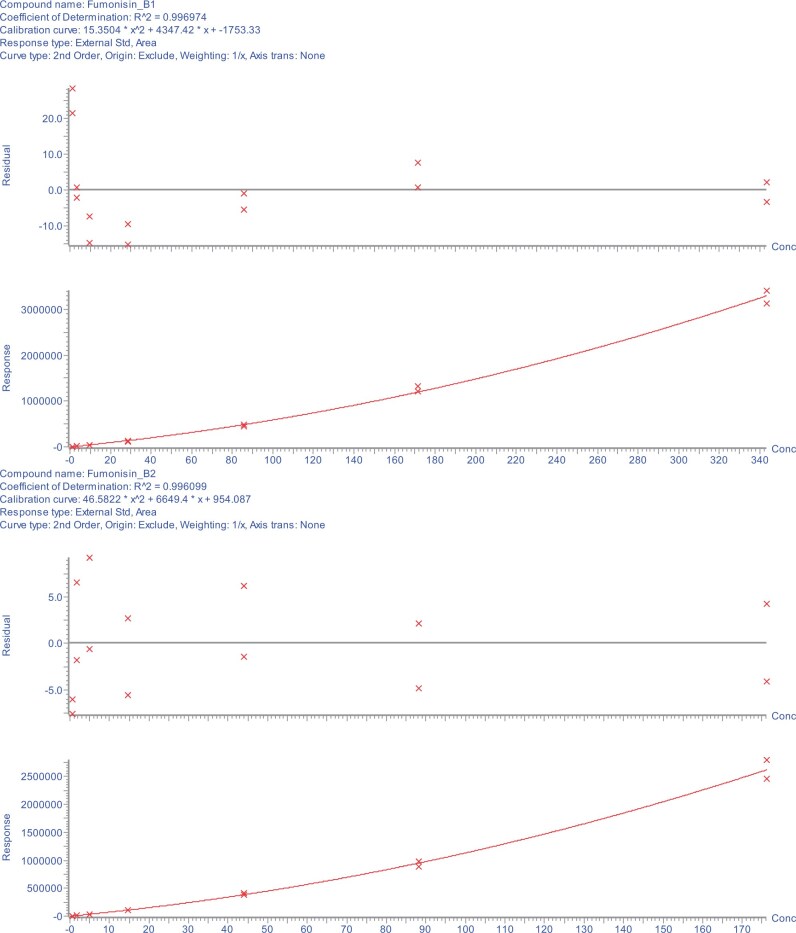

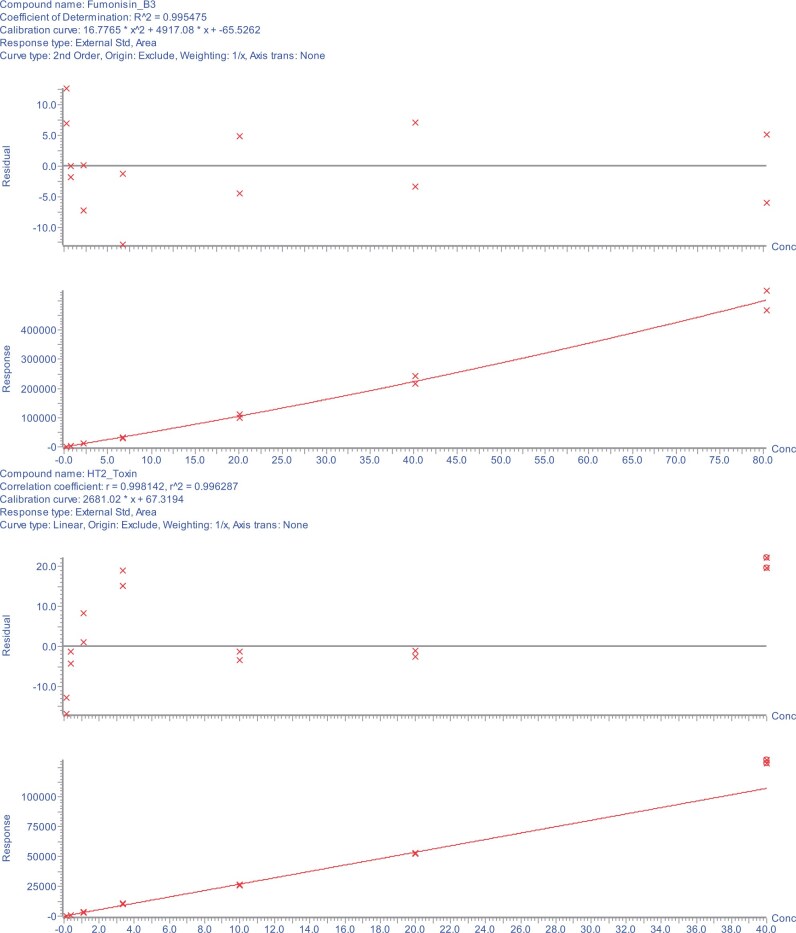

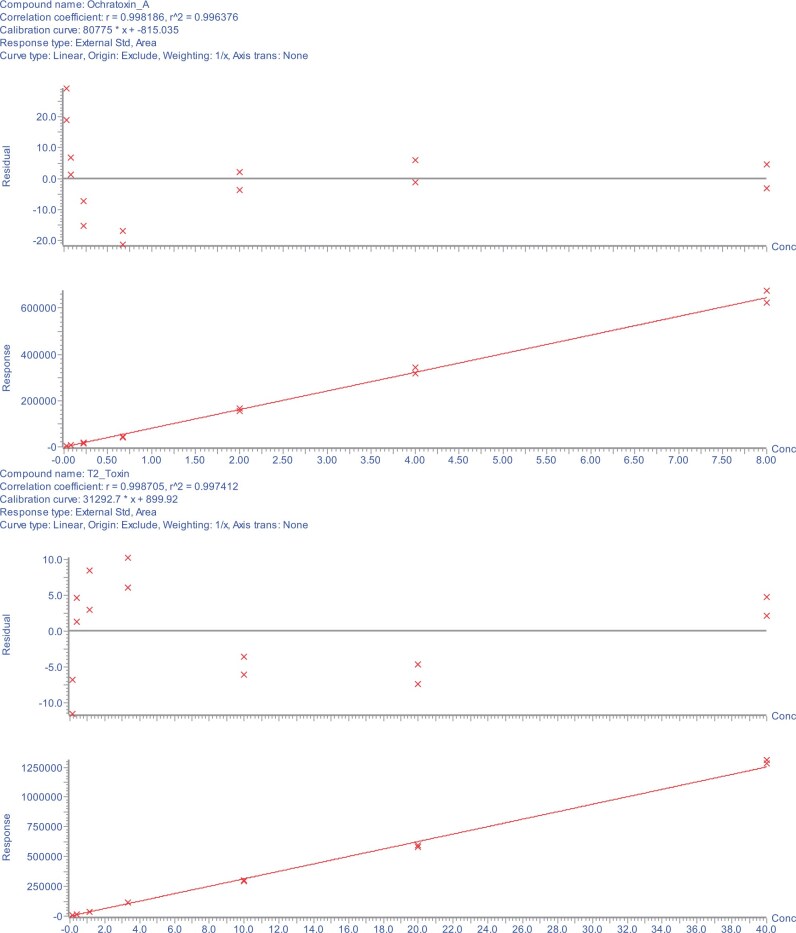

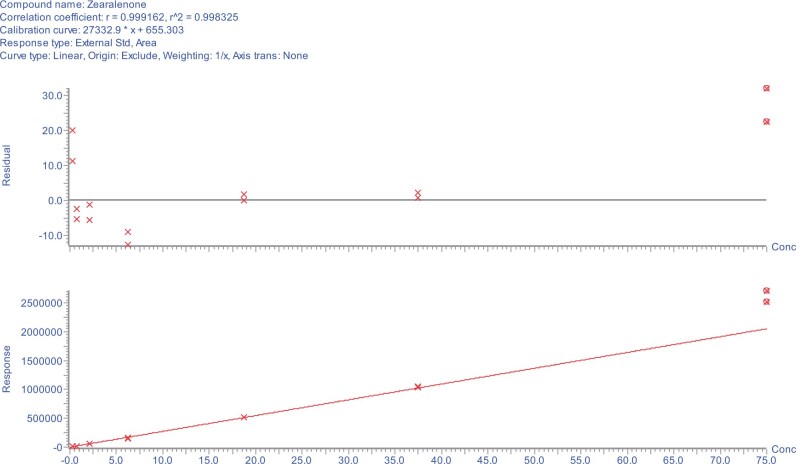
Calibration graphs and residual plots for Batch PC23-04296 corn from the independent laboratory.

**Figure 6.**
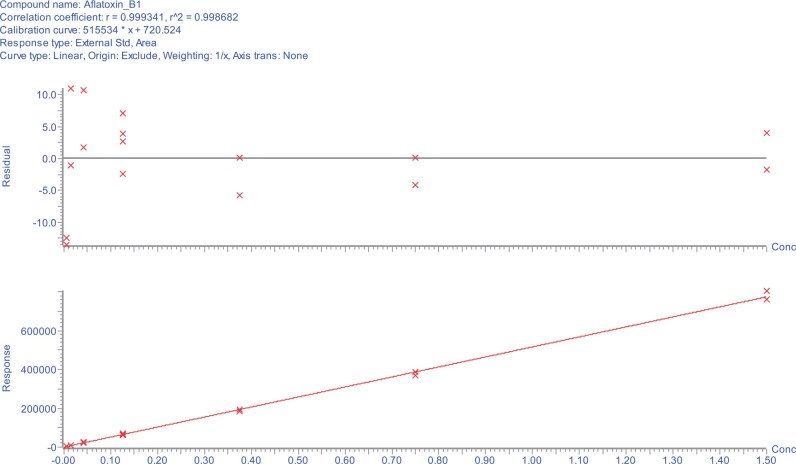

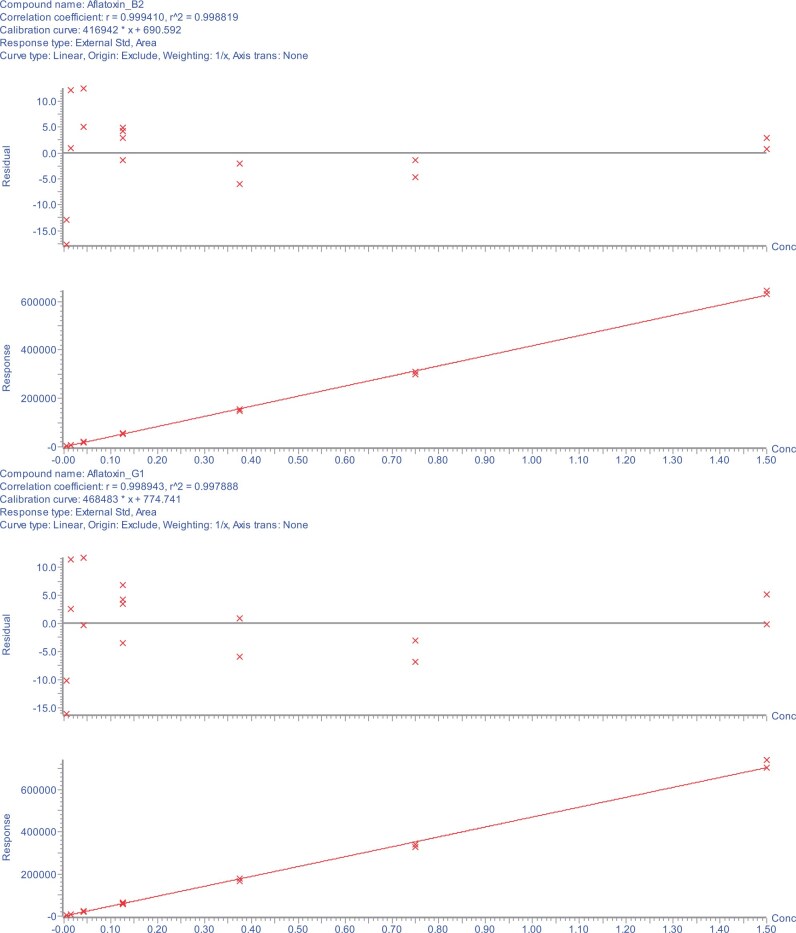

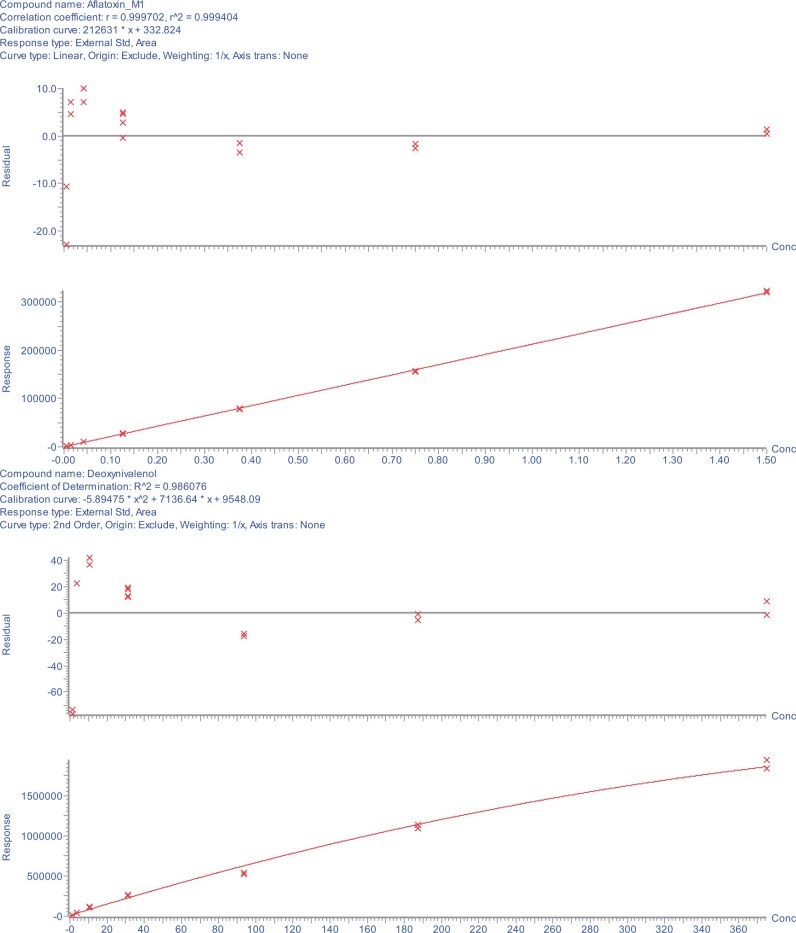

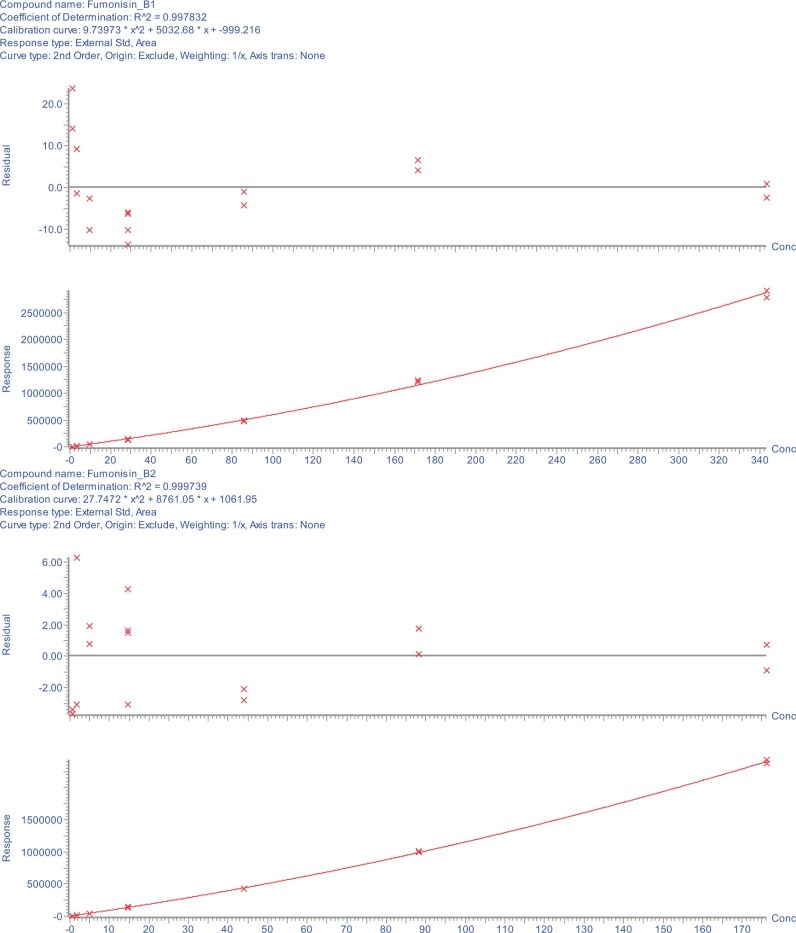

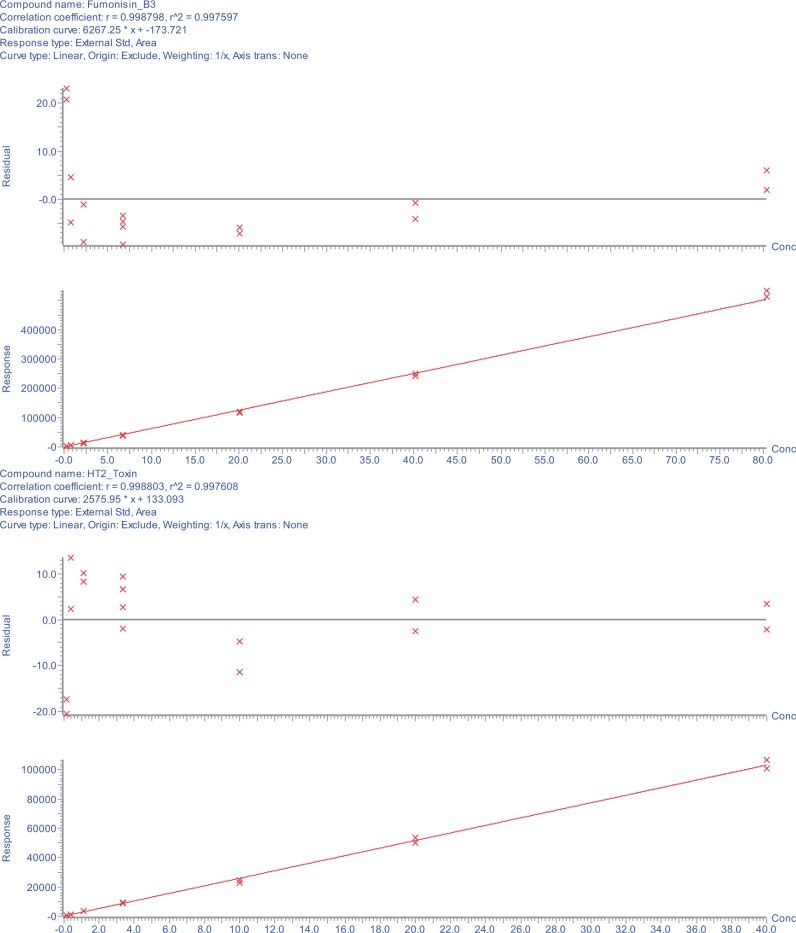

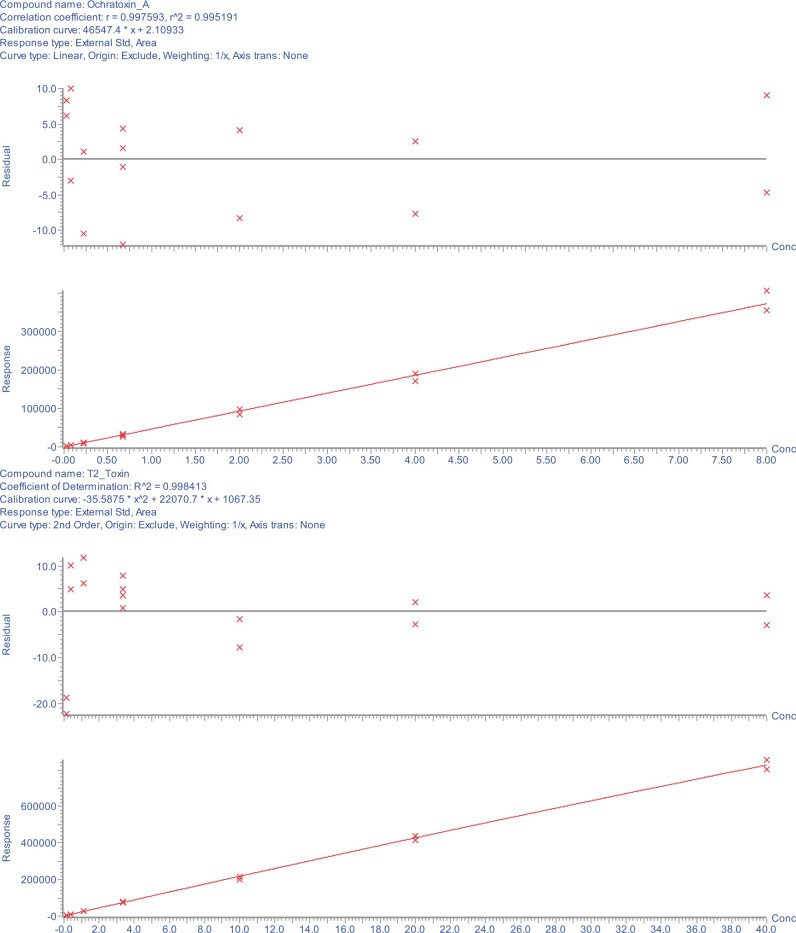

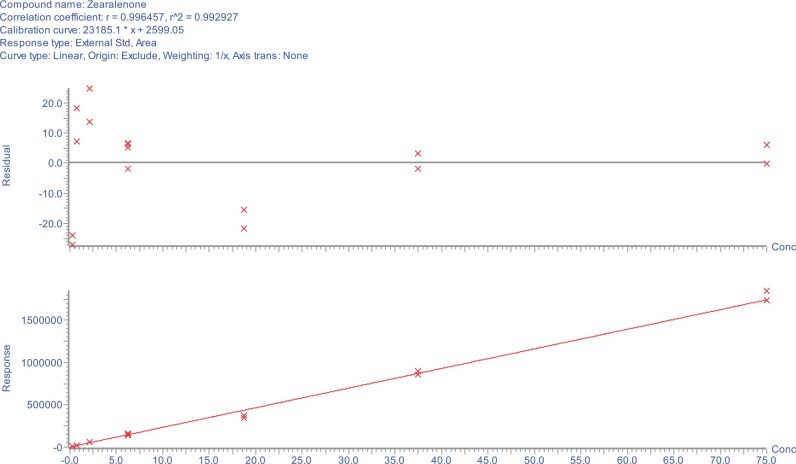
Calibration graphs and residual plots for Batch PC23-04421 animal feed from the independent laboratory.

**Figure 7.**
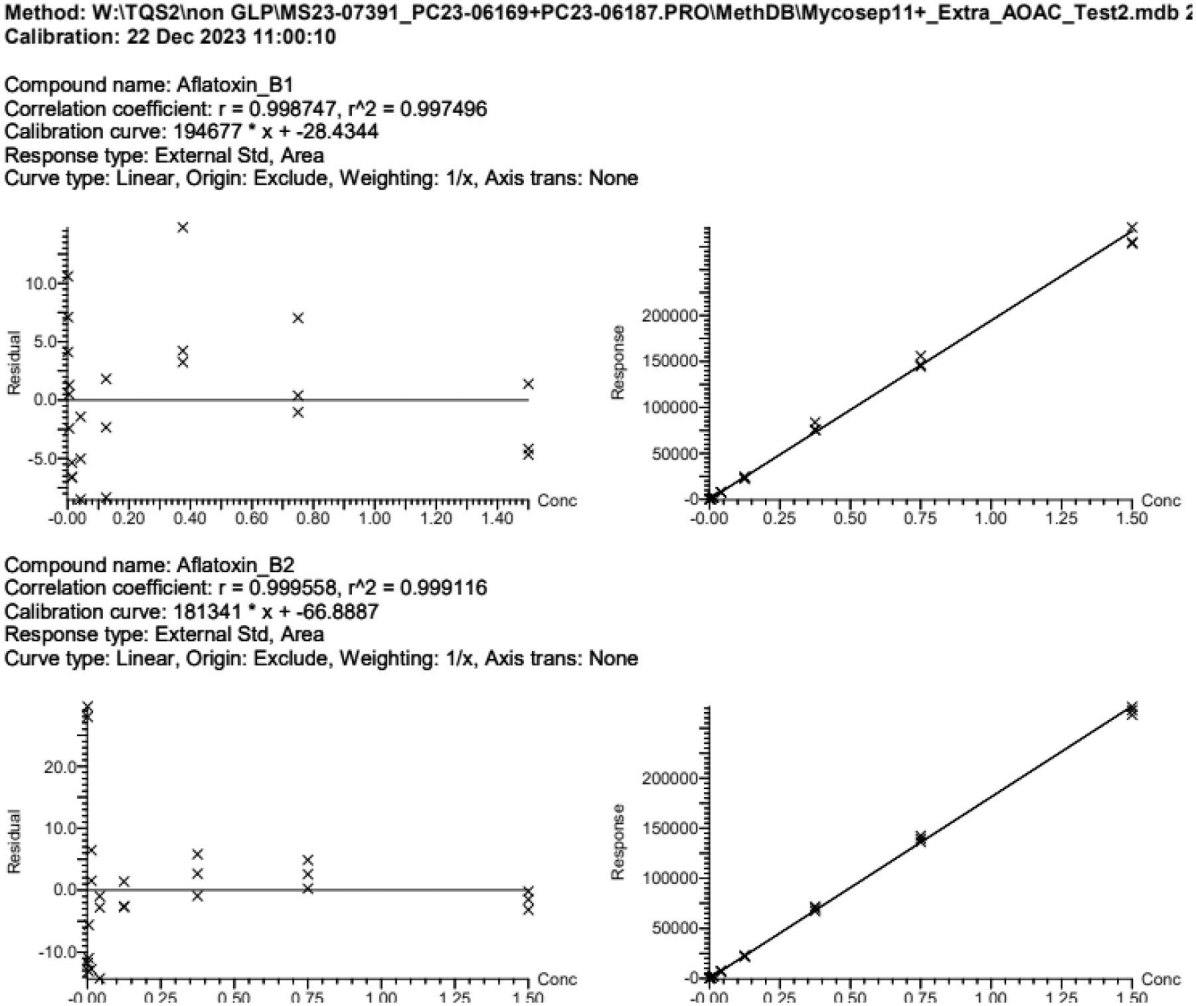

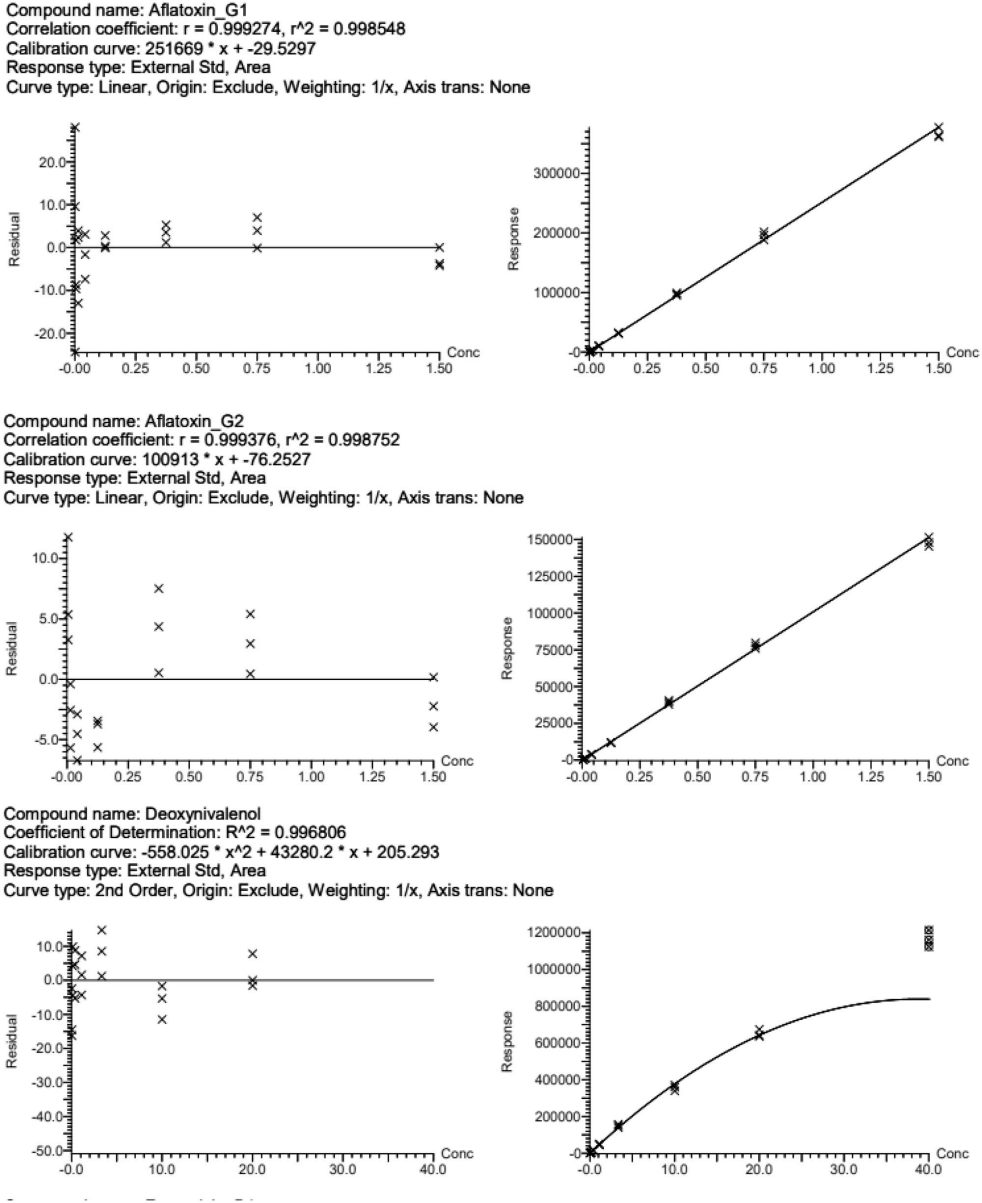

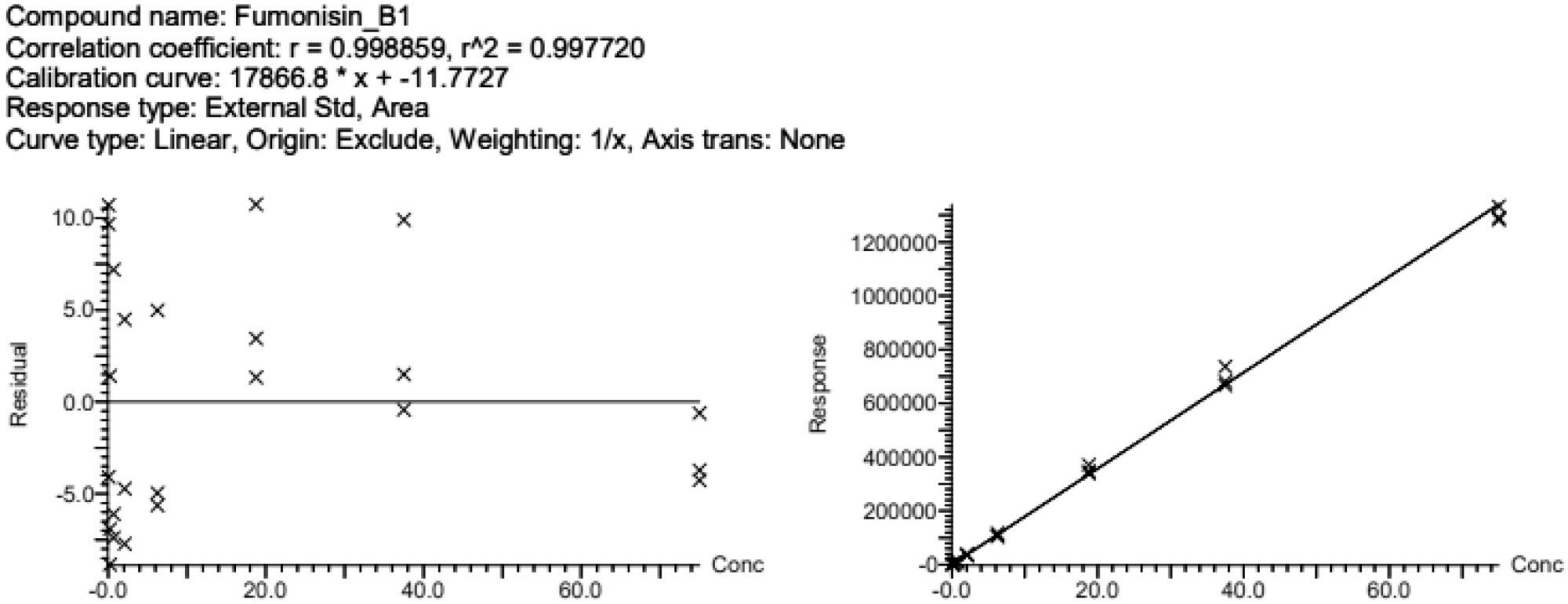

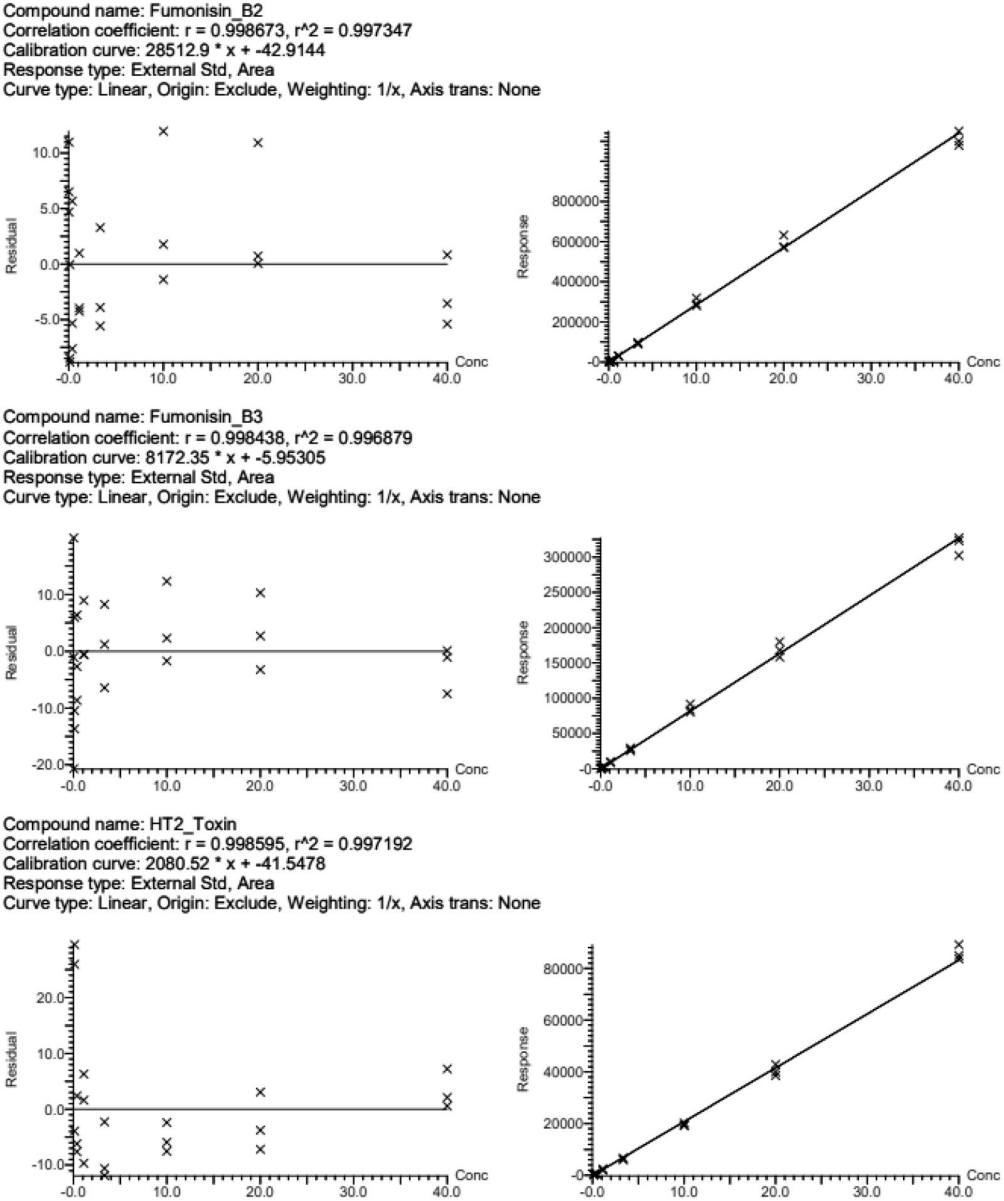

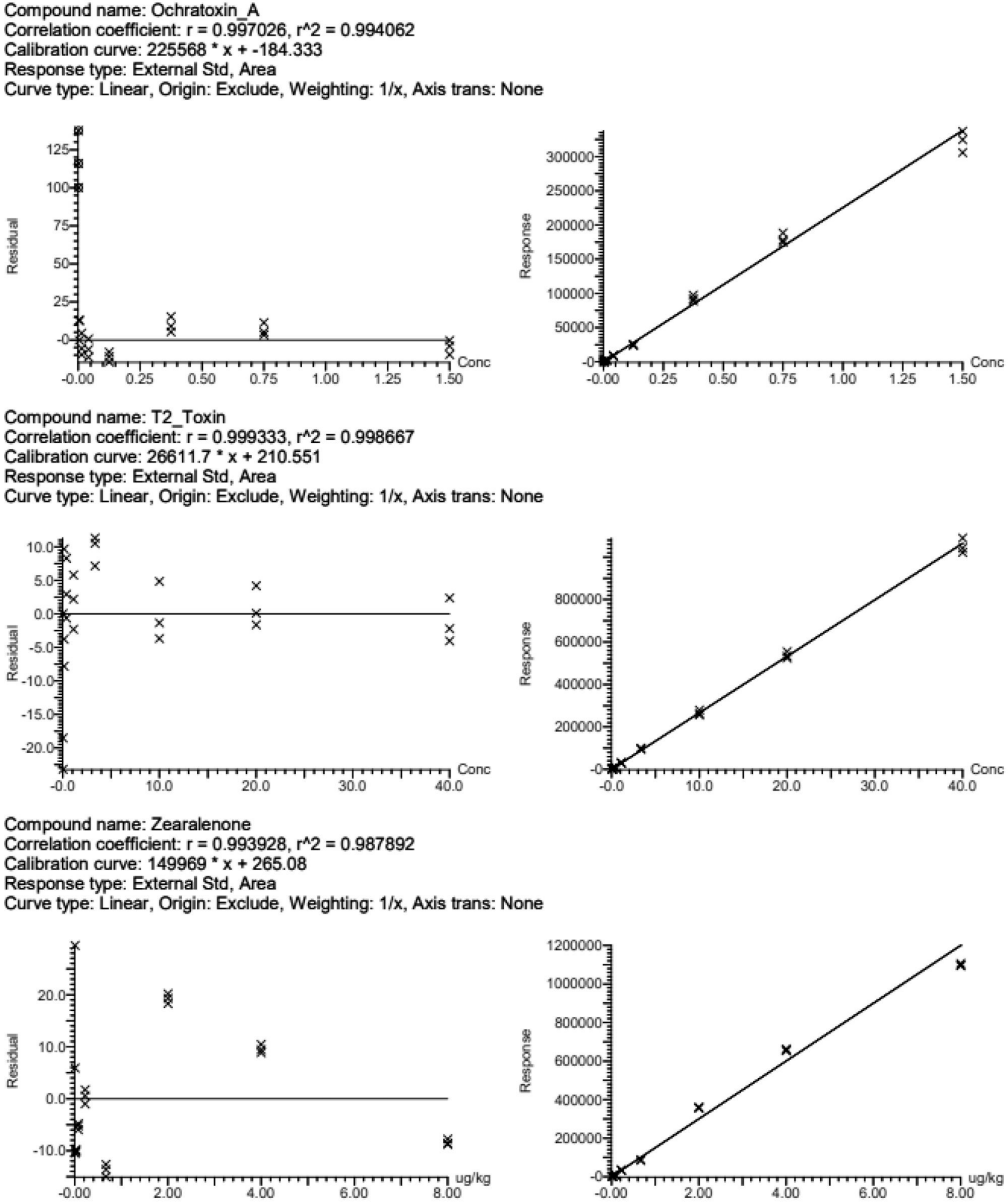
Calibration graphs and residual plots for LOQ studies from the independent laboratory.

Most of the method developer result outliers were found at level 1 (low) spikes. The results for corn showed the recoveries were marginally outside specification (too high); however, the RSD_r_ values showed the precision was acceptable, and with few exceptions, data from the independent laboratory (assaying the same sample) were within specification. Except for sporadic data points, the remaining claim matrixes were within the acceptance criteria for recovery and precision (19: *see*  Table 6). For animal feed and corn samples tested by the independent laboratory, the method was reported as easy to use, and conveniently and reliably determined a range of mycotoxins across a large range of concentrations in the two matrixes tested. The variable results (high RSD_r_ values) and lower recoveries observed for most of the analytes in the animal feed spike sample were thought to be due to problems encountered by loading and washing the columns when the animal feed sample extracts had not been filtered through glass microfiber paper. In most cases the results met the performance criteria. Specimen calibration and residual plots are shown in Figure 5 for the spiked corn sample batch, Figure 6 for the spiked animal feed sample batch, and Figure 7 for LOQ studies.

### LOD and LOQ

Corn and animal feed matrix samples were shared between method developer and independent laboratory sites (*see*  Tables 7 and 8). For corn, the LOQ was shown to be comparable for AFB_1_ (0.04–0.07 µg/kg), AFG_1_ (0.04–0.07 µg/kg) AFB_2_, and AFG_2_ at 0.07 µg/kg and for fumonisin B_2_ (8–8.64 µg/kg), fumonisin B_3_ (3.84–4 µg/kg), OTA (0.3–0.38 µg/kg), T-2 (1–1.92 µg/kg), HT-2 (1.4–1.92 µg/kg), and ZON (3.95–5.44 µg/kg). The independent laboratory LOQs for DON and fumonisin B_1_ were reported as 18 µg/kg and 16.32 µg/kg, compared to the method developer LOQs of 4 µg/kg, and 10 µg/kg, respectively. The animal feed LOQ was comparable for both method developer and independent laboratory for the following analytes: AFB_1_ (0.14 µg/kg), AFB_2_ (0.13–0.28 µg/kg), AFG_1_ (0.14 µg/kg), AFG_2_ (0.14–0.28 µg/kg), DON (75.26–76.84 µg/kg), fumonisin B_1_ (34.6–37.42 µg/kg), and ZON (7.00–7.2 µg/kg). There was a lower LOQ for the independent laboratory fumonisin B_2_ (8.24 µg/kg, compared to the method developer value of 16 µg/kg), fumonisin B_3_ (7.68–16 µg/kg), OTA (0.77–1.5 µg/kg), T-2 (3.84–7.5 µg/kg), and HT-2 (4.44–7.5 µg/kg). This was a result of differences in original LOQ estimations; however the estimated LOQ spikes were achieved in all cases.

Compared to the method developer data, lower recoveries were obtained for some analytes by the independent laboratory; however, with the exception of HT-2 in corn, Horowitz equation values were acceptable (HorRat ≤2), meeting precision criteria (16). The mean recovery for HT-2 in corn was reported as 134.59%, suggesting the LOQ could be comfortably achieved. Two of the ten replicates were outliers (0 values). Although small differences were observed, method developer and independent laboratory data showed LOQ levels exceeding minimum requirements set out by regulations and guidance, as summarized in Table 1, could be comfortably achieved. As an example, the lowest recommended T-2 level in animal feed is 50 µg/kg (20), compared to the LOQ demonstrated (3.84–7.5 µg/kg) in both the independent laboratory and method developer data.

For the remaining matrixes tested, method developer LOQ data showed LOQ levels exceeding minimum requirements set out by regulations and guidance, as summarized in Table 1. The LOQ of ZON in baby food was shown to be 1.25 µg/kg in non-dairy and 2.45 µg/kg in dairy cereal-based baby food where it was measured at native level. The regulated level in the EU is 20 µg/kg. The LOQ for OTA in paprika and chili (measured at native levels) of spices was shown to be 2.14 and 4.39 µg/kg, respectively. This compares to the regulated maximum EU level of 15–20 µg/kg. Aflatoxins were also quantifiable at levels much less than regulated. Method developer AFB_1_ LOQ in both corn and wheat was 0.035 µg/kg (the independent laboratory value was 0.07 µg/kg for corn). This compares to the regulated limit of 2–5 µg/kg in the EU (21). A 20 µg/kg limit exists for total aflatoxin in the United States. (4)

### Product Consistency and Stability

The method was used to assay FAPAS corn reference material TCL0406QC (Table 9) to determine lot-to-lot variability and product stability for three lots of 11^+^Myco MS-PREP columns. The batches tested were near the expiration date, near the middle of the expiration period, and recently manufactured. ANOVA analysis of data determined a low degree of variability between datasets indicating product consistency and stability over the assigned expiry of the columns (Table 9). All *P*-values were >0.05 so no statistically significant differences were observed. Consistent with all three lots tested, analyte recovery was within ranges set out by OMA criteria. DON recovery was slightly above the upper limit of the range in all three lots; however, RSD_r_ values were within criteria for all analytes tested. (19) The recommended shelf life of the 11^+^Myco MS-PREP columns is 18 months when stored at 2–8°C.

**Table 9.** Product consistency and stability

Method developer results TCL0406QC																			
Analyte	*n*	Lot JL 334					Lot LC 611					Lot LK 718					*F*-value	*Fcrit*-value	*P*-value
		Mean	S_r_	RSD_r_	Recovery	Bias	Mean	S_r_	RSD_r_	Recovery	Bias	Mean	S_r_	RSD_r_	Recovery	Bias			
AFB_1_	3	0.52	0.03	6.10	99.49	0.00	0.53	0.02	3.58	102.64	0.01	0.50	0.02	4.07	96.17	−0.02	1.44	5.14	0.31
AFB_2_	3	0.43	0.02	4.28	105.13	0.02	0.45	0.02	3.80	109.35	0.04	0.43	0.01	2.64	105.80	0.02	1.02	5.14	0.42
AFG_1_	3	0.53	0.02	3.14	106.65	0.03	0.54	0.02	3.79	107.40	0.04	0.53	0.01	1.79	106.13	0.03	0.12	5.14	0.89
AFG_2_	3	0.45	0.02	3.55	111.31	0.05	0.47	0.01	1.93	115.11	0.06	0.45	0.02	5.37	110.55	0.04	0.96	5.14	0.43
AFT	3	1.93	0.06	3.24	103.34	0.06	1.99	0.02	0.85	106.16	0.12	1.91	0.05	2.79	102.26	0.04	1.81	5.14	0.24
DON	3	346.99	2.36	0.68	116.44	48.99	357.55	4.48	1.25	119.98	59.55	345.71	14.65	4.24	116.01	47.71	1.58	5.14	0.28
FB_1_	3	132.63	1.33	1.00	99.72	−0.37	131.79	4.51	3.42	99.09	−1.21	130.43	3.14	2.41	98.07	−2.57	0.35	5.14	0.72
FB_2_	3	131.47	8.34	6.34	98.85	−1.53	135.91	6.79	5.00	102.19	2.91	126.32	14.57	11.54	94.98	−6.68	0.63	5.14	0.56
FB_3_	3	15.38	1.15	7.45	—^a^	—	15.64	0.48	3.08	—	—	14.92	0.77	5.15	—	—	0.55	5.14	0.60
FUM	3	264.11	7.60	2.88	104.39	11.11	267.71	11.30	4.22	105.81	14.71	256.75	14.31	5.57	101.48	3.75	0.73	5.14	0.52
OTA	3	0.56	0.03	5.48	108.13	0.04	0.57	0.00	0.49	109.18	0.05	0.55	0.02	3.06	105.94	0.03	0.54	5.14	0.61
T-2	3	22.47	0.56	2.51	110.69	2.17	22.87	0.94	4.12	112.67	2.57	22.53	0.63	2.81	110.99	2.23	0.26	5.14	0.78
HT-2	3	13.83	0.12	0.86	85.37	−2.37	13.21	0.67	5.04	81.52	−2.99	13.17	0.75	5.72	81.27	−3.03	1.22	5.14	0.36
Sum T-2 & HT-2	3	36.30	0.66	1.82	99.18	−0.30	36.08	1.60	4.43	98.57	−0.52	35.70	0.93	2.60	97.53	−0.90	0.22	5.14	0.81
ZON	3	30.60	1.11	3.64	102.68	0.80	31.36	0.78	2.48	105.25	1.56	30.57	0.74	2.43	102.59	0.77	0.76	5.14	0.51

a — = No assigned value.

### Robustness

Overall, the robustness results were largely unaffected by small variations in method parameters (Table 10). The mean and s_r_ for each analyte and each treatment combination were determined and data subjected to ANOVA analysis using Minitab software (*see*  Supplemental Tables 8–19). Aflatoxins B_1_, B_2_, G_1_, and G_2_ had significant *P*-values for the elution rate (G_1_ was borderline at 0.048). Increased elution flow rate was shown to decrease recovery of aflatoxins B_1_, B_2_, G_1_, and G_2_. For AFB_1_, the combination of extraction time with loading flow rate gave a *P*-value of 0.033. For DON, as the column loading flow rate increased (*P*-value 0.001) and the extraction time decreased (*P*-value 0.006), both caused a decrease in recovery. Finally, for HT-2 an increase in the column loading flow rate led to a significant decrease in recovery. In conclusion, some factors did show significant variation (*see*  Table 6 and Supplemental Figure 1). From all the variants noted, it is recommended that the loading flow rate should not be exceeded, as this had the greatest impact on results. In addition, elution off the column should not exceed the recommended elution rate as results may be compromised.

**Table 10.** Robustness results

Process parameters				Results from sample TCL0406QC, µg/kg											
Treatment combination	Extraction time, min	Column loading, mL/min	Column elution, drop/s	AFB_1_ (0.52)^a^	AFB_2_ (0.410)	AFG_1_ (0.5)	AFG_2_ (0.405)	OTA (0.520)	FB_1_ (133)	FB_2_ (133)	FB_3_ ^b^	DON (298)	ZON (29.8)	T-2 (20.3)	HT-2 (16.2)
1	25	1	0.5	0.508	0.435	0.511	0.471	0.491	126.79	126.91	13.80	332.36	34.36	24.20	13.22
	25	1	0.5	0.462	0.431	0.482	0.439	0.501	123.42	120.87	13.30	338.91	32.24	22.44	12.21
2	25	1	2	0.467	0.432	0.471	0.417	0.492	132.03	117.84	12.06	323.14	32.05	22.68	13.01
	25	1	2	0.440	0.383	0.467	0.434	0.470	144.76	126.89	14.62	333.10	30.72	22.05	13.05
3	25	3	0.5	0.482	0.433	0.525	0.436	0.511	132.20	120.15	13.25	318.66	31.54	23.06	12.09
	25	3	0.5	0.476	0.409	0.488	0.445	0.489	127.54	119.66	13.81	319.55	33.54	23.61	11.91
4	25	3	2	0.451	0.409	0.504	0.431	0.470	121.83	121.72	13.69	316.96	30.78	22.02	11.36
	25	3	2	0.459	0.415	0.478	0.426	0.478	120.85	124.53	12.71	317.04	32.71	23.64	11.43
5	35	1	0.5	0.461	0.426	0.484	0.433	0.478	128.23	114.94	14.39	339.12	33.76	22.50	13.17
	35	1	0.5	0.451	0.429	0.497	0.447	0.445	131.94	125.09	13.27	336.94	32.11	22.62	13.05
6	35	1	2	0.452	0.397	0.483	0.439	0.470	117.24	113.15	12.54	340.00	31.11	22.93	13.27
	35	1	2	0.416	0.421	0.485	0.445	0.458	117.31	114.30	13.01	341.98	32.61	23.00	12.63
7	35	3	0.5	0.485	0.426	0.524	0.426	0.511	133.49	124.51	15.50	316.57	32.57	22.83	11.43
	35	3	0.5	0.477	0.438	0.495	0.433	0.470	107.85	107.29	11.86	332.46	32.68	22.82	12.25
8	35	3	2	0.494	0.418	0.492	0.426	0.554	123.73	122.79	14.17	332.07	31.95	23.34	11.44
	35	3	2	0.476	0.387	0.495	0.414	0.451	111.20	115.64	11.86	331.27	32.61	21.77	10.82
9	30	2	1	0.471	0.443	0.490	0.435	0.439	123.26	115.67	13.25	333.62	30.62	23.85	12.16
	30	2	1	0.487	0.431	0.486	0.448	0.480	130.89	124.67	13.67	336.14	31.79	22.88	12.12

a Numbers in brackets are assigned values.

b No assigned value for FB_3_.

### Confirmation

Method developer LC–MS/MS data were shown to meet US and EU criteria (17,18). For each analyte, the supplementary data summary in Supplemental Tables 20–26 shows that the matrix ion ratios were within US and/or EU criteria.

Overall, the method was used successfully in the claimed matrixes, meeting Codex performance parameters in most cases and in agreement with the results of the independent laboratory study.

### Column capacity

Finally, the column capacity was determined for all analytes, simultaneously loaded onto the column. Total aflatoxin was determined to be 450 ng, DON 2000 ng, Total fumonisins FB1, FB2 and FB3 (4:2:1) 6000 ng, OTA 1400 ng, total T-2 and HT-2 (1:1) 1050 ng, and ZON 800 ng (Table 11; *see*  Supplemental Figure 7).

**Table 11.** Capacity results

Analyte		AFB_1_	AFB_2_	AFG_1_	AFG_2_	Total AFT	DON	FB_1_	FB_2_	FB_3_	FUM	OTA	T-2	HT-2	T-2 and HT-2	ZON
N		6	6	6	6	6	6	6	6	6	6	6	6	6	6	6
Spike, ng		112.5	112.5	112.5	112.5	450	2000	3429	1714	857	6000	1400	525	525	1050	800
Clear solution	Mean	96.0	79.4	90.0	54.3	79.9	81.8	85.1	76.9	83.9	82.7	84.0	100.1	56.1	78.1	74.0
	SD	1.2	1.5	2.0	2.5	1.1	1.5	2.2	2.6	3.0	1.8	2.1	2.6	2.4	1.2	1.4
	RSD_r_	1.2	1.9	2.2	4.6	1.4	1.9	2.6	2.3	3.5	2.2	2.5	2.6	4.3	1.6	1.9

## Conclusions

The 11^+^Myco MS-PREP immunoaffinity column with LC–MS/MS method was validated under the AOAC *Performance Tested Methods* program, according to AOAC RI policies and procedures. Overall, data from the method developer studies show the criteria for assay calibration, selectivity, product consistency and stability, robustness, and LC–MS/MS confirmation. Using both spiked and certified reference materials, the claimed matrixes of corn, wheat, cereal-based baby food, paprika, chili powder, and animal feed met the criteria set out for acceptable bias, recovery, repeatability, precision, LOD_est_, and LOQ. The independent laboratory study using a subset of the claimed matrixes (corn and animal feed samples) demonstrated the criteria set out for acceptable bias, recovery, repeatability, precision, LOD_est_, and LOQ were met.

The study data have been evaluated in the AOAC RI *Performance Tested Methods* Program and support certification of the 11^+^Myco MS-PREP immunoaffinity column with LC–MS/MS method (AOAC *Performance Tested Method*SM 112401) for the claimed matrixes of corn, wheat, cereal-based baby food, paprika, chili powder, and animal feed.

## Supplemental Information

Supplemental information is available on the *J. AOAC Int*. website.

## Supplementary Material

qsae097_Supplementary_Data
