# Supplementary material for: Validation of the 11+Myco MS-PREP® Method for Determination of Aflatoxins, Fumonisins, Deoxynivalenol, Ochratoxin A, Zearalenone, HT-2, and T-2 Toxins in Cereals, Baby Food, Spices, and Animal Feed by Immunoaffinity Column with LC–MS/MS: AOAC Performance Tested MethodSM 112401
Source: J AOAC Int. 2024 Dec 11;108(2):207–52. doi: 10.1093/jaoacint/qsae097 (PMC11879221; doi:10.1093/jaoacint/qsae097)
Supplement: qsae097_Supplementary_Data [file qsae097_supplementary_data.docx]

**11+ Myco MS-PREP – Supplementary Data**

**Table 1. Combined working standard 1 solution preparation^a^**

| Toxin | Stock Standard concentration (ng/mL) | Volume of Stock Standard Added (µL) | Combined Working Standard Concentration (ng/mL) |
| --- | --- | --- | --- |
| AFLA (1:1:1:1) | 2500  (625 each B_1_, B_2_, G_1_, G_2_) | 240 | 60  (15 each B_1_, B_2_, G_1_, G_2_) |
| Aflatoxin M_1_ | 1,000 | 150 | 15 |
| DON | 100,000 | 375 | 3,750 |
| FUM (4:2:1) | 437,500  (250,000 FB_1_, 125,000 FB_2_, 62,500 FB_3_) | 137 | 6,000  (3,429 FB_1_, 1,714 FB_2_,  857 FB_3_) |
| OTA | 10,000 | 80 | 80 |
| T-2 & HT-2 (1:1) | 100,000  (50,000 each T-2 and HT-2) | 80 | 800  (400 each T-2 and HT-2) |
| ZON | 100,000 | 75 | 750 |
| MeOH |  | 3,863 |  |
| H_2_O |  | q.s. to 10.0 mL |  |

^a^ Adjust volumes as needed depending on stock standard concentrations.

**Table 2. Combined working standard 2 solution preparation (without AFLA M_1_)^a^**

| Toxin | Stock Standard concentration (ng/mL) | Volume of Stock Standard Added (µL) | Combined Working Standard Concentration (ng/mL) |
| --- | --- | --- | --- |
| AFLA (1:1:1:1) | 2500  (625 each B_1_, B_2_, G_1_, G_2_) | 240 | 60  (15 each B_1_, B_2_, G_1_, G_2_) |
| DON | 100,000 | 375 | 3,750 |
| FUM (4:2:1) | 437,500  (250,000 FB_1_, 125,000 FB_2_, 62,500 FB_3_) | 137 | 6,000  (3,429 FB_1_, 1,714 FB_2_,  857 FB_3_) |
| OTA | 10,000 | 80 | 80 |
| T-2 & HT-2 (1:1) | 100,000  (50,000 each T-2 and HT-2) | 80 | 800  (400 each T-2 and HT-2) |
| ZON | 100,000 | 75 | 750 |
| MeOH |  | 4013 |  |
| H_2_O |  | q.s. to 10.0 mL |  |

^a^ Adjust volumes as needed depending on stock standard concentrations.

**Table 4. Toxin concentrations of calibration curve standards**

| Toxin | Concentration of each toxin (ng/mL) | | | | | | |
| --- | --- | --- | --- | --- | --- | --- | --- |
|  | Std 7 | Std 6 | Std 5 | Std 4 | Std 3 | Std 2 | Std 1 |
| Aflatoxin B_1_ | 1.50 | 0.75 | 0.375 | 0.125 | 0.0417 | 0.0139 | 0.00463 |
| Aflatoxin B_2_ | 1.50 | 0.75 | 0.375 | 0.125 | 0.0417 | 0.0139 | 0.00463 |
| Aflatoxin G_1_ | 1.50 | 0.75 | 0.375 | 0.125 | 0.0417 | 0.0139 | 0.00463 |
| Aflatoxin G_2_ | 1.50 | 0.75 | 0.375 | 0.125 | 0.0417 | 0.0139 | 0.00463 |
| AFT^a^ | 6.0 | 3.0 | 1.5 | 0.5 | 0.1667 | 0.0556 | 0.0185 |
| Aflatoxin M_1_^b^ | 1.50 | 0.75 | 0.375 | 0.125 | 0.0417 | 0.0139 | 0.00463 |
| DON | 375 | 187.5 | 93.75 | 31.25 | 10.42 | 3.472 | 1.157 |
| Fumonisin B_1_ | 343.4 | 171.7 | 85.85 | 28.62 | 9.539 | 3.180 | 1.060 |
| Fumonisin B_2_ | 176.2 | 88.12 | 44.06 | 14.69 | 4.896 | 1.632 | 0.5440 |
| Fumonisin B_3_ | 80.35 | 40.17 | 20.09 | 6.695 | 2.232 | 0.7439 | 0.2480 |
| FUM^c^ | 600 | 300 | 150 | 50 | 16.67 | 5.556 | 1.852 |
| OTA | 8.0 | 4.0 | 2.0 | 0.6667 | 0.2222 | 0.0741 | 0.0247 |
| T-2 | 40.0 | 20.0 | 10.0 | 3.333 | 1.111 | 0.3704 | 0.1235 |
| HT-2 | 40.0 | 20.0 | 10.0 | 3.333 | 1.111 | 0.3704 | 0.1235 |
| Total T-2 & HT-2 | 800 | 40.0 | 20.0 | 6.667 | 2.222 | 0.7407 | 0.2469 |
| ZON | 75.0 | 37.5 | 18.75 | 6.25 | 2.083 | 0.6944 | 0.2315 |

^a^Total of Aflatoxins B_1_, B_2_, G_1_, and G_2_.

^b^If included.

^c^Total of Fumonisins B_1_, B2, and B_3_.

**Table 4. MS/MS Conditions**

| **Scheduled MRM Parameters** | |
| --- | --- |
| Mode | Electrospray Ionization |
| Polarity | Positive |
| MRM Window | 60 s |
| Target Cycle Time | 0.35 s |
| Minimum Dwell Time | 10 ms |
| Maximum Dwell Time | 175 ms |
| **Ion Source / Gas Settings** | |
| Curtain gas | 30 |
| IonSpray Voltage | + 4,500 V |
| Turbo Gas Temperature | 500 ºC |
| Ion Source Gas 1 (Nebuliser gas) | 40 |
| Ion Source Gas 2 (Heater gas) | 50 |
| Collision gas | 7 |

**Table 5. sMRM settings**

| Analyte |  | Retention Time (min)^a^ | Presursor Ion (m/z) | Product Ion  Quantifier  Qualifier (m/z) | Declustering Potential | Collison energy (V) | Collision exit potential (V) |
| --- | --- | --- | --- | --- | --- | --- | --- |
| AFB_1_ |  | 3.50 | 313.2  [M+H]+ | 285.1  241.0 | 170 | 30.7  48.2 | 17  17 |
| AFB_2_ |  | 3.34 | 315.2  [M+H]+ | 259.0  203.0 | 170 | 38.6  48.8 | 17  15 |
| AFG_1_ |  | 3.10 | 331.1  [M+H]+ | 243.0  199.9 | 140 | 36.6  53.7 | 15  15 |
| AFG_2_ |  | 2.90 | 329.1  [M+H]+ | 189.0  256.9 | 140 | 54.4  40.9 | 14  20 |
| AFM_1_ |  | 2.90 | 329.1  [M+H]+ | 273.1  229.1 | 170 | 32.1  56.7 | 16  16 |
| DON |  | 1.32 | 297.1  [M+H]+ | 249.0  231.0 | 110 | 15.0  17.0 | 16  15 |
| FB_1_ |  | 3.80 | 722.4  [M+H]+ | 334.3  352.3 | 100 | 76.0  68.0 | 16  20 |
| FB_2_ |  | 4.30 | 706.4  [M+H]+ | 336.2  318.2 | 120 | 71.0  71.0 | 20  20 |
| FB_3_ |  | 4.10 | 706.41  [M+H]+ | 336.2  318.2 | 120 | 61.0  56.0 | 20  20 |
| OTA |  | 4.54 | 404.1  [M+H]+ | 239.0  358.0 | 78 | 31.2  19.1 | 15  18 |
| T-2 |  | 4.27 | 484.3  [M+NH_4_]+ | 245.0  305.1 | 60 | 17.3  17.9 | 16  15 |
| HT-2 |  | 3.90 | 442.3  [M+NH_4_]+ | 263.0  215.0 | 52 | 16.9  17.2 | 15  16 |
| ZON |  | 4.50 | 319.1  [M+H]+ | 283.0  187.1 | 90 | 16.4  25.9 | 17  12 |

^a^ Retention times may vary.

**Table 6. Conversion factors for calculating mycotoxin concentration in matrix from that in the IAC eluant.**

| Parameter | Cereal  (corn, wheat) | Cereal-based baby food | Animal feed | Spices |
| --- | --- | --- | --- | --- |
| Gram equivalents injected (g) | 0.001333 | 0.002667 | 0.000333 | 0.000667 |
| Conversion factor to ppb (ng/g) | 750 | 375 | 3000 | 1500 |

**Table 7. Reference materials for the validation studies**

| Matrix | Toxins and Conc. | Source | Cat. No. |
| --- | --- | --- | --- |
| Corn | AFB_1_: 0.52 ng/g  AFB_2_: 0.41 ng/g  AFG_1:_ 0.5 ng/g  AFG_2_: 0.405ng/g  AFLA: 1.87  DON: 298 ng/g  OTA: 0.52 ng/g  T-2: 20.3 ng/g  HT-2: 16.2 ng/g  Sum T-2 & HT-2: 36.6ng/g  FB_1_: 133 ng/g FB_2_: 133 ng/g  FUM: 253 ng/g ZON: 29.8 ng/g | FAPAS, York, UK  DON, FB1 and FB2 was present in the test material and the levels were elevated with a spiking solution.  Aflatoxin B1, Aflatoxin B2, Aflatoxin G1, Aflatoxin G2, Ochratoxin A, T-2, HT-2 and ZON were spiked into the test material | TCL0406QC |
| Wheat | AFB_1_: 10.6 ng/g  AFLA: 10.6 ng/g  DON: 1300 ng/g  FB_1_: 500 ng/g  FB_2_: 200 ng/g  FB_3_: 100 ng/g  FUM: 800 ng/g  OTA: 22.0 ng/g  T-2: 445.9 ng/g  HT-2: 197.5 ng/g  ZON: 385.9 ng/g | Trilogy Analytical Laboratories, Washington, MO USA  Naturally contaminated | TP-QC-MYC-WHE  (TP-QC-MYC-20-1-WHE) |
| Wheat flour | AFB_1_: 2.60 ng/g  AFB_2_: 1.53 ng/g  AFG_1_: 1.70 ng/g  AFG_2_: 0.900 ng/g  AFLA: 6.69 ng/g  DON: 745 ng/g  T-2: 32.4 ng/g  HT-2: 22.3 ng/g  T-2 + HT-2: 54.0 ng/g  ZON: 74.4 ng/g | FAPAS, York, UK  Naturally contaminated with low levels of DON and ZON, these levels were elevated by spiking. Aflatoxin B1, aflatoxin B2, aflatoxin G1 aflatoxin G2, T-2 and HT-2 were spiked into the test material. | FCMM10-CCP49QC (T22187) |

| Infant Food 1 | AFB_1_: 0.209 ng/g  AFB_2_: 0.145 ng/g  AFG_1_: 0.212 ng/g  AFLA: 0.651 ng/g  OTA: 0.519 ng/g | FAPAS, York, UK  Aflatoxin B1, Aflatoxin B2, Aflatoxin G1, Aflatoxin G2 and Ochratoxin A were spiked into the test material. | FCMM4-INF8QC  (T04424QC) |
| --- | --- | --- | --- |
| Infant Food 2 | AFB_1_: 0.101 ng/g  AFB_2_: 0.089 ng/g  AFLA: 0.183 ng/g  DON: 141 ng/g  OTA: 0.469 ng/g | FAPAS, York, UK  Aflatoxin B1, Aflatoxin B2, Ochratoxin A, Deoxynivalenol (DON) were spiked into the test material. | FCMM6-INF8QC (T04412QC) |
| Animal Feed (Dried Distillers Grains with solubles) | AFB_1_: 17.5 ng/g  AFB_2_: 1.1 ng/g  AFLA: 18.6 ng/g  DON: 2700 ng/g  FB_1_: 8400 ng/g  FB_2_: 3000 ng/g  FB_3_: 800 ng/g  FUM: 12200 ng/g  ZON: 146 ng/g | Trilogy Analytical Laboratories, Washington, MO, USA  Naturally contaminated | Old Product Code: TQC-MMC24-100,  New Product Code: 121229(MM) |
| Pet Dog Food (cereal based) | AFB_1_: 10.9 ng/g  DON: 1090 ng/g  FB_1_: 230 ng/g  FB_2_: 229 ng/g  FUM: 438 ng/g  OTA: 10.0 ng/g  T-2: 52.9 ng/g  HT-2: 56.0 ng/g  T-2 & HT-2: 105 ng/g  ZON: 116 ng/g | FAPAS, York, UK  All analytes were spiked into the test material. | FCMM3-PFO11QC (T04447QC) |
| Chili | AFB_1_: 4.23 ng/g  AFB_2_: 1.73 ng/g  AFG_1_: 3.51 ng/g  AFG_2_: 0.88 ng/g  AFLA: 10.2 ng/g  OTA: 10.2 ng/g | FAPAS, York, UK  Ochratoxin A was naturally contaminated, Aflatoxins were spiked into the material. | FCMM4-SPI6RM (TET016RM) |
| Paprika 1 | AFB_1_: 3.72 ng/g  AFG_1_: 2.4 ng/g | ERM, Joint Research Council, Geel, Belgium  Naturally contaminated | BD286 |

| Paprika 2 | AFB_1_: 4.38 ng/g  AFB_2_: 1.84 ng/g  AFG_1_: 1.87 ng/g  AFG_2_: 0.800 ng/g  AFLA: 8.78 ng/g  OTA: 15.9 ng/g | FAPAS, York, UK  Aflatoxin B2, Aflatoxin G1, Aflatoxin G2 were spiked into the test material. Aflatoxin B1 and Ochratoxin A were naturally contaminated, these levels were elevated by spiking | FCMM4-SPI14QC (T04416QC) |
| --- | --- | --- | --- |

Table 8 Robustness data Aflatoxin B_1_

Table 9 Robustness data Aflatoxin B_2_

Table 10 Robustness data Aflatoxin G_1_

Table 11 Robustness data Aflatoxin G_2_

Table 12 Robustness data OTA

Table 13 Robustness data Fum B_1_

Table 14 Robustness data Fum B_2_

Table 15 Robustness data Fum B_3_

Table 16 Robustness data DON

Table 17 Robustness data ZON

Table 18 Robustness data T-2

Table 19 Robustness data HT-2

**Table 20. LC-MS/MS Confirmation; Corn**

| **Matrix** | **Analyte** | **Component values** | **Sample name** | **Ion ratio** | **Expected ion ratio** | **% difference** |
| --- | --- | --- | --- | --- | --- | --- |
| Corn | AFB_1_ | 285.1 / 241.0 | Cal std 5 standard | 0.7475 | 0.7613 | -1.8127 |
|  |  |  | Cornflour Sample 19 | 0.7538 |  | -0.9852 |
|  | AFB_2_ | 259.0 / 203.0 | Cal std 5 standard | 0.2935 | 0.2886 | 1.6979 |
|  |  |  | Cornflour Sample 19 | 0.2807 |  | -2.7374 |
|  | AFG_1_ | 243.0 / 199.9 | Cal std 5 standard | 0.6108 | 0.6221 | -1.8164 |
|  |  |  | Cornflour Sample 19 | 0.653 |  | 4.9670 |
|  | AFG_2_ | 189.0 / 256.9 | Cal std 5 standard | 0.8127 | 0.8338 | -2.5306 |
|  |  |  | Cornflour Sample 19 | 0.8398 |  | 0.7196 |
|  | OTA | 239.0 / 358.0 | Cal std 5 standard | 0.6165 | 0.6111 | 0.8837 |
|  |  |  | Cornflour Sample 19 | 0.5879 |  | -3.7964 |
|  | Fum B_1_ | 334.3 / 352.3 | Cal std 5 standard | 0.8652 | 0.8584 | 0.7922 |
|  |  |  | Cornflour Sample 19 | 0.85 |  | -0.9786 |
|  | Fum B_2_ | 336.2 / 318.2 | Cal std 5 standard | 0.8716 | 0.8401 | 3.7496 |
|  |  |  | Cornflour Sample 19 | 0.8781 |  | 4.5233 |
|  | Fum B_3_ | 336.2 / 318.2 | Cal std 5 standard | 0.6693 | 0.6839 | -2.1348 |
|  |  |  | Cornflour Sample 19 | 0.6804 |  | -0.5118 |
|  | DON | 249.3 / 231.0 | Cal std 5 standard | 0.5008 | 0.4976 | 0.6431 |
|  |  |  | Cornflour Sample 19 | 0.4997 |  | 0.4220 |
|  | ZON | 283.0 / 187.1 | Cal std 5 standard | 0.5957 | 0.5984 | -0.4512 |
|  |  |  | Cornflour Sample 19 | 0.5942 |  | -0.7019 |
|  | T-2 | 245.0 / 305.1 | Cal std 5 standard | 1.5114 | 1.487 | 1.6409 |
|  |  |  | Cornflour Sample 19 | 1.4973 |  | 0.6927 |
|  | HT-2 | 263.0 / 215.0 | Cal std 5 standard | 0.8861 | 0.9056 | -2.1533 |
|  |  |  | Cornflour Sample 19 | 0.8641 |  | -4.5826 |

**Table 21. LC-MS/MS Confirmation; Wheat**

| **Matrix** | **Analyte** | **Component values** | **Sample name** | **Ion ratio** | **Expected ion ratio** | **% difference** |
| --- | --- | --- | --- | --- | --- | --- |
| Wheat | AFB_1_ | 285.1 / 241.0 | Cal std 5 standard | 0.7476 | 0.7946 | -5.9149 |
|  |  |  | Wheat flour Sample 10 | 0.745 |  | -6.2421 |
|  | AFB_2_ | 259.0 / 203.0 | Cal std 5 standard | 0.288 | 0.2908 | -0.9629 |
|  |  |  | Wheat flour Sample 10 | 0.303 |  | 4.1953 |
|  | AFG_1_ | 243.0 / 199.9 | Cal std 5 standard | 0.6528 | 0.6325 | 3.2095 |
|  |  |  | Wheat flour Sample 10 | 0.6037 |  | -4.5534 |
|  | AFG_2_ | 189.0 / 256.9 | Cal std 5 standard | 0.8251 | 0.8331 | -0.9603 |
|  |  |  | Wheat flour Sample 10 | 0.8014 |  | -3.8051 |
|  | OTA | 239.0 / 358.0 | Cal std 5 standard | 0.6288 | 0.6095 | 3.1665 |
|  |  |  | Wheat flour Sample 10 | 0.6455 |  | 5.9065 |
|  | Fum B_1_ | 334.3 / 352.3 | Cal std 5 standard | 0.8334 | 0.859 | -2.9802 |
|  |  |  | Wheat flour Sample 10 | 0.8241 |  | -4.0629 |
|  | Fum B_2_ | 336.2 / 318.2 | Cal std 5 standard | 0.8394 | 0.8552 | -1.8475 |
|  |  |  | Wheat flour Sample 10 | 0.8263 |  | -3.3793 |
|  | Fum B_3_ | 336.2 / 318.2 | Cal std 5 standard | 0.6509 | 0.6593 | -1.2741 |
|  |  |  | Wheat flour Sample 10 | 0.6955 |  | 5.4907 |
|  | DON | 249.3 / 231.0 | Cal std 5 standard | 0.5023 | 0.4951 | 1.4543 |
|  |  |  | Wheat flour Sample 10 | 0.4967 |  | 0.3232 |
|  | ZON | 283.0 / 187.1 | Cal std 5 standard | 0.5933 | 0.5914 | 0.3213 |
|  |  |  | Wheat flour Sample 10 | 0.5864 |  | -0.8455 |
|  | T-2 | 245.0 / 305.1 | Cal std 5 standard | 1.5526 | 1.4594 | 6.3862 |
|  |  |  | Wheat flour Sample 10 | 1.4924 |  | 2.2612 |
|  | HT-2 | 263.0 / 215.0 | Cal std 5 standard | 0.9368 | 0.8987 | 4.2395 |
|  |  |  | Wheat flour Sample 10 | 0.8208 |  | -8.6681 |

**Table 22. LC-MS/MS Confirmation; Non-Dairy Baby Food**

| **Matrix** | **Analyte** | **Component values** | **Sample name** | **Ion ratio** | **Expected ion ratio** | **% difference** |
| --- | --- | --- | --- | --- | --- | --- |
| Baby food non dairy | AFB_1_ | 285.1 / 241.0 | Cal std 5 standard | 0.7636 | 0.7423 | 2.8695 |
|  |  |  | Baby Food non dairy  Sample 03 | 0.6864 |  | -7.5306 |
|  | AFB_2_ | 259.0 / 203.0 | Cal std 5 standard | 0.2752 | 0.2919 | -5.7211 |
|  |  |  | Baby Food non dairy  Sample 03 | 0.3182 |  | 9.0099 |
|  | AFG_1_ | 243.0 / 199.9 | Cal std 5 standard | 0.6181 | 0.6052 | 2.1315 |
|  |  |  | Baby Food non dairy  Sample 03 | 0.634 |  | 4.7588 |
|  | AFG_2_ | 189.0 / 256.9 | Cal std 5 standard | 0.8677 | 0.8335 | 4.1032 |
|  |  |  | Baby Food non dairy  Sample 03 | 0.8468 |  | 1.5957 |
|  | OTA | 239.0 / 358.0 | Cal std 5 standard | 0.6313 | 0.6258 | 0.8789 |
|  |  |  | Baby Food non dairy  Sample 03 | 0.7295 |  | 16.5708 |
|  | Fum B_1_ | 334.3 / 352.3 | Cal std 5 standard | 0.8813 | 0.8665 | 1.7080 |
|  |  |  | Baby Food non dairy  Sample 03 | 0.8964 |  | 3.4507 |
|  | Fum B_2_ | 336.2 / 318.2 | Cal std 5 standard | 0.8738 | 0.87 | 0.4368 |
|  |  |  | Baby Food non dairy  Sample 03 | 0.9348 |  | 7.4483 |
|  | Fum B_3_ | 336.2 / 318.2 | Cal std 5 standard | 0.6811 | 0.6393 | 6.5384 |
|  |  |  | Baby Food non dairy  Sample 03 | 0.6486 |  | 1.4547 |
|  | DON | 249.3 / 231.0 | Cal std 5 standard | 0.4933 | 0.4972 | -0.7844 |
|  |  |  | Baby Food non dairy  Sample 03 | 0.4882 |  | -1.8101 |
|  | ZON | 283.0 / 187.1 | Cal std 5 standard | 0.5983 | 0.5857 | 2.1513 |
|  |  |  | Baby Food non dairy  Sample 03 | 0.585 |  | -0.1195 |
|  | T-2 | 245.0 / 305.1 | Cal std 5 standard | 1.4521 | 1.4808 | -1.9381 |
|  |  |  | Baby Food non dairy  Sample 03 | 1.4643 |  | -1.1143 |
|  | HT-2 | 263.0 / 215.0 | Cal std 5 standard | 0.8789 | 0.9256 | -5.0454 |
|  |  |  | Baby Food non dairy  Sample 03 | 0.9196 |  | -0.6482 |

**Table 23. LC-MS/MS Confirmation; Dairy Baby Food**

| **Matrix** | **Analyte** | **Component values** | **Sample name** | **Ion ratio** | **Expected ion ratio** | **% difference** |
| --- | --- | --- | --- | --- | --- | --- |
| Baby food | AFB_1_ | 285.1 / 241.0 | Cal std 5 standard | 0.8163 | 0.8052 | 1.3785 |
|  |  |  | Baby Food Sample 10 | 0.8463 |  | 5.1043 |
|  | AFB_2_ | 259.0 / 203.0 | Cal std 5 standard | 0.2657 | 0.2904 | -8.5055 |
|  |  |  | Baby Food Sample 10 | 0.2957 |  | 1.8251 |
|  | AFG_1_ | 243.0 / 199.9 | Cal std 5 standard | 0.5658 | 0.575 | -1.6000 |
|  |  |  | Baby Food Sample 10 | 0.505 |  | -12.1739 |
|  | AFG_2_ | 189.0 / 256.9 | Cal std 5 standard | 0.8459 | 0.8266 | 2.3349 |
|  |  |  | Baby Food Sample 10 | 0.7299 |  | -11.6985 |
|  | AFM_1_ | 273.1 / 229.1 | Cal std 5 standard | 0.4158 | 0.4067 | 2.2375 |
|  |  |  | Baby Food Sample 10 | 0.3972 |  | -2.3359 |
|  | OTA | 239.0 / 358.0 | Cal std 5 standard | 0.6681 | 0.6719 | -0.5656 |
|  |  |  | Baby Food Sample 10 | 0.6813 |  | 1.3990 |
|  | Fum B_1_ | 334.3 / 352.3 | Cal std 5 standard | 0.894 | 0.8857 | 0.9371 |
|  |  |  | Baby Food Sample 10 | 0.8811 |  | -0.5194 |
|  | Fum B_2_ | 336.2 / 318.2 | Cal std 5 standard | 0.8333 | 0.8235 | 1.1900 |
|  |  |  | Baby Food Sample 10 | 0.8356 |  | 1.4693 |
|  | Fum B_3_ | 336.2 / 318.2 | Cal std 5 standard | 0.6341 | 0.6328 | 0.2054 |
|  |  |  | Baby Food Sample 10 | 0.6399 |  | 1.1220 |
|  | DON | 249.3 / 231.0 | Cal std 5 standard | 0.4932 | 0.4902 | 0.6120 |
|  |  |  | Baby Food Sample 10 | 0.486 |  | -0.8568 |
|  | ZON | 283.0 / 187.1 | Cal std 5 standard | 0.6277 | 0.6208 | 1.1115 |
|  |  |  | Baby Food Sample 10 | 0.6569 |  | 5.8151 |
|  | T-2 | 245.0 / 305.1 | Cal std 5 standard | 1.4002 | 1.4458 | -3.1540 |
|  |  |  | Baby Food Sample 10 | 1.4158 |  | -2.0750 |
|  | HT-2 | 263.0 / 215.0 | Cal std 5 standard | 0.8783 | 0.9049 | -2.9396 |
|  |  |  | Baby Food Sample 10 | 0.8604 |  | -4.9177 |

**Table 24. LC-MS/MS Confirmation; Animal Feed**

| **Matrix** | **Analyte** | **Component values** | **Sample name** | **Ion ratio** | **Expected ion ratio** | **% difference** |
| --- | --- | --- | --- | --- | --- | --- |
| Animal feed | AFB_1_ | 285.1 / 241.0 | Cal std 5 standard | 0.7799 | 0.7846 | -0.5990 |
|  |  |  | Animal feed Sample 14 | 0.7829 |  | -0.2167 |
|  | AFB_2_ | 259.0 / 203.0 | Cal std 5 standard | 0.2835 | 0.2932 | -3.3083 |
|  |  |  | Animal feed Sample 14 | 0.2744 |  | -6.4120 |
|  | AFG_1_ | 243.0 / 199.9 | Cal std 5 standard | 0.6475 | 0.6404 | 1.1087 |
|  |  |  | Animal feed Sample 14 | 0.6476 |  | 1.1243 |
|  | AFG_2_ | 189.0 / 256.9 | Cal std 5 standard | 0.8361 | 0.8179 | 2.2252 |
|  |  |  | Animal feed Sample 14 | 0.8304 |  | 1.5283 |
|  | OTA | 239.0 / 358.0 | Cal std 5 standard | 0.6354 | 0.618 | 2.8155 |
|  |  |  | Animal feed Sample 14 | 0.6112 |  | -1.1003 |
|  | Fum B_1_ | 334.3 / 352.3 | Cal std 5 standard | 0.8544 | 0.8482 | 0.7310 |
|  |  |  | Animal feed Sample 14 | 0.8338 |  | -1.6977 |
|  | Fum B_2_ | 336.2 / 318.2 | Cal std 5 standard | 0.923 | 0.8238 | 12.0418 |
|  |  |  | Animal feed Sample 14 | 0.8445 |  | 2.5127 |
|  | Fum B_3_ | 336.2 / 318.2 | Cal std 5 standard | 0.6708 | 0.6604 | 1.5748 |
|  |  |  | Animal feed Sample 14 | 0.6675 |  | 1.0751 |
|  | DON | 249.3 / 231.0 | Cal std 5 standard | 0.5007 | 0.4991 | 0.3206 |
|  |  |  | Animal feed Sample 14 | 0.5001 |  | 0.2004 |
|  | ZON | 283.0 / 187.1 | Cal std 5 standard | 0.6124 | 0.6118 | 0.0981 |
|  |  |  | Animal feed Sample 14 | 0.6055 |  | -1.0297 |
|  | T-2 | 245.0 / 305.1 | Cal std 5 standard | 1.5196 | 1.4817 | 2.5579 |
|  |  |  | Animal feed Sample 14 | 1.537 |  | 3.7322 |
|  | HT-2 | 263.0 / 215.0 | Cal std 5 standard | 0.8873 | 0.887 | 0.0338 |
|  |  |  | Animal feed Sample 14 | 0.8724 |  | -1.6460 |

**Table 25. LC-MS/MS Confirmation; Paprika**

| **Matrix** | **Analyte** | **Component values** | **Sample name** | **Ion ratio** | **Expected ion ratio** | **% difference** |
| --- | --- | --- | --- | --- | --- | --- |
| Paprika | AFB_1_ | 285.1 / 241.0 | Cal std 5 standard | 0.7511 | 0.7626 | -1.5080 |
|  |  |  | Paprika Sample 21 | 0.7482 |  | -1.8883 |
|  | AFB_2_ | 259.0 / 203.0 | Cal std 5 standard | 0.2881 | 0.287 | 0.3833 |
|  |  |  | Paprika Sample 21 | 0.2815 |  | -1.9164 |
|  | AFG_1_ | 243.0 / 199.9 | Cal std 5 standard | 0.5989 | 0.6114 | -2.0445 |
|  |  |  | Paprika Sample 21 | 0.6304 |  | 3.1076 |
|  | AFG_2_ | 189.0 / 256.9 | Cal std 5 standard | 0.8163 | 0.8267 | -1.2580 |
|  |  |  | Paprika Sample 21 | 0.7989 |  | -3.3628 |
|  | OTA | 239.0 / 358.0 | Cal std 5 standard | 0.635 | 0.6295 | 0.8737 |
|  |  |  | Paprika Sample 21 | 0.617 |  | -1.9857 |
|  | Fum B_1_ | 334.3 / 352.3 | Cal std 5 standard | 0.8367 | 0.8631 | -3.0587 |
|  |  |  | Paprika Sample 21 | 0.8751 |  | 1.3903 |
|  | Fum B_2_ | 336.2 / 318.2 | Cal std 5 standard | 0.8787 | 0.8623 | 1.9019 |
|  |  |  | Paprika Sample 21 | 0.7316 |  | -15.1571 |
|  | Fum B_3_ | 336.2 / 318.2 | Cal std 5 standard | 0.6601 | 0.6561 | 0.6097 |
|  |  |  | Paprika Sample 21 | 0.6598 |  | 0.5639 |
|  | DON | 249.3 / 231.0 | Cal std 5 standard | 0.5048 | 0.4978 | 1.4062 |
|  |  |  | Paprika Sample 21 | 0.4968 |  | -0.2009 |
|  | ZON | 283.0 / 187.1 | Cal std 5 standard | 0.5828 | 0.5977 | -2.4929 |
|  |  |  | Paprika Sample 21 | 0.6059 |  | 1.3719 |
|  | T-2 | 245.0 / 305.1 | Cal std 5 standard | 1.4568 | 1.467 | -0.6953 |
|  |  |  | Paprika Sample 21 | 1.5944 |  | 8.6844 |
|  | HT-2 | 263.0 / 215.0 | Cal std 5 standard | 0.901 | 0.9167 | -1.7127 |
|  |  |  | Paprika Sample 21 | 0.9728 |  | 6.1198 |

**Table 26. LC-MS/MS Confirmation; Chili**

| **Matrix** | **Analyte** | **Component values** | **Sample name** | **Ion ratio** | **Expected ion ratio** | **% difference** |
| --- | --- | --- | --- | --- | --- | --- |
| Chili | AFB1 | 285.1 / 241.0 | Cal std 5 standard | 0.7876 | 0.8075 | -2.4644 |
|  |  |  | Chili Sample 7 | 0.7881 |  | -2.4025 |
|  | AFB2 | 259.0 / 203.0 | Cal std 5 standard | 0.2747 | 0.2884 | -4.7503 |
|  |  |  | Chili Sample 7 | 0.2858 |  | -0.9015 |
|  | AFG1 | 243.0 / 199.9 | Cal std 5 standard | 0.5772 | 0.5715 | 0.9974 |
|  |  |  | Chili Sample 7 | 0.5865 |  | 2.6247 |
|  | AFG2 | 189.0 / 256.9 | Cal std 5 standard | 0.8184 | 0.8237 | -0.6434 |
|  |  |  | Chili Sample 7 | 0.8516 |  | 3.3872 |
|  | OTA | 239.0 / 358.0 | Cal std 5 standard | 0.6689 | 0.6394 | 4.6137 |
|  |  |  | Chili Sample 7 | 0.6803 |  | 6.3966 |
|  | Fum B1 | 334.3 / 352.3 | Cal std 5 standard | 0.9167 | 0.9152 | 0.1639 |
|  |  |  | Chili Sample 7 | 0.8862 |  | -3.1687 |
|  | Fum B2 | 336.2 / 318.2 | Cal std 5 standard | 0.8696 | 0.849 | 2.4264 |
|  |  |  | Chili Sample 7 | 0.9637 |  | 13.5100 |
|  | Fum B3 | 336.2 / 318.2 | Cal std 5 standard | 0.6099 | 0.6395 | -4.6286 |
|  |  |  | Chili Sample 7 | 0.6219 |  | -2.7522 |
|  | DON | 249.3 / 231.0 | Cal std 5 standard | 0.4985 | 0.4914 | 1.4449 |
|  |  |  | Chili Sample 7 | 0.4936 |  | 0.4477 |
|  | ZON | 283.0 / 187.1 | Cal std 5 standard | 0.6366 | 0.6181 | 2.9930 |
|  |  |  | Chili Sample 7 | 0.6398 |  | 3.5108 |
|  | T-2 | 245.0 / 305.1 | Cal std 5 standard | 1.438 | 1.4114 | 1.8847 |
|  |  |  | Chili Sample 7 | 1.4843 |  | 5.1651 |
|  | HT-2 | 263.0 / 215.0 | Cal std 5 standard | 0.9029 | 0.9084 | -0.6055 |
|  |  |  | Chili Sample 7 | 0.981 |  | 7.9921 |

**Fig 1 Pareto Chart of the Standardized Effects**

A


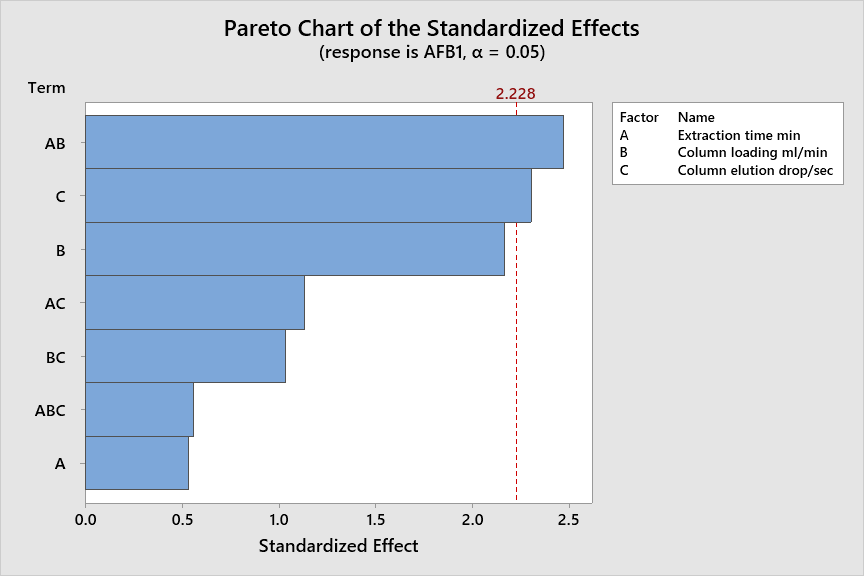


B


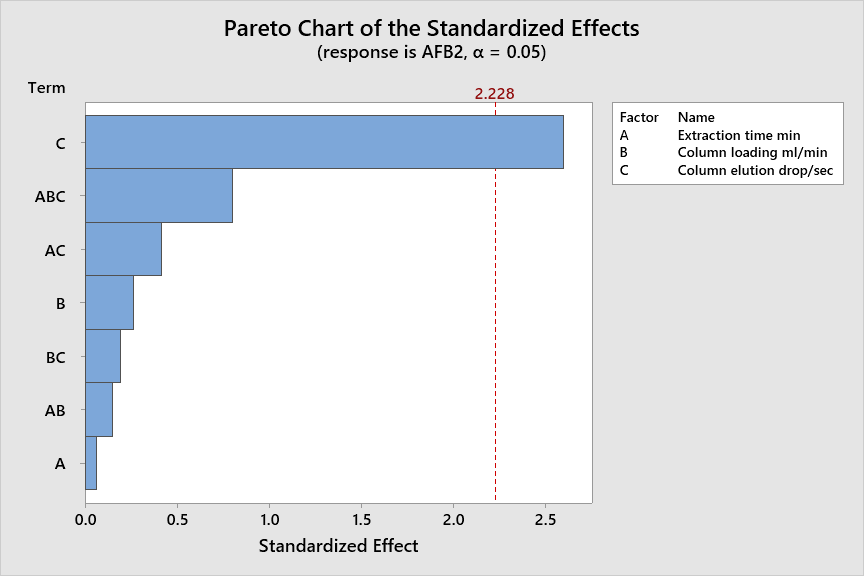


C


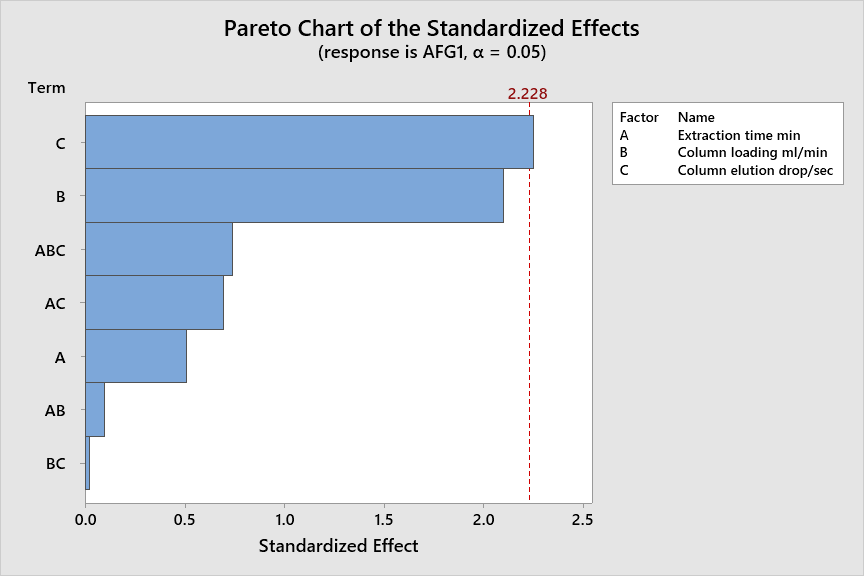


D


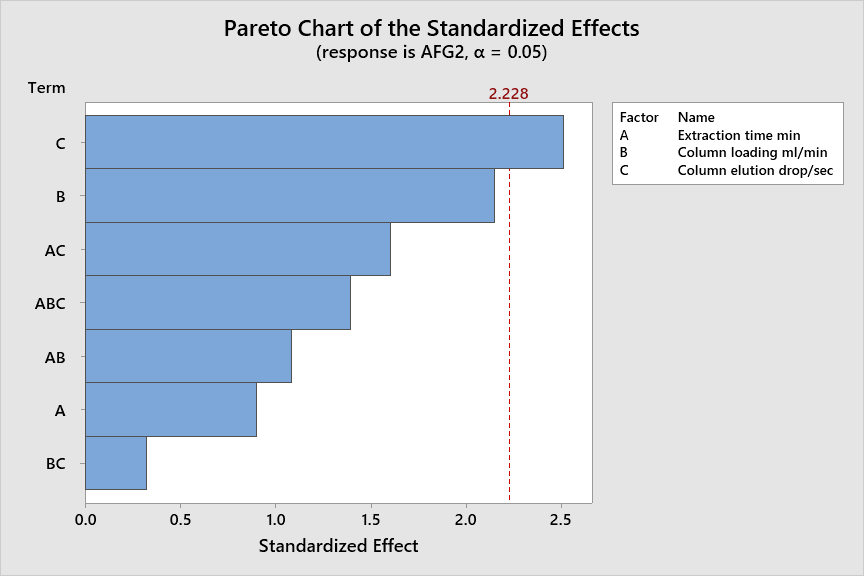


E


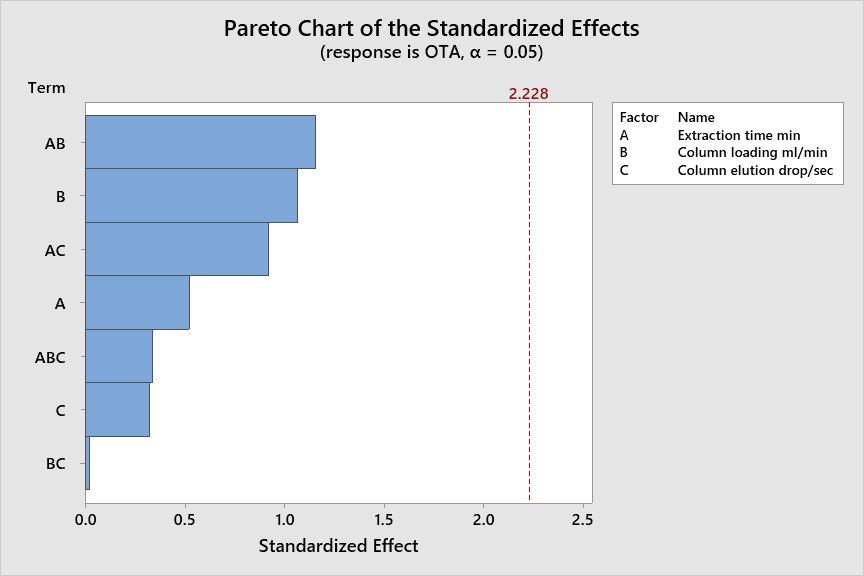


F


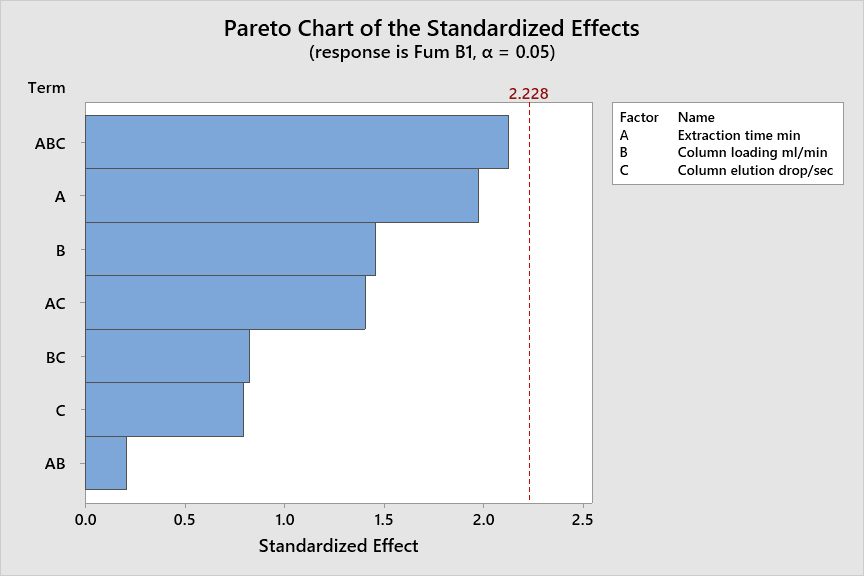


G


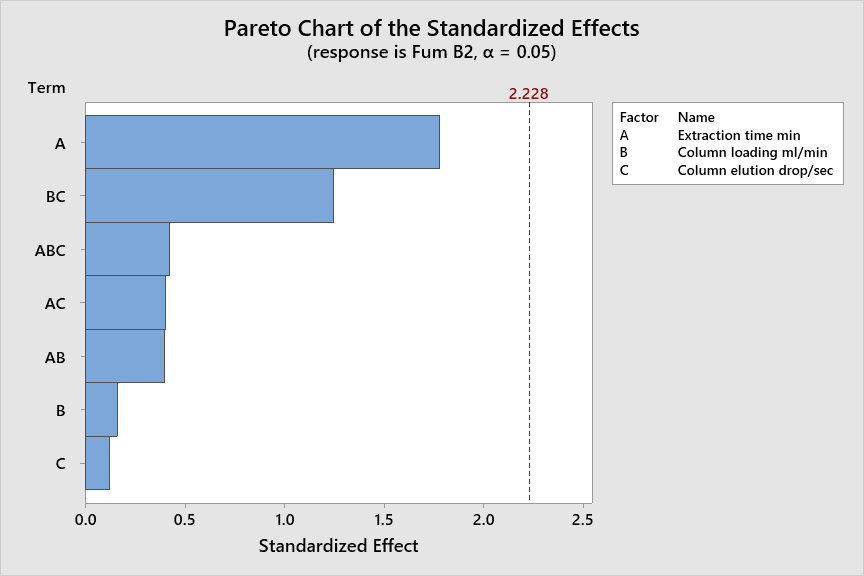


H


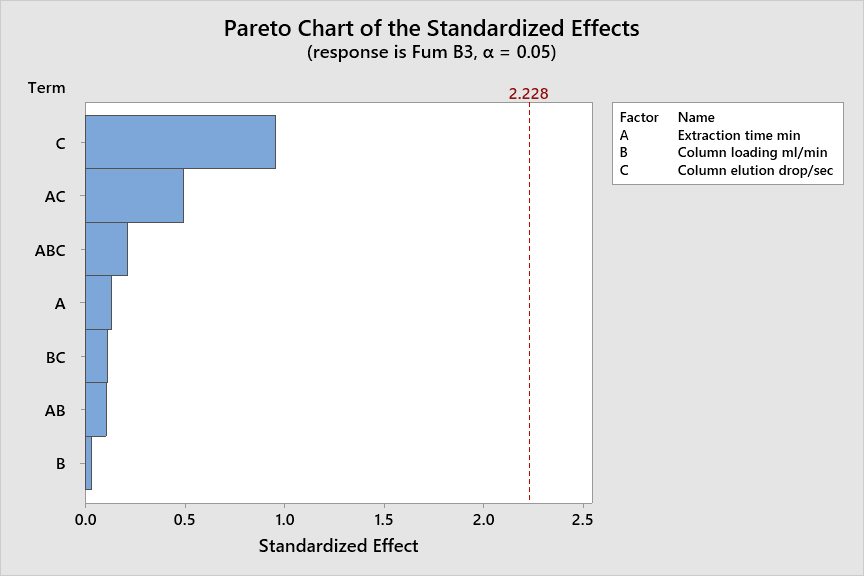


I


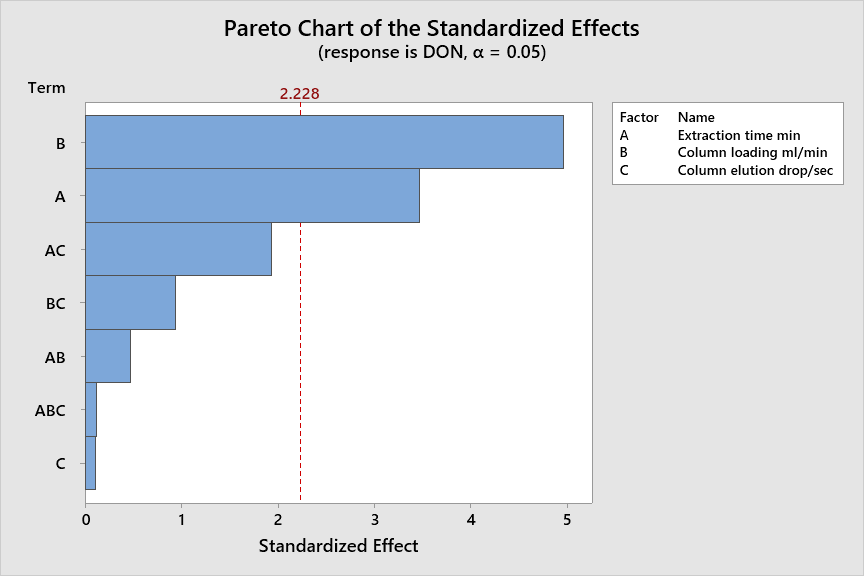


J


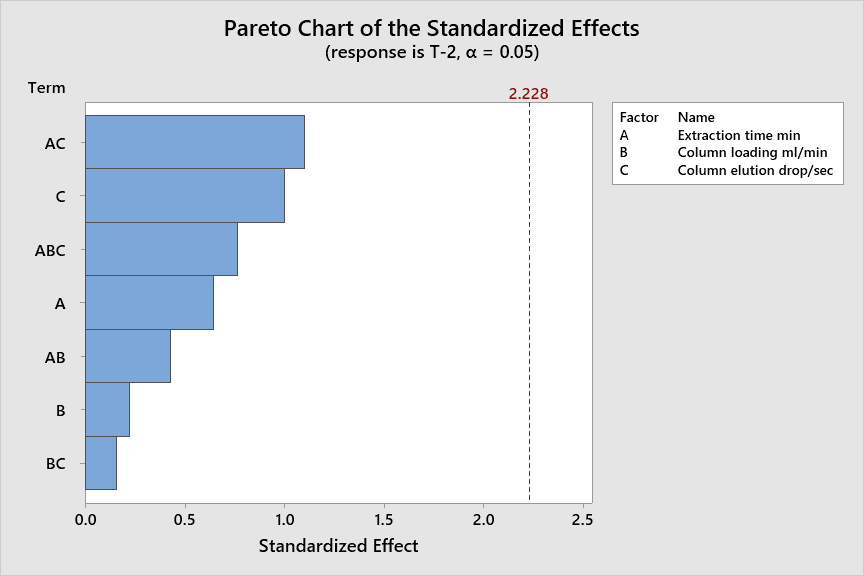


K


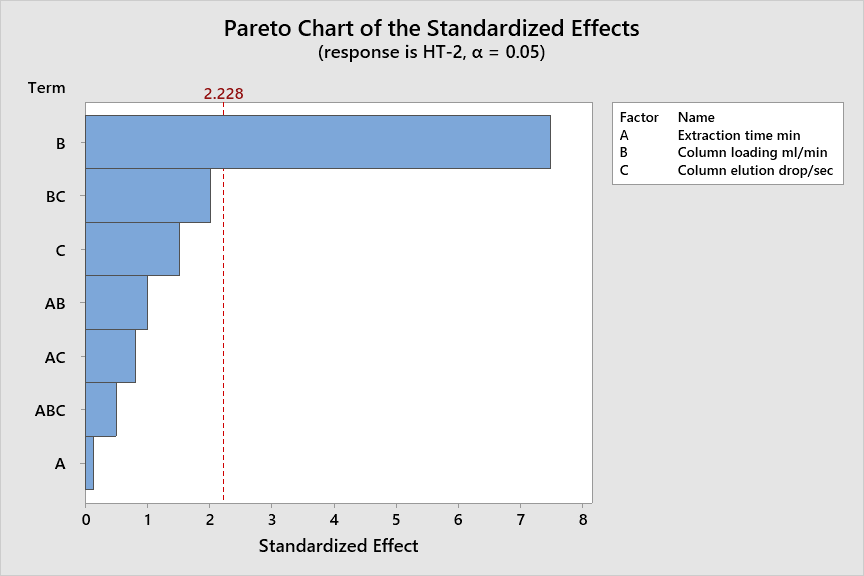


L


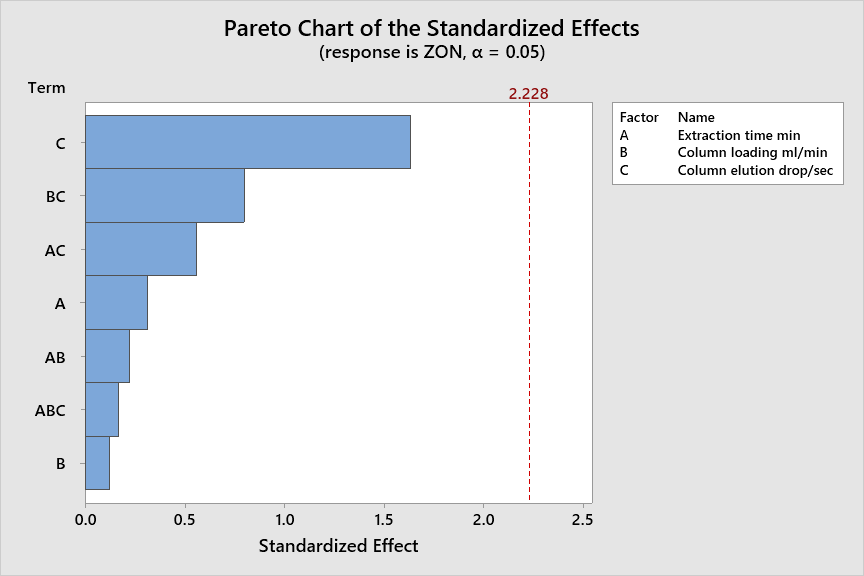


**Figure 2 Selectivity data**

A Selectivity Aflatoxin B_1_


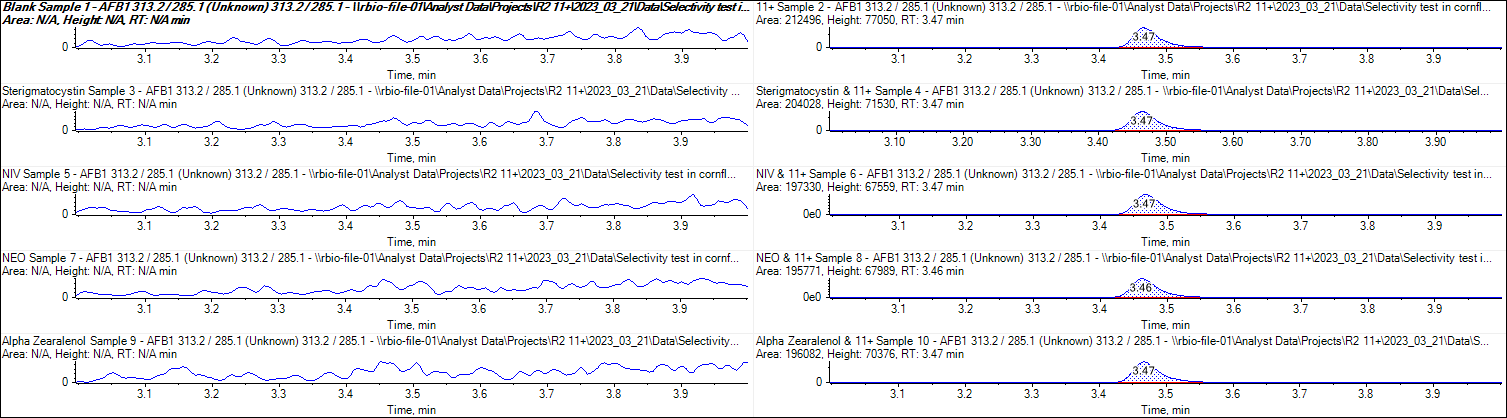


Component value 285.1


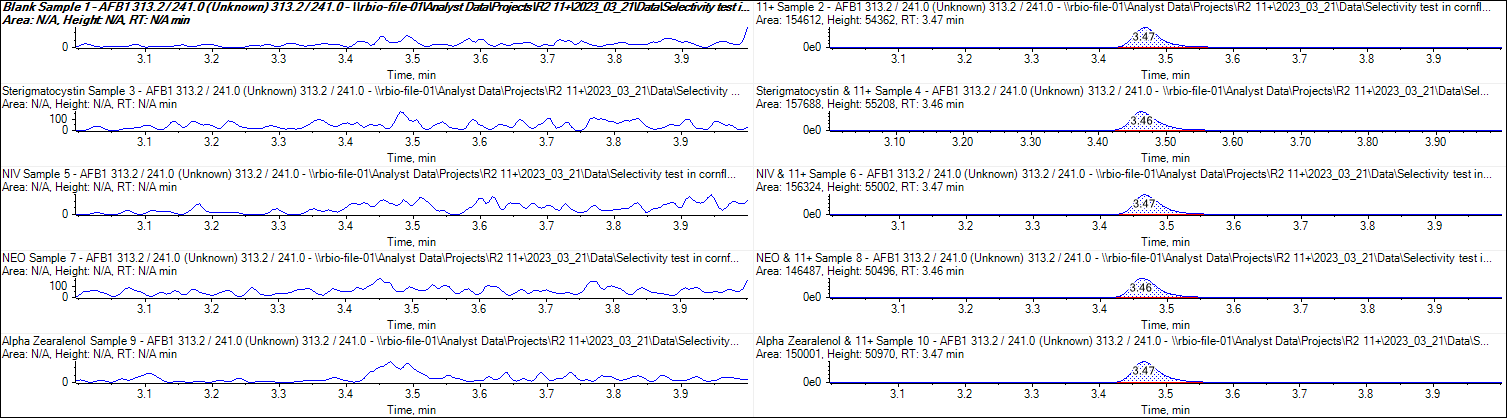


Component value 241.0

B Selectivity Aflatoxin B_2_


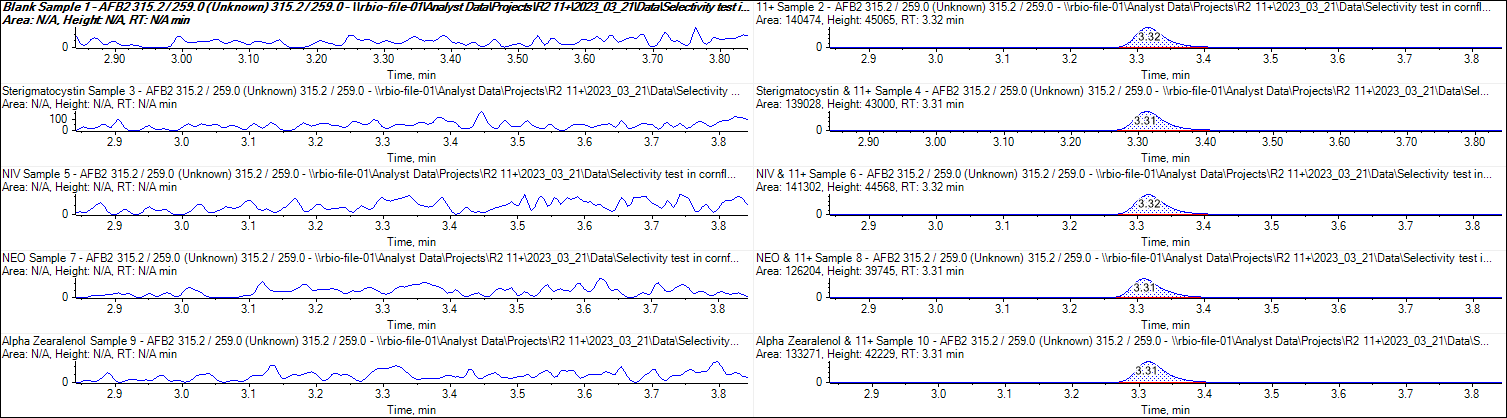


Component value 259.0


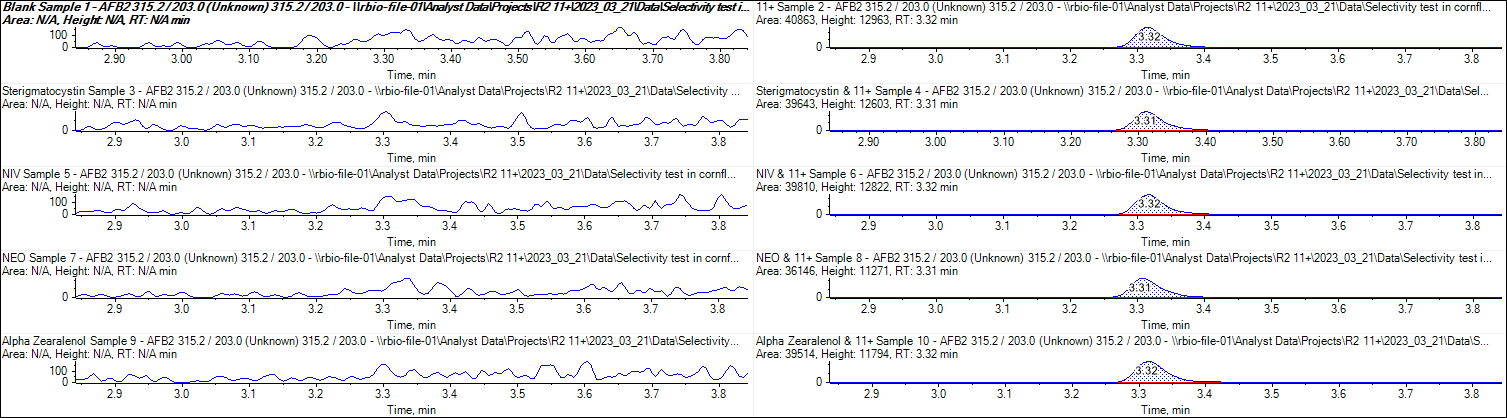


Component value 203.0

C Selectivity Aflatoxin G_1_


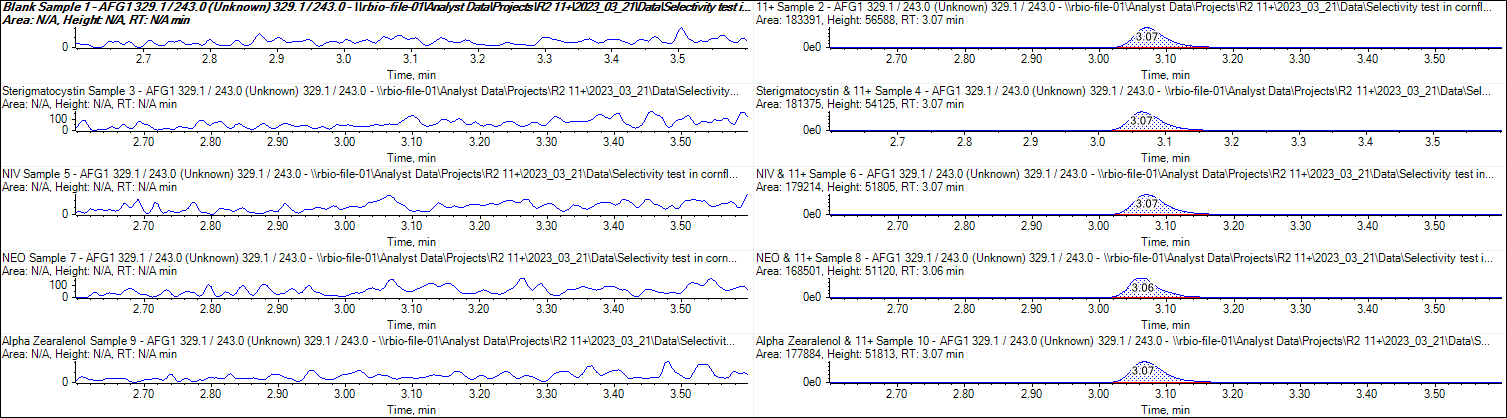


Component value 243.0


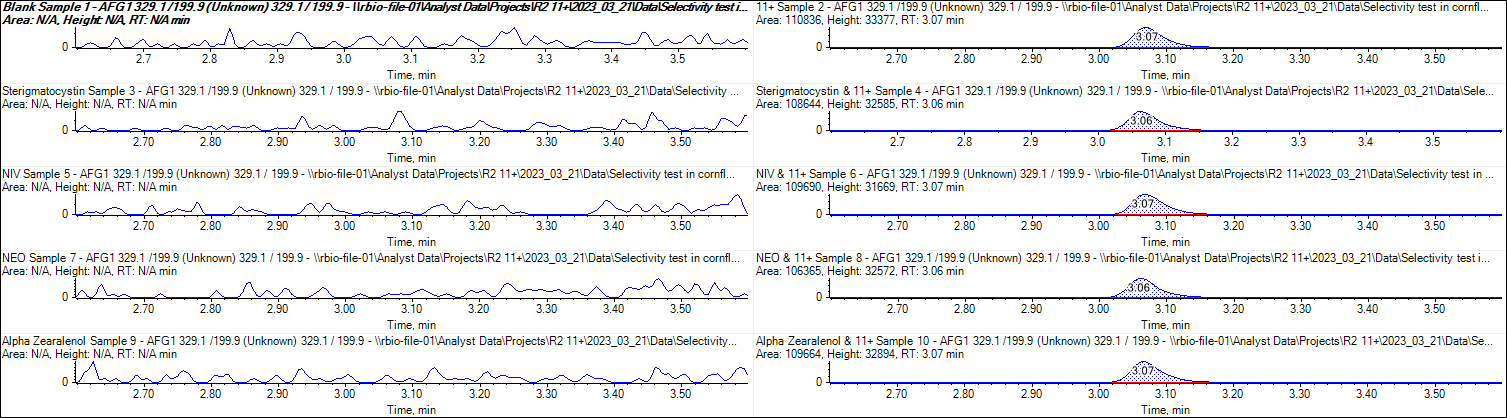


Component value 199.9

D Selectivity Aflatoxin G_2_


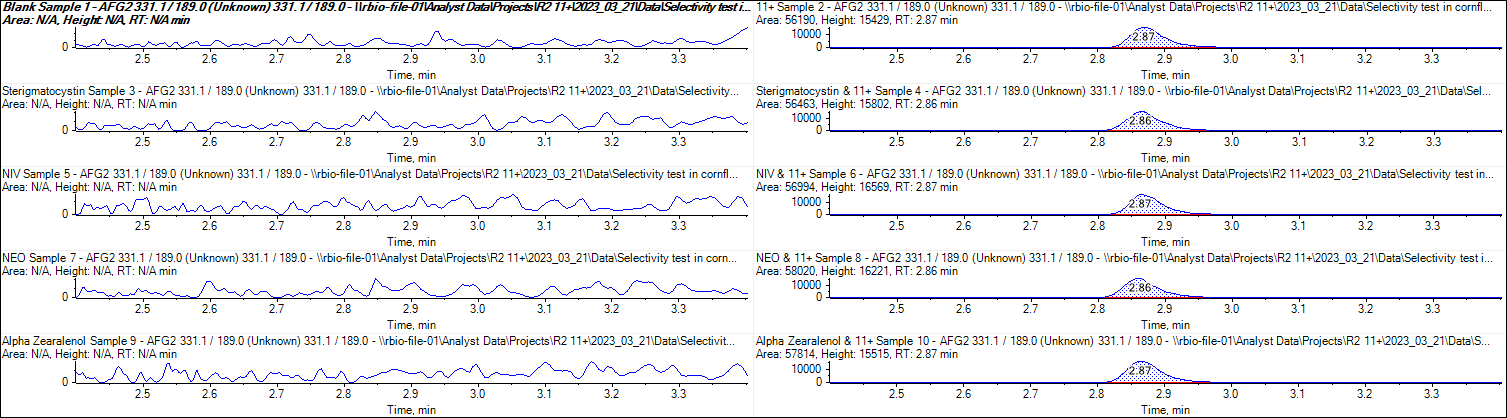


Component value 189.0


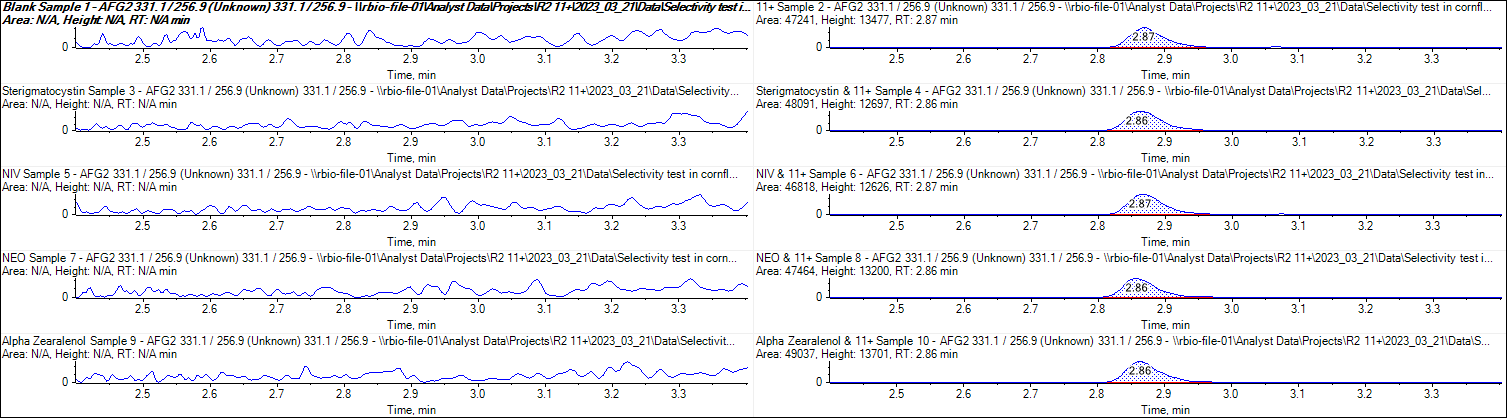


Component value 256.9

E Selectivity Ochratoxin A


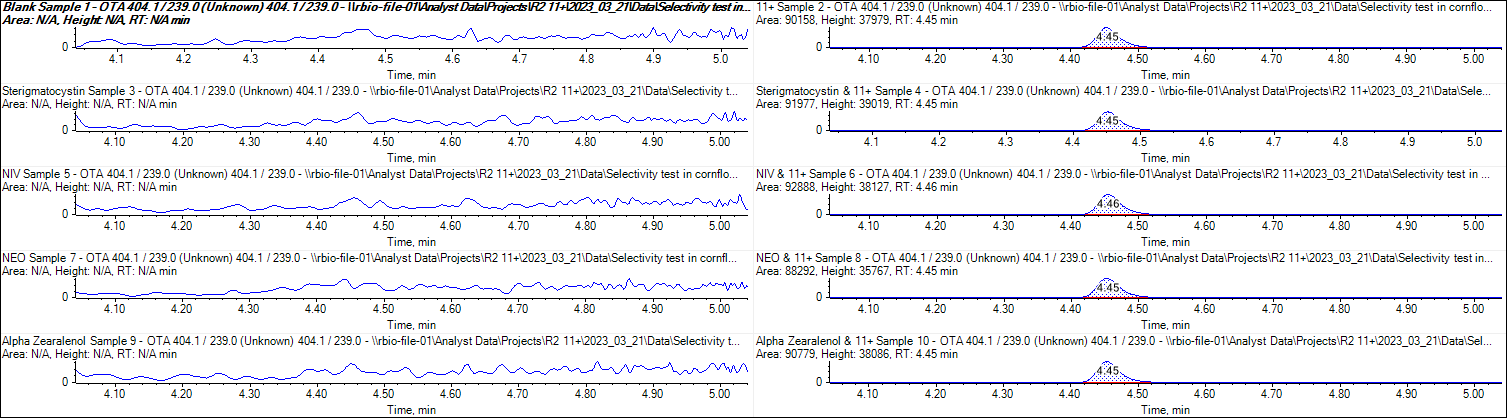


Component value 239.0


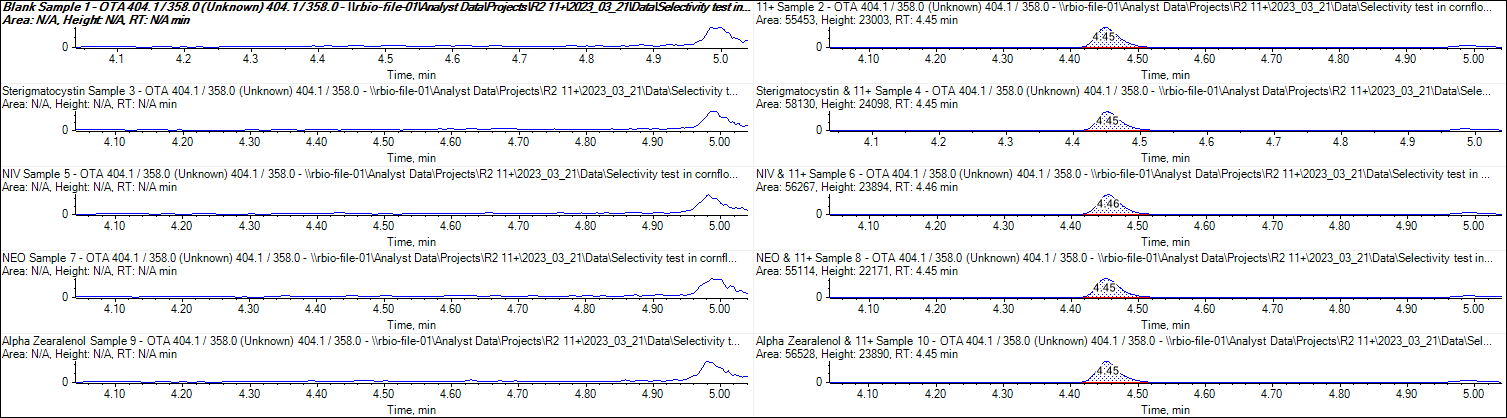


Component value 358.0

F Selectivity Fumonisin B_1_


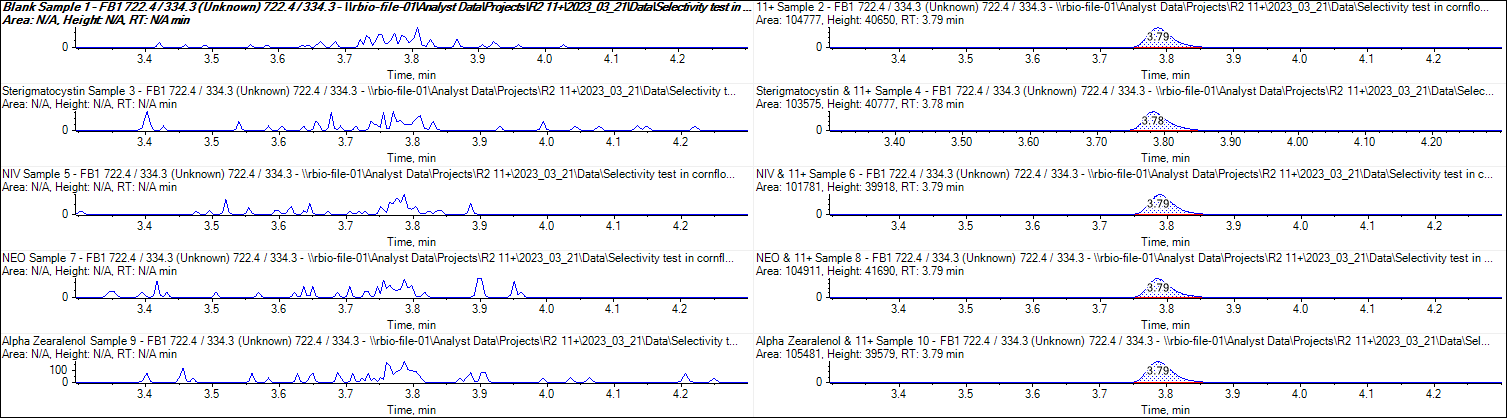


Component value 334.3


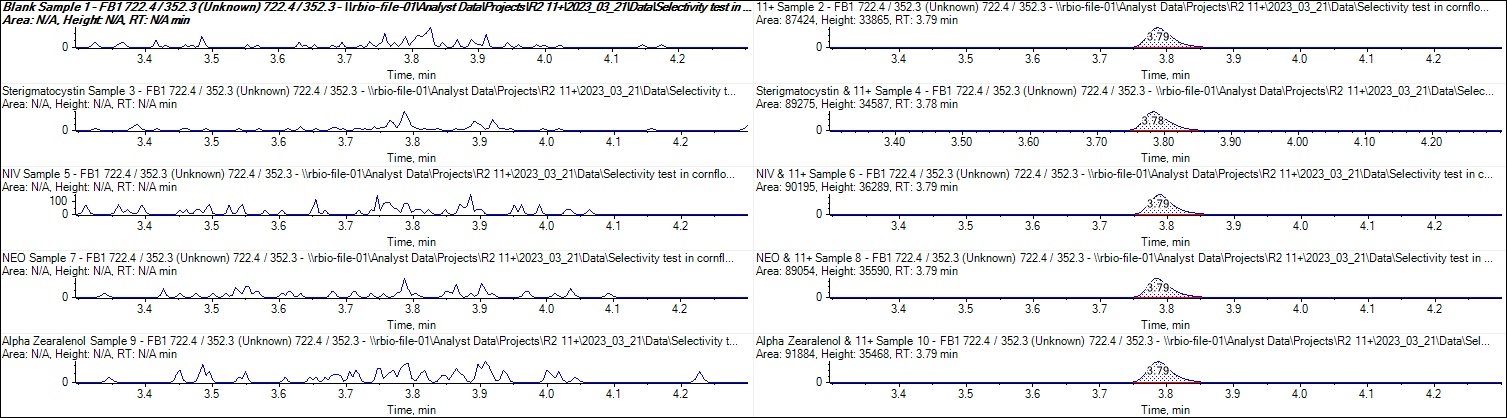


Component value 352.3

G Selectivity Fumonisin B_2_


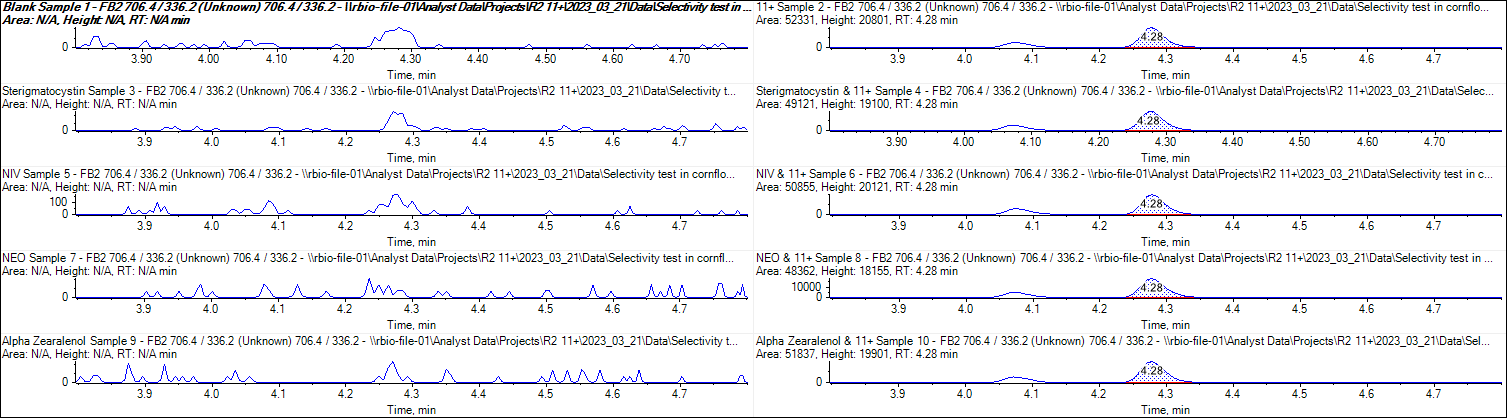


Component value 336.2


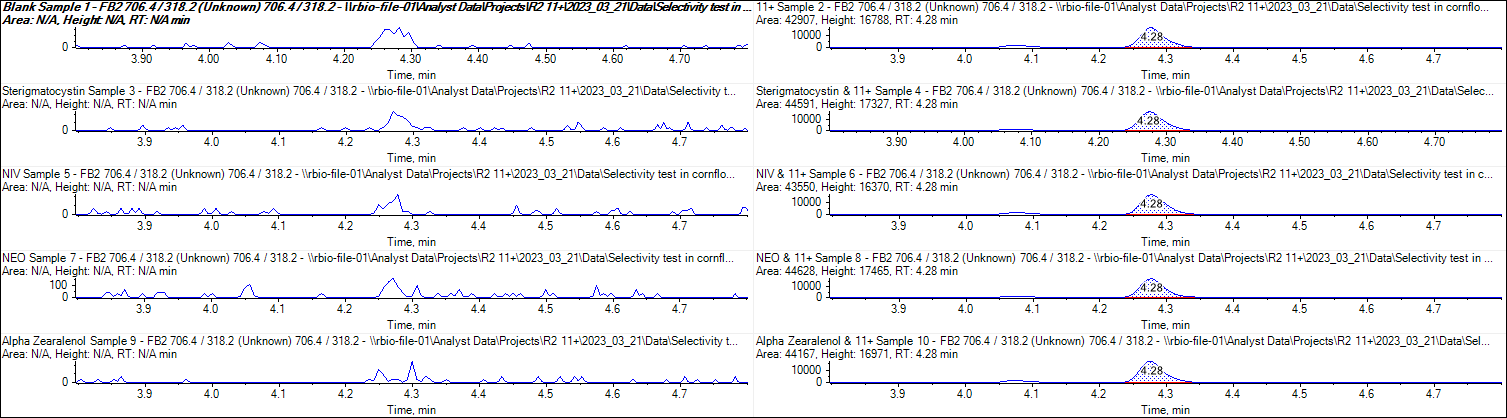


Component value 318.2

H Selectivity Fumonisin B_3_


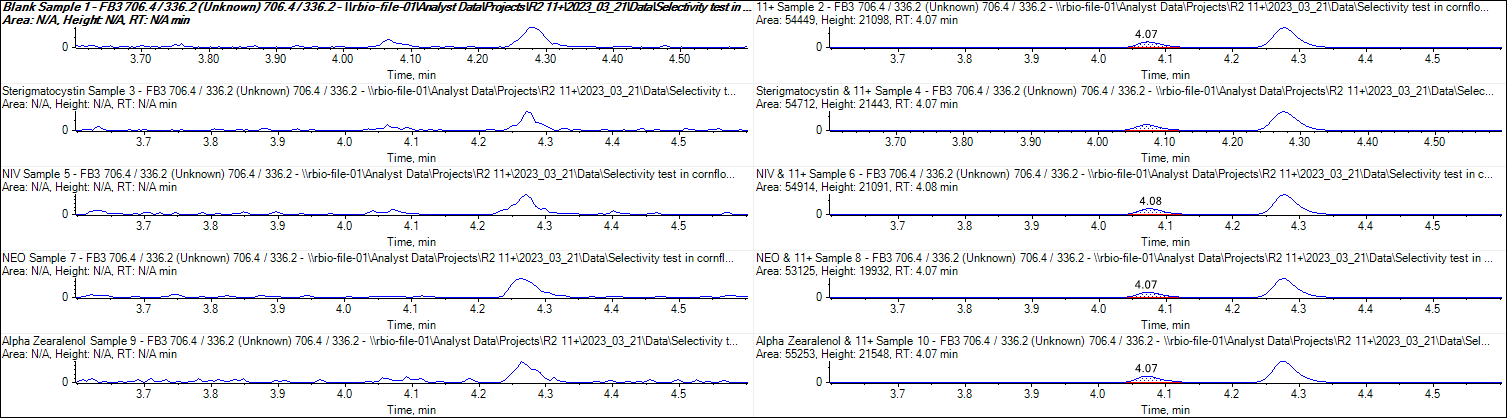


Component value 336.2


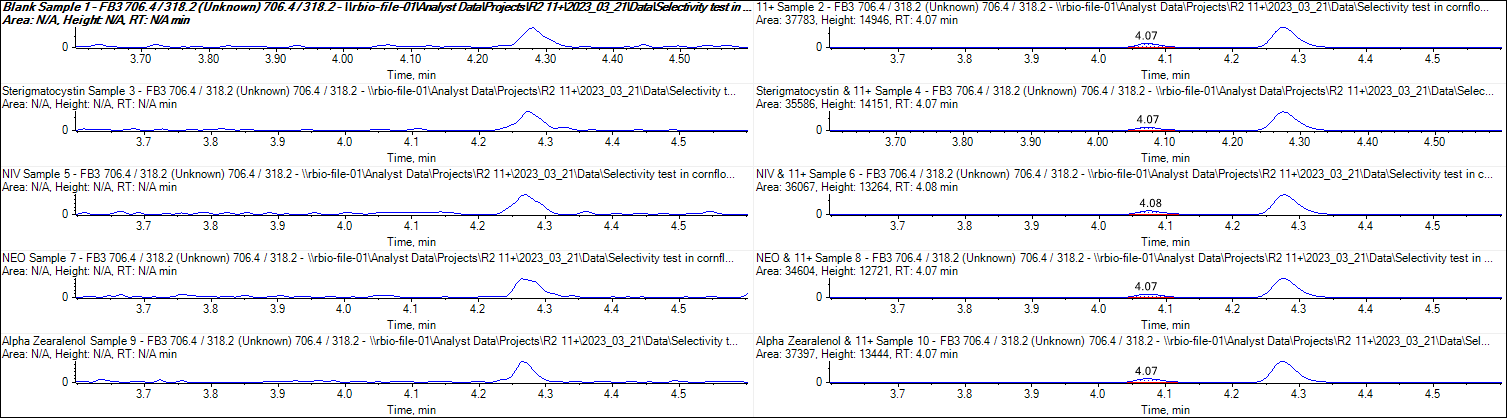


Component value 318.2

I Deoxynivalenol


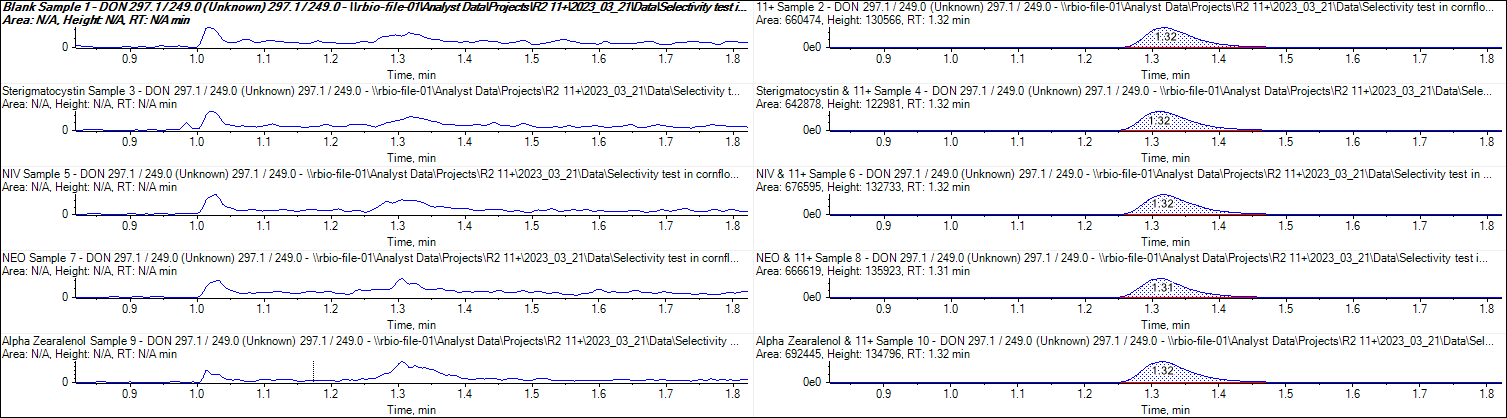


Component value 249.0


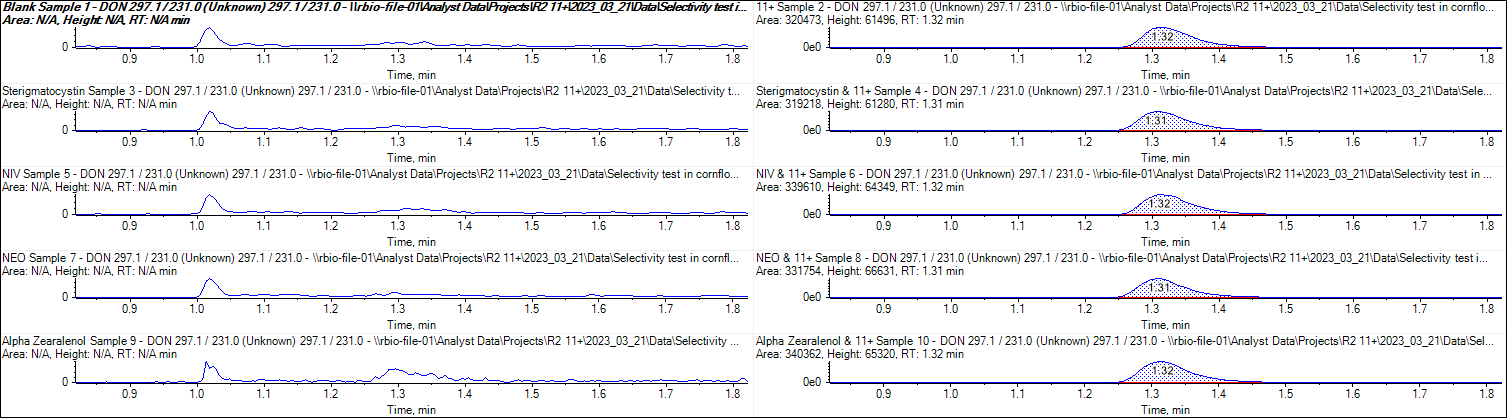


Component value 231.0

J Selectivity T-2


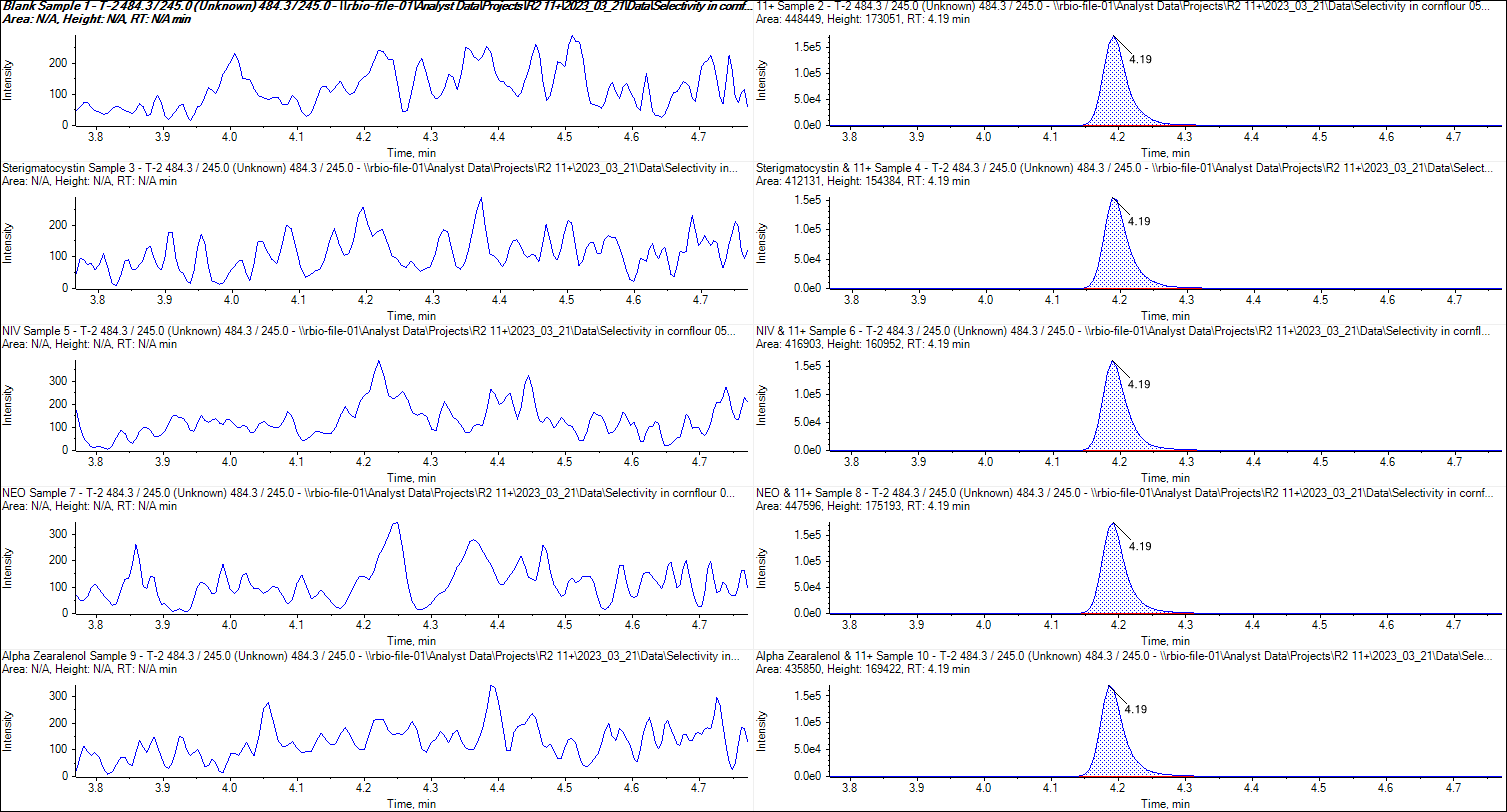
Component value 245.0


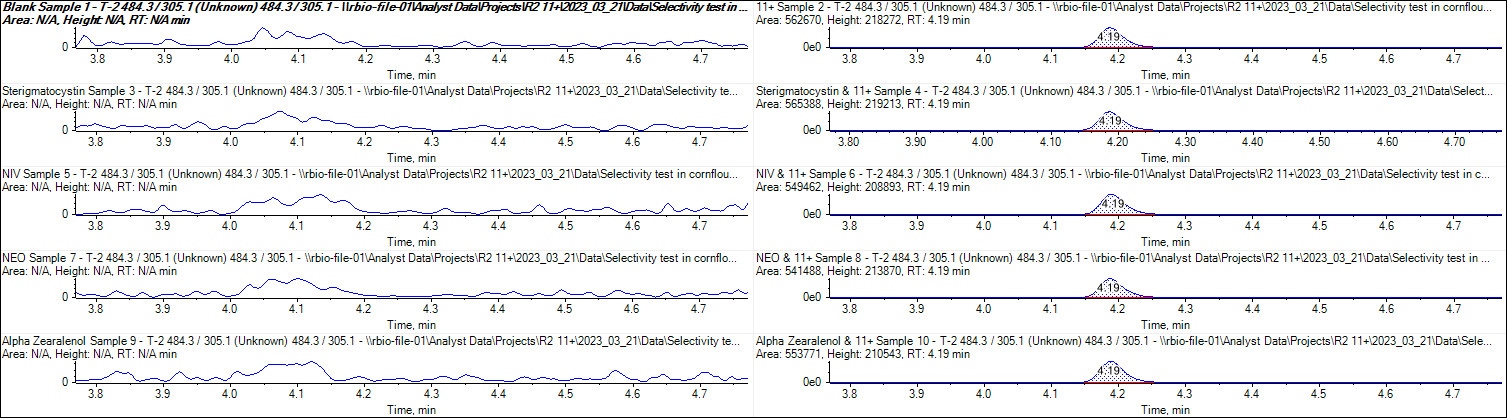


Component value 305.1

K Selectivity HT-2


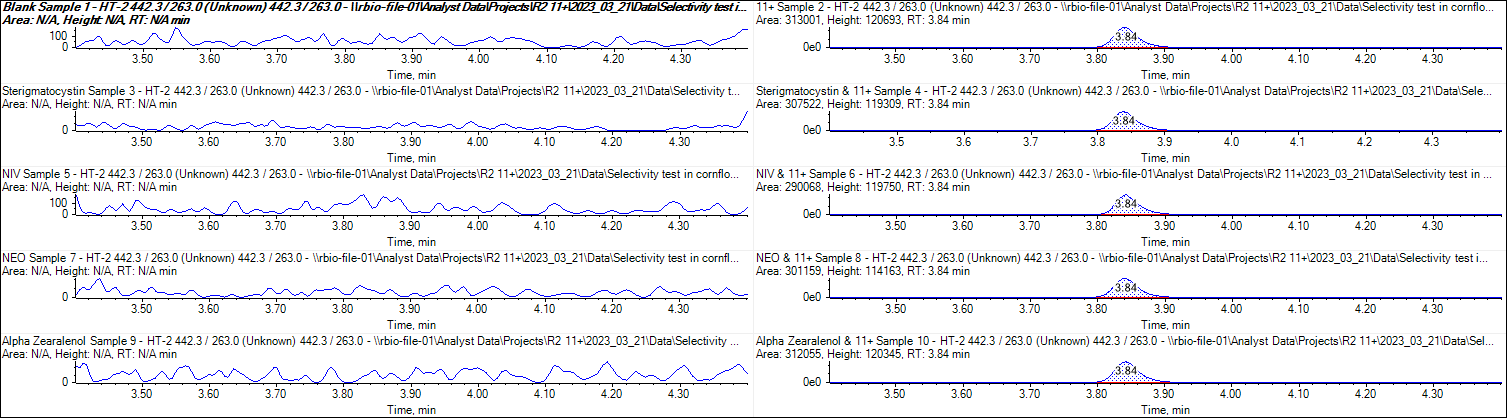


Component value 263.0


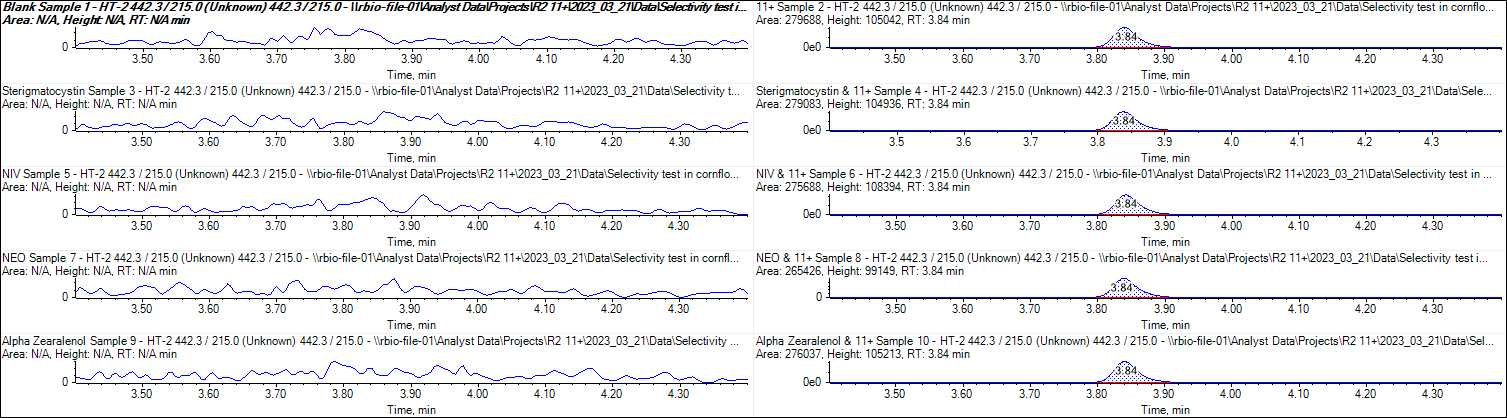


Component value 215.0

L Selectivity Zearalenone


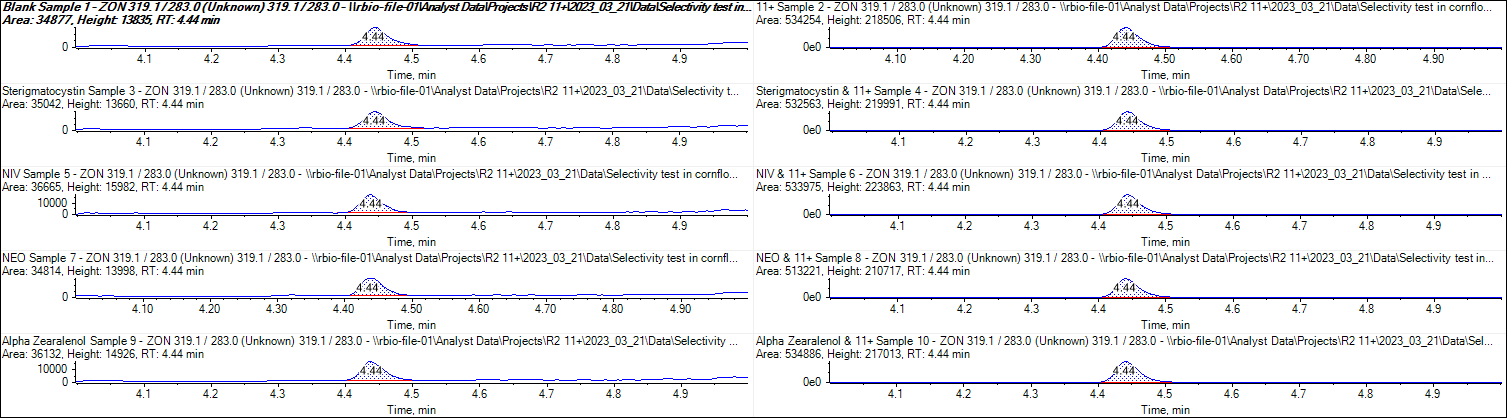


Component value 283.0


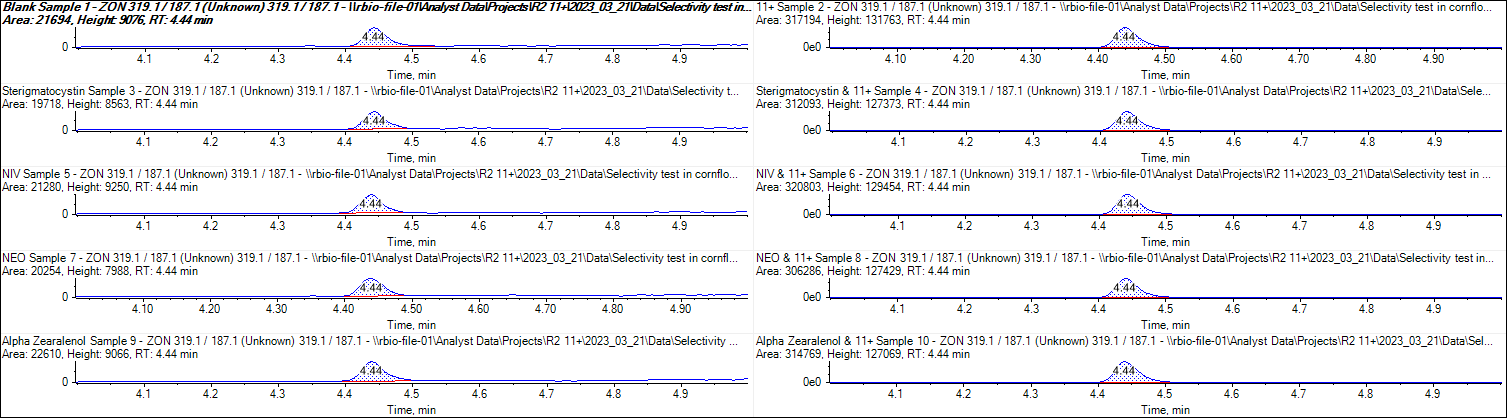


Component value 187.1

**Figure 3 Robustness Main Effects Plots**


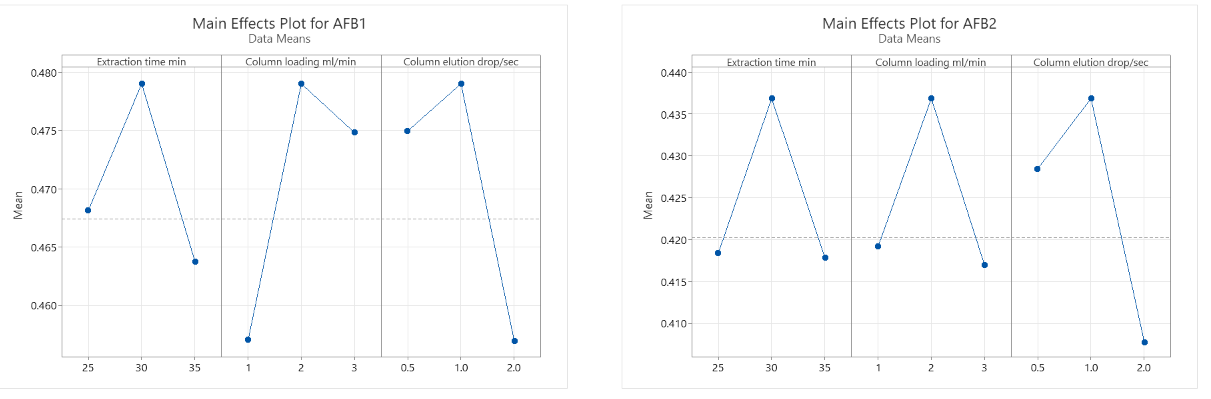

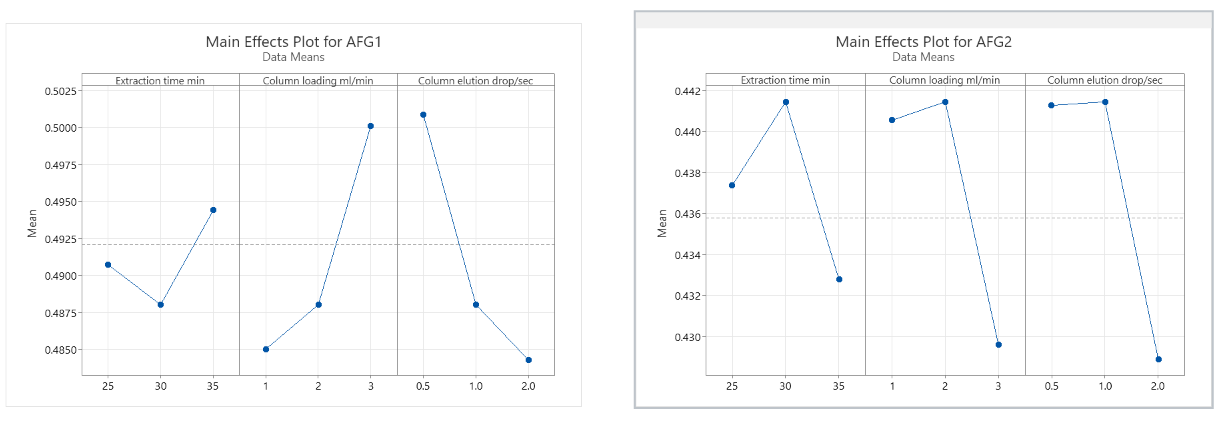


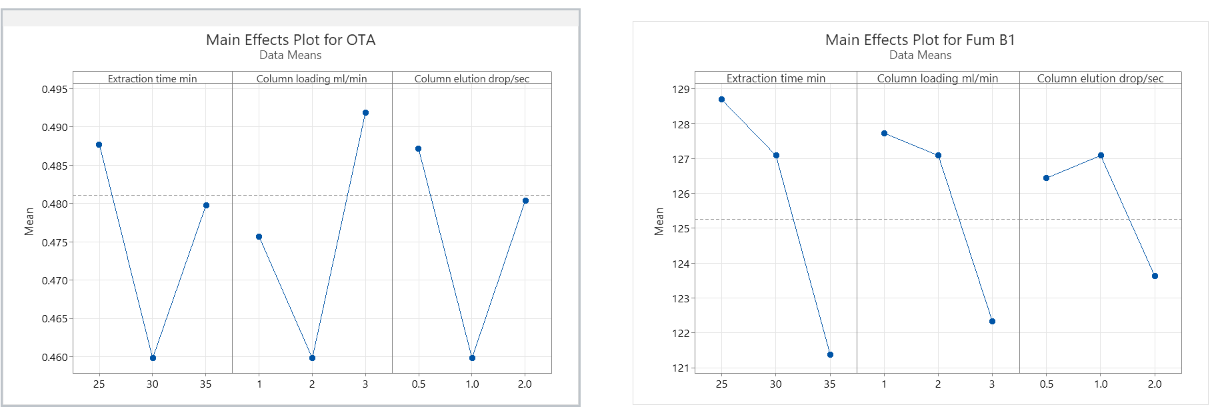


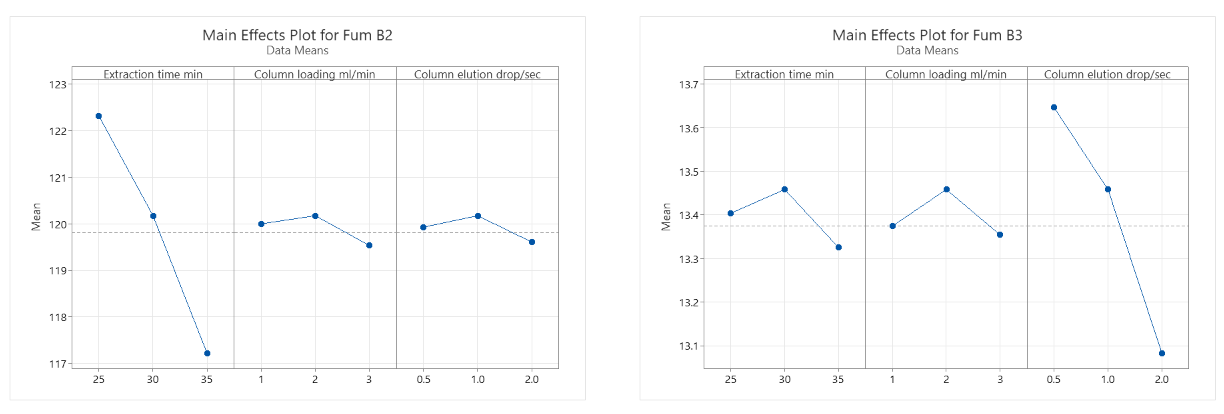


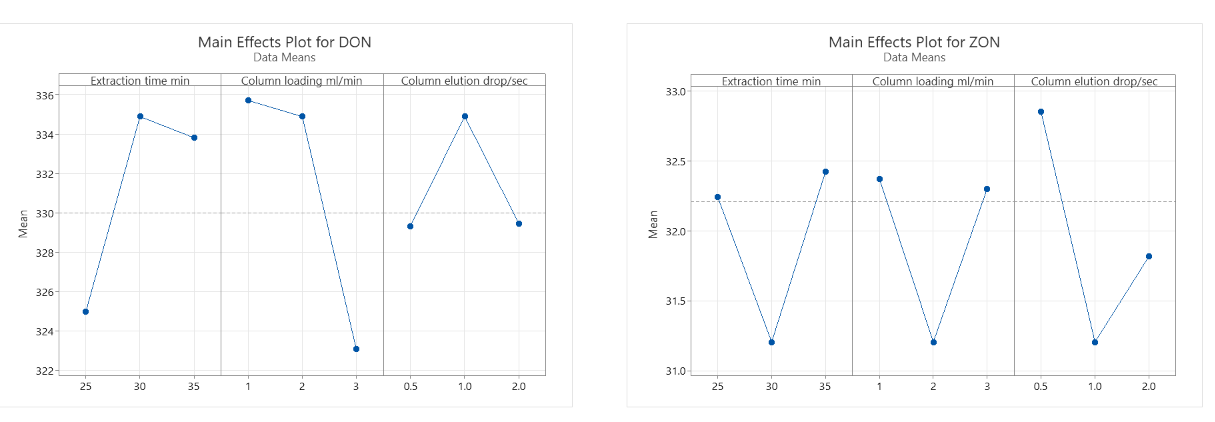


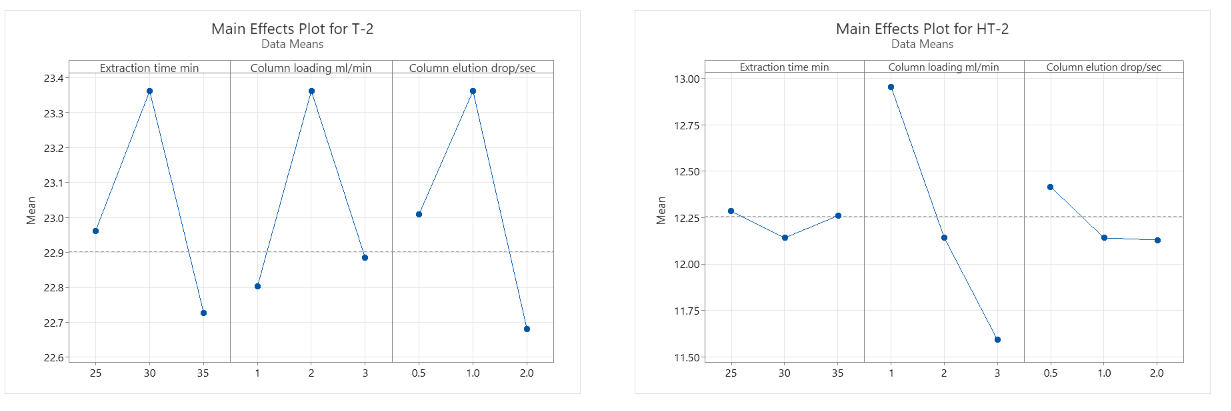


Figure 4 Robustness Interaction Plots


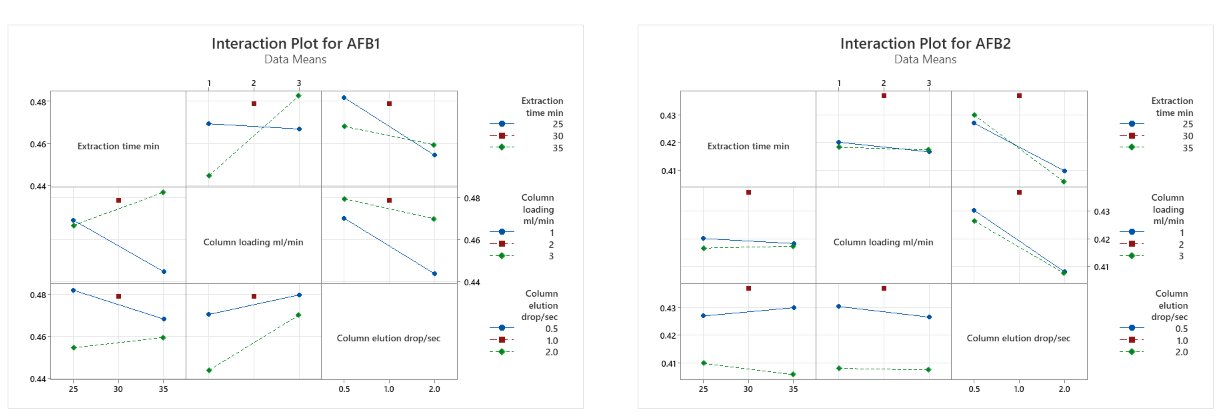


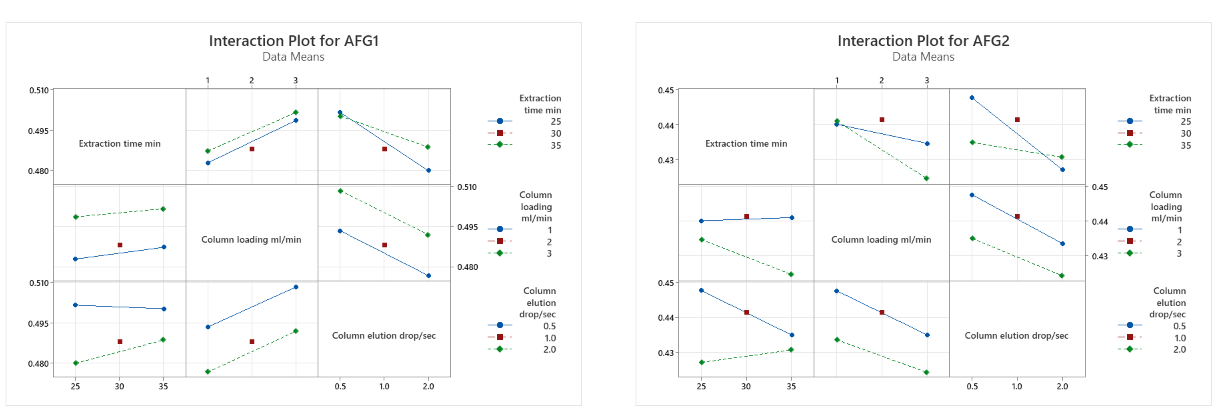


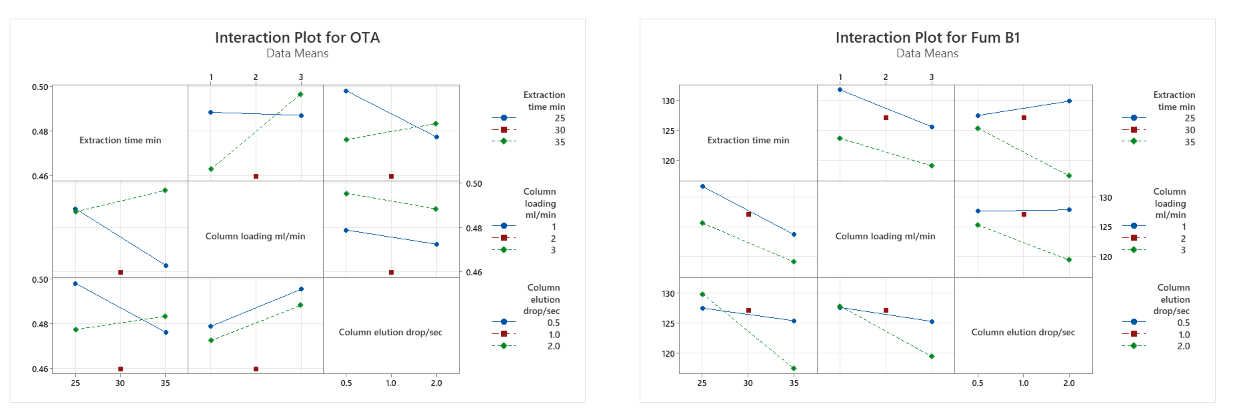


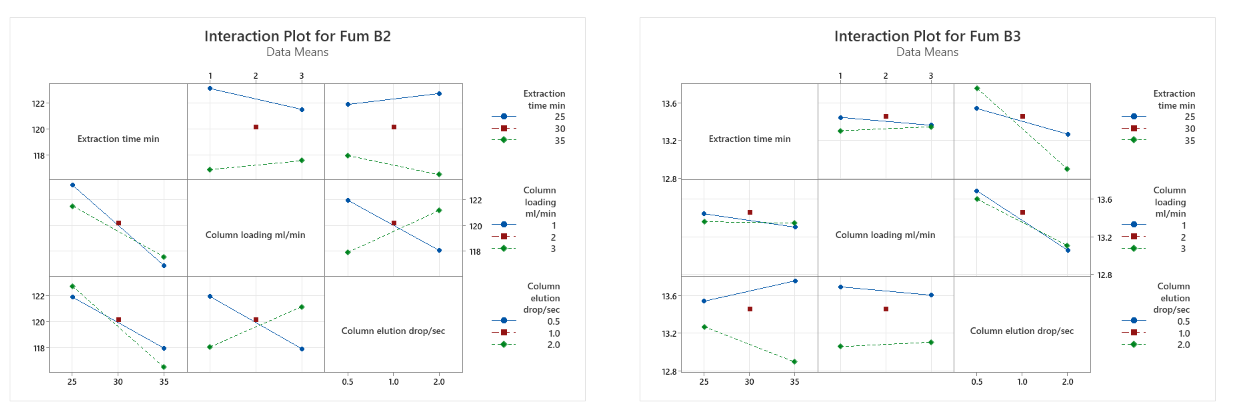


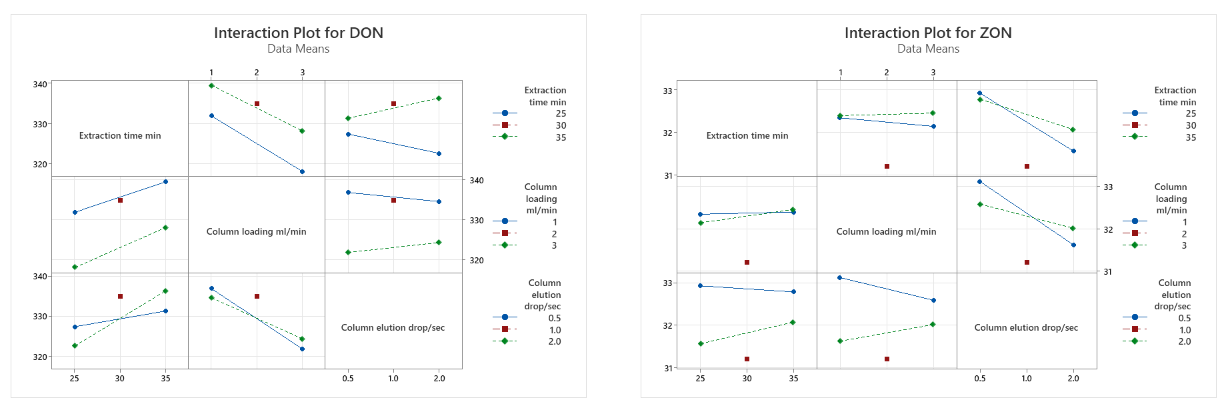


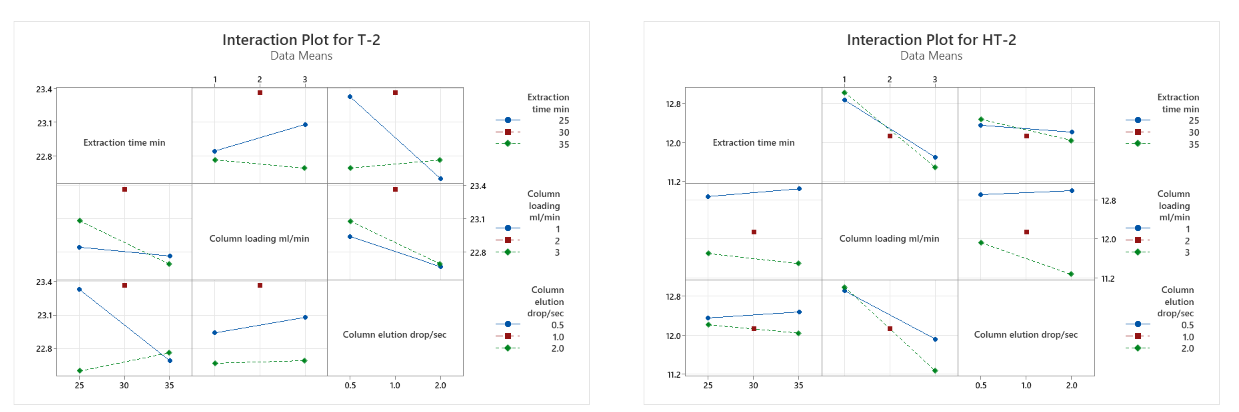


**Figure 5 Solvent Evaporation test summary report**

**Research & Development Interim Report**

**Title: Solvent Evaporation Test**

**Project: R^2**

**Author: Dave Leeman**

**Date: 6^th^ January 2023**

Objective

To assess extent of evaporation overnight of the different “spike solution diluents” with the different matrices. The spike solution diluents reflect the composition of spike solutions to be used in the study.

Matrix Information

Maize

Corn flour, Whole Food Earth

Wheat

Wheat grain, BuyWholeFoodsOnline, batch 20210628 QUE 724566

Baby food

Farley’s rusks reduced sugar, all ages 6 months onwards

Baby food (containing dairy ingredients)

Chocolate biscotti, 7+ months (Heinz product)

Paprika

Old India, batch MO56305/0013

Chilli

Schwartz Chilli powder

Animal feed

Mixed Corn, Small Holder Range

Sample Protocol

For all samples except animal feed 9 x 5 g sample was weighed into 50 mL centrifuge tubes.

To six of the aliquots 200 µL of the relevant Spike Solution Diluent (SSD) was added.

For animal feed 15 x 5 g sample was weighed out.

To six of the aliquots 200 µL of SSD was added.

To six of the aliquots 610 µL of SSD was added.

For samples where SSD is added

1. Put small plastic beaker onto 3 decimal place balance and add capless 50ml centrifuge tube. Weigh and record weight.
2. Add 5 g of sample, weigh and record weight.
3. Pipette required volume of the appropriate Spike Solution Diluent and weigh and record weight.
4. Leave overnight in a cupboard, uncovered and in the dark then weigh and record weight after around 18 hours.
5. Put back in cupboard, uncovered and in the dark then weigh and record weight after another 6 hours.

For samples where SSD is NOT added

1. Put small plastic beaker onto 3 decimal place balance and add capless 50ml centrifuge tube. Weigh and record weight.
2. Add 5 g of sample, weigh and record weight.
3. Leave overnight in a cupboard, uncovered and in the dark then reweigh and record weight after around 18 hours.
4. Put back in cupboard, uncovered and in the dark then weigh and record weight after another 6 hours.

The initial weighings were conducted in an afternoon with measurements at 18 hours weighings the following morning and the 24 hours later in the following afternoon. This would cover the expected time range after spiking that the extractions would occur.

Preparation of Sample Spike Diluents

SSD composition and amounts in applied SSD volumes are in tables below the preparation details.

**Preparation of Cereal SSD**

Add 8570 µL of methanol, 890 µL of acetonitrile and 540 µL of water to a 10 mL bottle. Mix well.

| % Solvent | | | Volume (µL) | | |
| --- | --- | --- | --- | --- | --- |
| MeOH | AcN | Water | MeOH | AcN | Water |
| 85.7 | 8.9 | 5.4 | 171.4 | 17.8 | 10.8 |

**Preparation of Cereal-Based Baby Food (CBBF) SSD**

Add 9724 µL of methanol, 168 µL of acetonitrile and 108 µL of water to a 10 mL bottle. Mix well.

| % Solvent | | | Volume (µL) | | |
| --- | --- | --- | --- | --- | --- |
| MeOH | AcN | Water | MeOH | AcN | Water |
| 97.2 | 1.7 | 1.1 | 194.5 | 3.4 | 2.2 |

**Preparation of Cereal-Based Baby Food with dairy ingredients (CBBF dairy) SSD**

Add 9672 µL of methanol, 220 µL of acetonitrile and 108 µL of water to a 10 mL bottle. Mix well.

| % Solvent | | | Volume (µL) | | |
| --- | --- | --- | --- | --- | --- |
| MeOH | AcN | Water | MeOH | AcN | Water |
| 96.7 | 2.2 | 1.1 | 193.4 | 4.4 | 2.2 |

**Preparation of Spices SSD**

Add 9434 µL of methanol, 458 µL of acetonitrile and 108 µL of water to a 10 mL bottle. Mix well.

| % Solvent | | | Volume (µL) | | |
| --- | --- | --- | --- | --- | --- |
| MeOH | AcN | Water | MeOH | AcN | Water |
| 94.3 | 4.6 | 1.1 | 188.7 | 9.2 | 2.2 |

**Preparation of Animal feed SSD**

Add 4505 µL of methanol, 2038 µL of acetonitrile and 777 µL of water to a 10 mL bottle. Mix well.

| Volume | % Solvent | | | Volume (µL) | | |
| --- | --- | --- | --- | --- | --- | --- |
|  | MeOH | AcN | Water | MeOH | AcN | Water |
| 200µl | 61.5 | 27.8 | 10.6 | 123.1 | 55.7 | 21.2 |
| 610µl | 61.5 | 27.8 | 10.6 | 375.4 | 169.8 | 64.8 |

Results

In provided excel workbook the recorded weights and calculated results are given in a series of tables.

Each matrix is covered in a worksheet with 4 tables.

One table gives the recorded weights for samples with addition of SSD.

An associated table shows the calculated values of sample weight, weight of applied SSD, weight of residual non-evaporated SSD after 18 and 24 hours and % of non-evaporated SSD after 18 and 24 hours. Mean and RSD values are given.

One table gives the recorded weights for samples with no addition of SSD.

An associated table shows the calculated values of sample weight and weight gain/loss after 18 and 24 hours. Mean and RSD values are given.

Brief summary

While most of the SSD evaporated there was still some residual non-evaporated SSD left after 24 hours in all cases.

There was less evaporation with the samples where the SSD had a higher water content, (cornflour, wheat and animal feed), than for SSD’s with a lower water content (Farley’s rusks, chocolate biscotti, paprika and chilli powder).

Results showed an increase in weight of the Farley’s rusks baby food over time which is assumed to be due to absorbing moisture from the atmosphere. This effect over both 18 and 24 hours was not seen with the other samples and led to a slightly anomalous increase in non-evaporated SSD weight from 18 to 24 hours in this sample.

We do not believe that the residual amounts observed would have any significant impact on the volume or composition of the extraction solvent.

As there was little difference in evaporation weights at 18 or 24 hours, 18 hours was deemed the more suitable time to leave overnight as this allowed spiking in the afternoon before starting early the next morning fitting better into the working day. This approach would be more practical for laboratory analysis.

**Figure 6 Solvent Evaporation test data**

Cornflour

|  | Weight 1 = Beaker + centrifuge tube | | |  |  |  |
| --- | --- | --- | --- | --- | --- | --- |
|  | Weight 2 = Beaker + centrifuge tube + 5 g sample | | |  |  |  |
|  | Weight 3 = Beaker + centrifuge tube + 5 g sample + SSD | | | |  |  |
|  | Weight 4 = Beaker + centrifuge tube + 5 g sample + SSD after 18 hours | | | |  |  |
|  | Weight 5 = Beaker + centrifuge tube + 5 g sample + SSD after 24 hours | | | |  |  |
|  |  |  |  |  |  |  |
| Cornflour 200 µL SSD addition | | | | | |  |
|  |  |  |  |  |  |  |
| Sample | Weight 1 (g) | Weight 2 (g) | Weight 3 (g) | Weight 4 (g) | Weight 5 (g) |  |
| 1 | 21.066 | 26.090 | 26.248 | 26.150 | 26.144 |  |
| 2 | 21.027 | 26.046 | 26.204 | 26.106 | 26.100 |  |
| 3 | 21.165 | 26.154 | 26.312 | 26.215 | 26.209 |  |
| 4 | 20.980 | 25.985 | 26.144 | 26.046 | 26.040 |  |
| 5 | 21.220 | 26.190 | 26.349 | 26.248 | 26.242 |  |
| 6 | 21.149 | 26.147 | 26.307 | 26.209 | 26.203 |  |
|  |  |  |  |  |  |  |

|  |  |  |  |  |  |  |  |
| --- | --- | --- | --- | --- | --- | --- | --- |
| Cornflour 200 µL SSD addition | | | | | | |  |
| Sample | Sample wt (g) | SSD wt (g) | Non-evap wt (g) 18hr | % Non-evap 18hr | Non-evap wt (g) 24hr | % Non-evap 24hr |  |
|  |  |  |  |  |  |  |  |
| 1 | 5.024 | 0.158 | 0.060 | 37.975 | 0.054 | 34.177 |  |
| 2 | 5.019 | 0.158 | 0.060 | 37.975 | 0.054 | 34.177 |  |
| 3 | 4.989 | 0.158 | 0.061 | 38.608 | 0.055 | 34.810 |  |
| 4 | 5.005 | 0.159 | 0.061 | 38.365 | 0.055 | 34.591 |  |
| 5 | 4.970 | 0.159 | 0.058 | 36.478 | 0.052 | 32.704 |  |
| 6 | 4.998 | 0.160 | 0.062 | 38.750 | 0.056 | 35.000 |  |
| Mean | 5.001 | 0.159 | 0.060 | 38.0 | 0.054 | 34.2 |  |
| SD | 0.020 | 0.001 | 0.001 | 0.8 | 0.001 | 0.8 |  |
| RSD | 0.4 | 0.5 | 2.3 | 2.2 | 2.5 | 2.4 |  |
|  | Weight 2 - Weight 1 | Weight 3 - Weight 2 | Weight 4 - Weight 2 |  | Weight 5 - Weight 2 |  |  |

|  | Weight 1 = Beaker + centrifuge tube | | |  |
| --- | --- | --- | --- | --- |
|  | Weight 2 = Beaker + centrifuge tube + 5 g sample | | |  |
|  | Weight 3 = Beaker + centrifuge tube + 5 g sample after 18 hours | | |  |
|  | Weight 4 = Beaker + centrifuge tube + 5 g sample after 24 hours | | |  |
|  |  |  |  |  |
| Cornflour no SSD addition | | | | |
|  |  |  |  |  |
| Sample | Weight 1 (g) | Weight 2 (g) | Weight 3 (g) | Weight 4 (g) |
| 1 | 20.822 | 25.790 | 25.789 | 25.795 |
| 2 | 21.164 | 26.143 | 26.143 | 26.148 |
| 3 | 20.981 | 26.016 | 26.017 | 26.022 |
|  |  |  |  |  |
|  |  |  |  |  |
| Cornflour no SSD addition | | | |  |
| Sample | Sample wt (g) | Weight gain/loss 18hr (g) | Weight gain/loss 24hr (g) |  |
|  |  |  |  |  |
| 1 | 4.968 | -0.001 | 0.005 |  |
| 2 | 4.979 | 0.000 | 0.005 |  |
| 3 | 5.035 | 0.001 | 0.006 |  |
|  | Weight 2 - Weight 1 | Weight 3 - Weight 2 | Weight 4 - Weight 2 |  |

Wheat

| Wheat 200 µL SSD addition | | | | | |  |
| --- | --- | --- | --- | --- | --- | --- |
|  |  |  |  |  |  |  |
| Sample | Weight 1 (g) | Weight 2 (g) | Weight 3 (g) | Weight 4 (g) | Weight 5 (g) |  |
| 1 | 21.049 | 26.074 | 26.231 | 26.113 | 26.110 |  |
| 2 | 21.083 | 26.089 | 26.245 | 26.129 | 26.127 |  |
| 3 | 21.003 | 26.022 | 26.179 | 26.062 | 26.060 |  |
| 4 | 20.866 | 25.885 | 26.042 | 25.926 | 25.923 |  |
| 5 | 20.833 | 25.819 | 25.978 | 25.859 | 25.858 |  |
| 6 | 21.238 | 26.249 | 26.406 | 26.291 | 26.288 |  |
|  |  |  |  |  |  |  |

|  |  |  |  |  |  |  |  |
| --- | --- | --- | --- | --- | --- | --- | --- |
| Wheat 200 µL SSD addition | | | | | | |  |
| Sample | Sample wt (g) | SSD wt (g) | Non-evap wt (g) 18hr | % Non-evap 18hr | Non-evap wt (g) 24hr | % Non-evap 24hr |  |
|  |  |  |  |  |  |  |  |
| 1 | 5.025 | 0.157 | 0.039 | 24.841 | 0.036 | 22.930 |  |
| 2 | 5.006 | 0.156 | 0.040 | 25.641 | 0.038 | 24.359 |  |
| 3 | 5.019 | 0.157 | 0.040 | 25.478 | 0.038 | 24.204 |  |
| 4 | 5.019 | 0.157 | 0.041 | 26.115 | 0.038 | 24.204 |  |
| 5 | 4.986 | 0.159 | 0.040 | 25.157 | 0.039 | 24.528 |  |
| 6 | 5.011 | 0.157 | 0.042 | 26.752 | 0.039 | 24.841 |  |
| Mean | 5.011 | 0.157 | 0.040 | 25.7 | 0.038 | 24.2 |  |
| SD | 0.014 | 0.001 | 0.001 | 0.7 | 0.001 | 0.7 |  |
| RSD | 0.3 | 0.6 | 2.6 | 2.7 | 2.9 | 2.7 |  |

| Wheat no SSD addition | | | | |
| --- | --- | --- | --- | --- |
|  |  |  |  |  |
| Sample | Weight 1 (g) | Weight 2 (g) | Weight 3 (g) | Weight 4 (g) |
| 1 | 20.976 | 25.992 | 25.987 | 25.991 |
| 2 | 20.820 | 25.834 | 25.830 | 25.835 |
| 3 | 20.873 | 25.873 | 25.868 | 25.874 |
|  |  |  |  |  |

|  |  |  |  |  |
| --- | --- | --- | --- | --- |
| Wheat no SSD addition | | | |  |
| Sample | Sample wt (g) | Weight gain/loss 18hr (g) | Weight gain/loss 24hr (g) |  |
|  |  |  |  |  |
| 1 | 5.016 | -0.005 | -0.001 |  |
| 2 | 5.014 | -0.004 | 0.001 |  |
| 3 | 5.000 | -0.005 | 0.001 |  |

Cereal based baby food (non dairy)

| Farley's rusks 200 µL SSD addition | | | | | |  |
| --- | --- | --- | --- | --- | --- | --- |
|  |  |  |  |  |  |  |
| Sample | Weight 1 (g) | Weight 2 (g) | Weight 3 (g) | Weight 4 (g) | Weight 5 (g) |  |
| 1 | 21.037 | 26.052 | 26.203 | 26.072 | 26.077 |  |
| 2 | 21.033 | 26.042 | 26.191 | 26.062 | 26.066 |  |
| 3 | 21.008 | 26.014 | 26.166 | 26.038 | 26.044 |  |
| 4 | 21.064 | 26.062 | 26.213 | 26.084 | 26.090 |  |
| 5 | 20.960 | 25.973 | 26.124 | 25.996 | 26.001 |  |
| 6 | 20.856 | 25.855 | 26.005 | 25.877 | 25.883 |  |
|  |  |  |  |  |  |  |

|  |  |  |  |  |  |  |  |
| --- | --- | --- | --- | --- | --- | --- | --- |
| Farley's rusks 200 µL SSD addition | | | | | | |  |
| Sample | Sample wt (g) | SSD wt (g) | Non-evap wt (g) 18hr | % Non-evap 18hr | Non-evap wt (g) 24hr | % Non-evap 24hr |  |
|  |  |  |  |  |  |  |  |
| 1 | 5.015 | 0.151 | 0.020 | 13.245 | 0.025 | 16.556 |  |
| 2 | 5.009 | 0.149 | 0.020 | 13.423 | 0.024 | 16.107 |  |
| 3 | 5.006 | 0.152 | 0.024 | 15.789 | 0.030 | 19.737 |  |
| 4 | 4.998 | 0.151 | 0.022 | 14.570 | 0.028 | 18.543 |  |
| 5 | 5.013 | 0.151 | 0.023 | 15.232 | 0.028 | 18.543 |  |
| 6 | 4.999 | 0.150 | 0.022 | 14.667 | 0.028 | 18.667 |  |
| Mean | 5.007 | 0.151 | 0.022 | 14.5 | 0.027 | 18.0 |  |
| SD | 0.007 | 0.001 | 0.002 | 1.0 | 0.002 | 1.4 |  |
| RSD | 0.1 | 0.7 | 7.3 | 6.9 | 8.2 | 7.7 |  |

| Farley's rusks no SSD addition | | | | |
| --- | --- | --- | --- | --- |
|  |  |  |  |  |
| Sample | Weight 1 (g) | Weight 2 (g) | Weight 3 (g) | Weight 4 (g) |
| 1 | 21.173 | 26.152 | 26.158 | 26.166 |
| 2 | 20.819 | 25.834 | 25.839 | 25.845 |
| 3 | 21.030 | 26.052 | 26.057 | 26.065 |
|  |  |  |  |  |

|  |  |  |  |  |
| --- | --- | --- | --- | --- |
| Farley's rusks no SSD addition | | | |  |
| Sample | Sample wt (g) | Weight gain/loss 18hr (g) | Weight gain/loss 24hr (g) |  |
|  |  |  |  |  |
| 1 | 4.979 | 0.006 | 0.014 |  |
| 2 | 5.015 | 0.005 | 0.011 |  |
| 3 | 5.022 | 0.005 | 0.013 |  |

Cereal based baby food (dairy)

| Chocolate biscotti 200 µL SSD addition | | | | | |  |
| --- | --- | --- | --- | --- | --- | --- |
|  |  |  |  |  |  |  |
| Sample | Weight 1 (g) | Weight 2 (g) | Weight 3 (g) | Weight 4 (g) | Weight 5 (g) |  |
| 1 | 21.398 | 26.405 | 26.556 | 26.426 | 26.423 |  |
| 2 | 21.104 | 26.113 | 26.266 | 26.134 | 26.132 |  |
| 3 | 21.263 | 26.279 | 26.431 | 26.299 | 26.298 |  |
| 4 | 21.199 | 26.195 | 26.346 | 26.215 | 26.214 |  |
| 5 | 21.549 | 26.529 | 26.681 | 26.549 | 26.547 |  |
| 6 | 21.348 | 26.329 | 26.481 | 26.350 | 26.348 |  |
|  |  |  |  |  |  |  |

|  |  |  |  |  |  |  |  |
| --- | --- | --- | --- | --- | --- | --- | --- |
| Chocolate biscotti 200 µL SSD addition | | | | | | |  |
| Sample | Sample wt (g) | SSD wt (g) | Non-evap wt (g) 18hr | % Non-evap 18hr | Non-evap wt (g) 24hr | % Non-evap 24hr |  |
|  |  |  |  |  |  |  |  |
| 1 | 5.007 | 0.151 | 0.021 | 13.907 | 0.018 | 11.921 |  |
| 2 | 5.009 | 0.153 | 0.021 | 13.725 | 0.019 | 12.418 |  |
| 3 | 5.016 | 0.152 | 0.020 | 13.158 | 0.019 | 12.500 |  |
| 4 | 4.996 | 0.151 | 0.020 | 13.245 | 0.019 | 12.583 |  |
| 5 | 4.980 | 0.152 | 0.020 | 13.158 | 0.018 | 11.842 |  |
| 6 | 4.981 | 0.152 | 0.021 | 13.816 | 0.019 | 12.500 |  |
| Mean | 4.998 | 0.152 | 0.020 | 13.5 | 0.019 | 12.3 |  |
| SD | 0.015 | 0.001 | 0.001 | 0.4 | 0.001 | 0.3 |  |
| RSD | 0.3 | 0.5 | 2.7 | 2.6 | 2.8 | 2.6 |  |

| Chocolate biscotti no SSD addition | | | | |
| --- | --- | --- | --- | --- |
|  |  |  |  |  |
| Sample | Weight 1 (g) | Weight 2 (g) | Weight 3 (g) | Weight 4 (g) |
| 1 | 21.249 | 26.238 | 26.244 | 26.245 |
| 2 | 21.087 | 26.062 | 26.065 | 26.065 |
| 3 | 21.202 | 26.228 | 26.231 | 26.232 |
|  |  |  |  |  |

|  |  |  |  |  |
| --- | --- | --- | --- | --- |
| Chocolate biscotti no SSD addition | | | |  |
| Sample | Sample wt (g) | Weight gain/loss 18hr (g) | Weight gain/loss 24hr (g) |  |
|  |  |  |  |  |
| 1 | 4.989 | 0.006 | 0.007 |  |
| 2 | 4.975 | 0.003 | 0.003 |  |
| 3 | 5.026 | 0.003 | 0.004 |  |

Paprika

| Paprika 200 µL SSD addition | | | | | |  |
| --- | --- | --- | --- | --- | --- | --- |
|  |  |  |  |  |  |  |
| Sample | Weight 1 (g) | Weight 2 (g) | Weight 3 (g) | Weight 4 (g) | Weight 5 (g) |  |
| 1 | 21.151 | 26.162 | 26.315 | 26.188 | 26.181 |  |
| 2 | 21.098 | 26.121 | 26.273 | 26.144 | 26.136 |  |
| 3 | 21.159 | 26.137 | 26.288 | 26.159 | 26.152 |  |
| 4 | 21.057 | 26.069 | 26.22 | 26.093 | 26.085 |  |
| 5 | 21.090 | 26.101 | 26.253 | 26.126 | 26.118 |  |
| 6 | 21.145 | 26.122 | 26.273 | 26.145 | 26.138 |  |
|  |  |  |  |  |  |  |

|  |  |  |  |  |  |  |  |
| --- | --- | --- | --- | --- | --- | --- | --- |
| Paprika 200 µL SSD addition | | | | | | |  |
| Sample | Sample wt (g) | SSD wt (g) | Non-evap wt (g) 18hr | % Non-evap 18hr | Non-evap wt (g) 24hr | % Non-evap 24hr |  |
|  |  |  |  |  |  |  |  |
| 1 | 5.011 | 0.153 | 0.026 | 16.993 | 0.019 | 12.418 |  |
| 2 | 5.023 | 0.152 | 0.023 | 15.132 | 0.015 | 9.868 |  |
| 3 | 4.978 | 0.151 | 0.022 | 14.570 | 0.015 | 9.934 |  |
| 4 | 5.012 | 0.151 | 0.024 | 15.894 | 0.016 | 10.596 |  |
| 5 | 5.011 | 0.152 | 0.025 | 16.447 | 0.017 | 11.184 |  |
| 6 | 4.977 | 0.151 | 0.023 | 15.232 | 0.016 | 10.596 |  |
| Mean | 5.002 | 0.152 | 0.024 | 15.7 | 0.016 | 10.8 |  |
| SD | 0.020 | 0.001 | 0.001 | 0.9 | 0.002 | 0.9 |  |
| RSD | 0.4 | 0.5 | 6.2 | 5.8 | 9.2 | 8.8 |  |

| Paprika no SSD addition | | | | |
| --- | --- | --- | --- | --- |
|  |  |  |  |  |
| Sample | Weight 1 (g) | Weight 2 (g) | Weight 3 (g) | Weight 4 (g) |
| 1 | 21.118 | 26.145 | 26.145 | 26.146 |
| 2 | 21.098 | 26.096 | 26.096 | 26.098 |
| 3 | 21.083 | 26.099 | 26.100 | 26.101 |
|  |  |  |  |  |

|  |  |  |  |  |
| --- | --- | --- | --- | --- |
| Paprika SSD addition | | | |  |
| Sample | Sample wt (g) | Weight gain/loss 18hr (g) | Weight gain/loss 24hr (g) |  |
|  |  |  |  |  |
| 1 | 5.027 | 0.000 | 0.001 |  |
| 2 | 4.998 | 0.000 | 0.002 |  |
| 3 | 5.016 | 0.001 | 0.002 |  |

Chili powder

| Chili 200 µL SSD addition | | | | | |  |
| --- | --- | --- | --- | --- | --- | --- |
|  |  |  |  |  |  |  |
| Sample | Weight 1 (g) | Weight 2 (g) | Weight 3 (g) | Weight 4 (g) | Weight 5 (g) |  |
| 1 | 21.031 | 26.058 | 26.209 | 26.083 | 26.075 |  |
| 2 | 20.835 | 25.859 | 26.010 | 25.886 | 25.878 |  |
| 3 | 20.837 | 25.861 | 26.013 | 25.887 | 25.880 |  |
| 4 | 20.964 | 25.995 | 26.146 | 26.021 | 26.014 |  |
| 5 | 20.886 | 25.895 | 26.046 | 25.919 | 25.913 |  |
| 6 | 21.173 | 26.181 | 26.332 | 26.207 | 26.201 |  |
|  |  |  |  |  |  |  |

|  |  |  |  |  |  |  |  |
| --- | --- | --- | --- | --- | --- | --- | --- |
| Chili 200 µL addition | | | | | | |  |
| Sample | Sample wt (g) | SSD wt (g) | Non-evap wt (g) 18hr | % Non-evap 18hr | Non-evap wt (g) 24hr | % Non-evap 24hr |  |
|  |  |  |  |  |  |  |  |
| 1 | 5.027 | 0.151 | 0.025 | 16.556 | 0.017 | 11.258 |  |
| 2 | 5.024 | 0.151 | 0.027 | 17.881 | 0.019 | 12.583 |  |
| 3 | 5.024 | 0.152 | 0.026 | 17.105 | 0.019 | 12.500 |  |
| 4 | 5.031 | 0.151 | 0.026 | 17.219 | 0.019 | 12.583 |  |
| 5 | 5.009 | 0.151 | 0.024 | 15.894 | 0.018 | 11.921 |  |
| 6 | 5.008 | 0.151 | 0.026 | 17.219 | 0.020 | 13.245 |  |
| Mean | 5.021 | 0.151 | 0.026 | 17.0 | 0.019 | 12.3 |  |
| SD | 0.010 | 0.000 | 0.001 | 0.7 | 0.001 | 0.7 |  |
| RSD | 0.2 | 0.3 | 4.0 | 4.0 | 5.5 | 5.5 |  |

| Chili no SSD addition | | | | |
| --- | --- | --- | --- | --- |
|  |  |  |  |  |
| Sample | Weight 1 (g) | Weight 2 (g) | Weight 3 (g) | Weight 4 (g) |
| 1 | 21.120 | 26.118 | 26.12 | 26.121 |
| 2 | 21.181 | 26.186 | 26.188 | 26.187 |
| 3 | 21.197 | 26.190 | 26.194 | 26.193 |
|  |  |  |  |  |

|  |  |  |  |  |
| --- | --- | --- | --- | --- |
| Chili SSD addition | | | |  |
| Sample | Sample wt (g) | Weight gain/loss 18hr (g) | Weight gain/loss 24hr (g) |  |
|  |  |  |  |  |
| 1 | 4.998 | 0.002 | 0.003 |  |
| 2 | 5.005 | 0.002 | 0.001 |  |
| 3 | 4.993 | 0.004 | 0.003 |  |

Animal Feed

| Animal feed 200 uL SSD addition | | | | | |  |
| --- | --- | --- | --- | --- | --- | --- |
|  |  |  |  |  |  |  |
| Sample | Weight 1 (g) | Weight 2 (g) | Weight 3 (g) | Weight 4 (g) | Weight 5 (g) |  |
| 1 | 21.141 | 26.176 | 26.336 | 26.228 | 26.218 |  |
| 2 | 20.948 | 25.963 | 26.122 | 26.013 | 26.004 |  |
| 3 | 20.803 | 25.839 | 26.000 | 25.887 | 25.878 |  |
| 4 | 20.971 | 25.964 | 26.124 | 26.012 | 26.003 |  |
| 5 | 21.135 | 26.115 | 26.275 | 26.162 | 26.154 |  |
| 6 | 20.943 | 25.958 | 26.119 | 26.012 | 26.003 |  |
|  |  |  |  |  |  |  |

|  |  |  |  |  |  |  |  |
| --- | --- | --- | --- | --- | --- | --- | --- |
| Animal feed 200 µL SSD addition | | | | | | |  |
| Sample | Sample wt (g) | SSD wt (g) | Non-evap wt (g) 18hr | % Non-evap 18hr | Non-evap wt (g) 24hr | % Non-evap 24hr |  |
|  |  |  |  |  |  |  |  |
| 1 | 5.035 | 0.160 | 0.052 | 32.500 | 0.042 | 26.250 |  |
| 2 | 5.015 | 0.159 | 0.050 | 31.447 | 0.041 | 25.786 |  |
| 3 | 5.036 | 0.161 | 0.048 | 29.814 | 0.039 | 24.224 |  |
| 4 | 4.993 | 0.160 | 0.048 | 30.000 | 0.039 | 24.375 |  |
| 5 | 4.980 | 0.160 | 0.047 | 29.375 | 0.039 | 24.375 |  |
| 6 | 5.015 | 0.161 | 0.054 | 33.540 | 0.045 | 27.950 |  |
| Mean | 5.012 | 0.160 | 0.050 | 31.1 | 0.041 | 25.5 |  |
| SD | 0.022 | 0.001 | 0.003 | 1.7 | 0.002 | 1.5 |  |
| RSD | 0.4 | 0.5 | 5.4 | 5.4 | 5.9 | 5.8 |  |

| Animal feed 610 uL SSD addition | | | | | |  |  |
| --- | --- | --- | --- | --- | --- | --- | --- |
|  |  |  |  |  |  |  |  |
| Sample | Weight 1 (g) | Weight 2 (g) | Weight 3 (g) | Weight 4 (g) | Weight 5 (g) |  |  |
| 1 | 20.810 | 25.841 | 26.332 | 26.008 | 25.979 |  |  |
| 2 | 21.005 | 26.021 | 26.515 | 26.194 | 26.164 |  |  |
| 3 | 21.156 | 26.159 | 26.651 | 26.330 | 26.296 |  |  |
| 4 | 21.027 | 26.033 | 26.522 | 26.204 | 26.170 |  |  |
| 5 | 20.994 | 26.015 | 26.507 | 26.190 | 26.160 |  |  |
| 6 | 20.879 | 25.882 | 26.376 | 26.051 | 26.018 |  |  |
|  | beaker + 50 ml tube | weight 1 + 5 g sample | Weight 2 + spike | Weight 3 after 18 hr | Weight 3 after 24hr |  |  |
|  |  |  |  |  |  |  |  |
| Animal feed 610 µL SSD addition | | | | | | |  |
| Sample | Sample wt (g) | SSD wt (g) | Non-evap wt (g) 18hr | % Non-evap 18hr | Non-evap wt (g) 24hr | % Non-evap 24hr |  |
|  |  |  |  |  |  |  |  |
| 1 | 5.031 | 0.491 | 0.167 | 34.012 | 0.138 | 28.106 |  |
| 2 | 5.016 | 0.494 | 0.173 | 35.020 | 0.143 | 28.947 |  |
| 3 | 5.003 | 0.492 | 0.171 | 34.756 | 0.137 | 27.846 |  |
| 4 | 5.006 | 0.489 | 0.171 | 34.969 | 0.137 | 28.016 |  |
| 5 | 5.021 | 0.492 | 0.175 | 35.569 | 0.145 | 29.472 |  |
| 6 | 5.003 | 0.494 | 0.169 | 34.211 | 0.136 | 27.530 |  |
| Mean | 5.013 | 0.492 | 0.171 | 34.8 | 0.139 | 28.3 |  |
| SD | 0.011 | 0.002 | 0.003 | 0.6 | 0.004 | 0.7 |  |
| RSD | 0.2 | 0.4 | 1.7 | 1.6 | 2.7 | 2.6 |  |

| Animal feed no SSD addition | | | | |
| --- | --- | --- | --- | --- |
|  |  |  |  |  |
| Sample | Weight 1 (g) | Weight 2 (g) | Weight 3 (g) | Weight 4 (g) |
| 1 | 21.219 | 26.201 | 26.203 | 26.203 |
| 2 | 20.822 | 25.857 | 25.857 | 25.856 |
| 3 | 20.961 | 25.946 | 25.945 | 25.946 |
|  |  |  |  |  |

|  |  |  |  |  |
| --- | --- | --- | --- | --- |
| Animal feed no SSD addition | | | |  |
| Sample | Sample wt (g) | Weight gain/loss 18hr (g) | Weight gain/loss 24hr (g) |  |
|  |  |  |  |  |
| 1 | 4.982 | 0.002 | 0.002 |  |
| 2 | 5.035 | 0.000 | -0.001 |  |
| 3 | 4.985 | -0.001 | 0.000 |  |

Summary of standard preparation

|  |  | Solvent (volume in µL) |  |  |  |  |  |
| --- | --- | --- | --- | --- | --- | --- | --- |
|  | Methanol | Acetonitrile | Water | Total vol (µL) | % MeOH | % ACN | % H2O |
| Cereals | 8570 | 890 | 540 | 10000 | 85.70 | 8.90 | 5.40 |
| CBBF non-dairy | 9724 | 168 | 108 | 10000 | 97.24 | 1.68 | 1.08 |
| CBBF dairy | 9672 | 220 | 108 | 10000 | 96.72 | 2.20 | 1.08 |
| Spices | 9434 | 458 | 108 | 10000 | 94.34 | 4.58 | 1.08 |
| AF | 4505 | 2038 | 777 | 7320 | 61.54 | 27.84 | 10.61 |

**Figure 7 Column capacity test experimental report**

**IR 11+ Myco MS-PREP Capacity**

**Title: Capacity Test**

**Project: PTM Program (11+ Myco MS-PREP)**

**Author: Dave Leeman**

Summary

11+ Myco MS-PREP columns were challenged at the proposed capacity levels in a loading solution with a solvent composition approximate to that used for the IFU cereal method. Acceptable recoveries (>70%) were obtained for Total Aflatoxin, DON, Total Fumonisin, Ochratoxin A, the sum of T-2 & HT-2, ZON.

Product & Standard Information

Product: 11+ Myco MS-PREP

Code: P128

Batch: LK 718

Expiry: 09 APR 25

Standard: 25 µg/mL Aflatoxin B1 (Actual concentration 25.8 µg/mL)

Lot: 240702-24247

Expiry: 03 SEP 25

Standard: 25 µg/mL Aflatoxin B2 (Actual concentration 25.3 µg/mL)

Lot: 240523-24247

Expiry: 03 SEP 25

Standard: 25 µg/mL Aflatoxin G1 (Actual concentration 25.4 µg/mL)

Lot: 240213-24247

Expiry: 03 SEP 25

Standard: 25 µg/mL Aflatoxin G2 (Actual concentration 25.1 µg/mL)

Lot: 240409-24247

Expiry: 03 SEP 25

Standard: 100 µg/mL DON (Actual concentration 98.2 µg/mL)

Lot: 240514-24247

Expiry: 03 SEP 25

Standard: 437.5 µg/mL Fumonisin (FB1:FB2:FB3 4:2:1)

(Actual concentrations: 428.5 µg/mL Fumonisin, 255 µg/mL FB1, 117 µg/mL FB2, 56.5 µg/mL FB3)

Lot: 230822-24183

Expiry: 01 JUL 25

Standard: 10 µg/mL OTA (Actual concentration 9.7 µg/mL)

Lot: 240401-24213

Expiry: 31 JUL 25

Standard: 100 µg/mL T-2 (Actual concentration 99.7 µg/mL)

Lot: 230405-24071

Expiry: 11 MAR 25

Standard: 100 µg/mL HT-2 (Actual concentration 98.7 µg/mL)

Lot: 230405-24071

Expiry: 20 MAR 25

Standard: 25 µg/mL ZON Stage 1 dilution (Actual concentration 24.839 µg/mL)

Lot: MB 803

Expiry: 07 FEB 25

Capacity Challenge Procedure

The selected spike values are shown below in ng.

The solvent composition of challenge solution was made so that it would approximate that of loading solution for the IFU cereal method (2% acetonitrile, 2% water and 96% PBS). Note: due to composition of stock standards used the actual loading solution was 2% acetonitrile, 0.82% methanol, 1.18% water and 96% PBS.

**Preparation of Capacity challenge solutions**

The volumes in the below table were used to make up the challenge solution. The volume of Capacity Working Standard (CWS) was added to the volume of acetonitrile in a 200ml glass amber bottle, then the volumes of water and PBS were added and mixed well.

**IAC column procedure**

Six columns were challenged with the Capacity challenge solution.

20 mL of Capacity spike challenge solution applied and passed through IAC at a flowrate of 2 mL per minute.

Column washed by passing 20 mL of 20 mM ammonium acetate wash solution through at a flowrate of approximately 5 mL per minute.

Toxins eluted with 1.5 mL methanol at a flowrate of 1 drop per second then 1.5 mL water passed through and collected in a 5 mL amber glass bottle.

1 mL of eluate was added to 9 mL of diluent and mixed well.

20 µL of diluted eluate injected onto LC-MS/MS system.

The amounts of injected dilute eluate equivalent to 100% recovery are given in Appendix 1.

Standard Preparation

Preparation of Capacity Working Standard (CWS)

To 500 µL of acetonitrile was added:

77.6 µL of 25.8 µg/mL Aflatoxin B1 standard

79.0µL of 25.3 µg/mL Aflatoxin B2 standard

78.8 µL of 25.4 µg/mL Aflatoxin G1 standard

79.6 µL of 25.1 µg/mL Aflatoxin G2 standard

362 µL of 98.2 µg/mL DON standard

249 µL of 428.5 µg/mL Total Fumonisin standard (FB1/FB2/FB3 4/2/1)

2,566 µL of 9.7 µg/mL Ochratoxin A standard

93.6 µL of 99,700 ng/mL T-2 standard

94.6 µL of 98,700 ng/mL HT-2 standard

573 µL of 24.839 µg/mL ZON standard

Made up 5 mL with acetonitrile and mixed well.

Preparation of calibration standards

A 6-point calibration curve was run. The curve was run before and after the spiked columns.

To make the curve the ng injected was plotted against peak area. The curve was set to Linear with a weighting of 1/x. For Fumonisin B1 the curve was set to Quadratic with a weighting of 1/x.

Calibration standard 6

200 µL of Capacity working standard was made up to 16 mL with diluent and mixed well.

Calibration standard 5

5 mL Calibration standard 6 added to 5 mL diluent and mixed well.

Calibration standard 4

5 mL Calibration standard 5 added to 5 mL diluent and mixed well.

Calibration standard 3

5 mL Calibration standard 4 added to 5 mL diluent and mixed well.

Calibration standard 2

5 mL Calibration standard 3 added to 5 mL diluent and mixed well.

Calibration standard 1

5 mL Calibration standard 2 added to 5 mL diluent and mixed well.

The standard concentrations and corresponding injected amounts for the individual analytes are given in Appendix 2.

Results summary

Calculated recoveries, mean recovery, SD, and RSD values are shown in the below table.

Conclusion

Recoveries of greater than 70% were obtained for Total Aflatoxin, DON, Total Fumonisin, Ochratoxin A, sum of T-2 & HT-2, ZON. It was decided that this confirmed the proposed capacity levels, and these would be stated in the IFU.

Appendix 1

**Spiked columns 100% recovery values (ng)**

| AFB1 | 0.075 |
| --- | --- |
| AFB2 | 0.075 |
| AFG1 | 0.075 |
| AFG2 | 0.075 |
| Tot AFT | 0.3 |
| DON | 1.33333333 |
| Fum B1 | 2.38039673 |
| Fum B2 | 1.09218203 |
| Fum B3 | 0.52742124 |
| Tot Fum | 4 |
| OTA | 0.93333333 |
| T-2 | 0.35 |
| HT-2 | 0.35 |
| T-2 & HT-2 | 0.7 |
| ZON | 0.53333333 |

Appendix 2

**Capacity test Standard concentrations (ng/ml)**

**Capacity test Standard injected amounts concentrations (ng)**
